# Molecular evolution of chloroplast genomes in subfamily Zingiberoideae (Zingiberaceae)

**DOI:** 10.1186/s12870-021-03315-9

**Published:** 2021-11-23

**Authors:** Dong-Mei Li, Jie Li, Dai-Rong Wang, Ye-Chun Xu, Gen-Fa Zhu

**Affiliations:** Guangdong Key Laboratory of Ornamental Plant Germplasm Innovation and Utilization, Environmental Horticulture Research Institute, Guangdong Academy of Agricultural Sciences, Guangzhou, 510640 China

**Keywords:** Zingiberaceae, Zingiberoideae, Chloroplast genome, Phylogeny, Divergent hotspots, Genome evolution

## Abstract

**Background:**

Zingiberoideae is a large and diverse subfamily of the family Zingiberaceae. Four genera in subfamily Zingiberoideae each possess 50 or more species, including *Globba* (100), *Hedychium* (*>* 80), *Kaempferia* (50) and *Zingiber* (150). Despite the agricultural, medicinal and horticultural importance of these species, genomic resources and suitable molecular markers for them are currently sparse.

**Results:**

Here, we have sequenced, assembled and analyzed ten complete chloroplast genomes from nine species of subfamily Zingiberoideae: *Globba lancangensis*, *Globba marantina*, *Globba multiflora*, *Globba schomburgkii*, *Globba schomburgkii var. angustata*, *Hedychium coccineum*, *Hedychium neocarneum*, *Kaempferia rotunda* ‘Red Leaf’, *Kaempferia rotunda* ‘Silver Diamonds’ and *Zingiber recurvatum*. These ten chloroplast genomes (size range 162,630–163,968 bp) possess typical quadripartite structures that consist of a large single copy (LSC, 87,172–88,632 bp), a small single copy (SSC, 15,393–15,917 bp) and a pair of inverted repeats (IRs, 29,673–29,833 bp). The genomes contain 111–113 different genes, including 79 protein coding genes, 28–30 tRNAs and 4 rRNA genes. The dynamics of the genome structures, gene contents, amino acid frequencies, codon usage patterns, RNA editing sites, simple sequence repeats and long repeats exhibit similarities, with slight differences observed among the ten genomes. Further comparative analysis of seventeen related Zingiberoideae species, 12 divergent hotspots are identified. Positive selection is observed in 14 protein coding genes, including *accD*, *ccsA*, *ndhA*, *ndhB*, *psbJ*, *rbcL*, *rpl20*, *rpoC1*, *rpoC2*, *rps12*, *rps18*, *ycf1*, *ycf2* and *ycf4.* Phylogenetic analyses, based on the complete chloroplast-derived single-nucleotide polymorphism data, strongly support that *Globba*, *Hedychium*, and *Curcuma* I + *“*the *Kaempferia* clade” consisting of *Curcuma* II, *Kaempferia* and *Zingiber*, form a nested evolutionary relationship in subfamily Zingiberoideae.

**Conclusions:**

Our study provides detailed information on ten complete Zingiberoideae chloroplast genomes, representing a valuable resource for future studies that seek to understand the molecular evolutionary dynamics in family Zingiberaceae. The identified divergent hotspots can be used for development of molecular markers for phylogenetic inference and species identification among closely related species within four genera of *Globba*, *Hedychium*, *Kaempferia* and *Zingiber* in subfamily Zingiberoideae.

**Supplementary Information:**

The online version contains supplementary material available at 10.1186/s12870-021-03315-9.

## Background

Zingiberaceae is a family of over 1200 species that span 53 genera [1–5]. These species are found throughout the tropical and subtropical world, with their primary populations and species diversity centered in Southern and Southeast Asia [1, 3–5]. The family Zingiberaceae consists of the four recognized subfamilies of Alpinioideae, Siphonochiloideae, Tamijioideae and Zingiberoideae, with *Globba, Hedychium*, *Kaempferia* and *Zingiber* belonging to Zingiberoideae [2]. These four genera each possess 50 or more species [1, 3–5]. Some of these species in subfamily Zingiberoideae have high economic value, such as *G. schomburgkii* and *H. coronarium*, ornamental and medicinal plants, *K. galanga*, a medicine and flavoring spice, and *Z. officinale*, an important crop and edible food [3–7].

It is difficult to identify Zingiberaceae plants merely based on their vegetative parts and flowers [2, 3, 5]. First, the vegetative parts of Zingiberaceae plants are often very similar, which is not suitable for species identification [3, 5]. Second, the structure of the flowers is complex, and the texture is weak, with some of them being as thin as cicada wings. Once pressed into dry specimens, it is difficult to know their original appearance, and they break and begin to decompose as soon as they are touched [5]. Past studies of Zingiberaceae have primarily concentrated on morphological classification and resources [1, 4, 5], ecology [4], cultivation and propagation [3, 4], medicinal and ornamental uses [3–7], and phylogeny [2, 8–11]. Among these phylogenetic investigations, several studies used nuclear internal transcribed spacer (*ITS*) and traditional chloroplast *matK/trnK-matK/trnL-trnF* data to explore the phylogenetic relationships within subfamily Zingiberoideae [2, 8] and within the four genera of *Globba*, *Hedychium*, *Kaempferia* and *Zingiber* [2, 9–11]. These traditional chloroplast markers have successfully identified the patterns of the evolutionary relationships within the four genera but in general, have been limited in differentiating resolutions among these four genera. Compared to traditional chloroplast markers, complete chloroplast genomes provide high resolution for relationship reconstruction within the *Alpinia*, *Amomum*, *Curcuma* and *Zingiber* genera, which allows an exploration of their phylogenetic positions in family Zingiberaceae [12–18]. Moreover, chloroplast single nucleotide polymorphism (SNP)-based phylogenetic analyses improve the phylogenetic resolution within the *Alpinia*, *Amomum*, *Curcuma, Hedychium*, *Kaempferia*, *Stahlianthus* and *Zingiber* genera in family Zingiberaceae [19–26]. However, because of the lack of complete chloroplast genomic data for the genus *Globba*, no studies have focused on the structural or mutational dynamics of the chloroplast genomes among the four genera of *Globba*, *Hedychium*, *Kaempferia* and *Zingiber* in subfamily Zingiberoideae.

Chloroplasts are important organelles that have their own genomes. They provide essential energy needed for plant growth and survival by converting light energy into carbohydrates through photosynthesis [27–29]. In angiosperms, chloroplast genomes typically consist of a large single copy region (LSC), a small single copy region (SSC), and two copies of inverted repeats (IRA and IRB) [27–29]. Most chloroplast genomes of flowering plants range in size from 107 kb (*Cathaya argyrophylla*) to 280 kb (*Pelargonium*) and consist of 110–130 genes, encoding ribosomal RNAs (rRNAs), transfer RNAs (tRNAs) and proteins [12–29]. In contrast with nuclear and mitochondrial genomes, chloroplast genomes are more conserved, shorter in length and more widely used in plant species identification and phylogenetic relationship analyses [12–29]. Moreover, the development of high-throughput sequencing technology has reduced the cost of sequencing and quickly accelerated comparative chloroplast genome and phylogenetic research.

In this study, we completely sequenced ten Zingiberoideae chloroplast genomes (*G. lancangensis*, *G. marantina*, *G. multiflora*, *G. schomburgkii*, *G. schomburgkii var. angustata*, *H. coccineum*, *H. neocarneum*, *K. rotunda* ‘Red Leaf’, *K. rotunda* ‘Silver Diamonds’ and *Z. recurvatum*) and compared them to eight other published chloroplast genomes from three genera, *Hedychium*, *Kaempferia* and *Zingiber*, within subfamily Zingiberoideae (*H. coronarium*, *H. spicatum*, *K. galanga*, *K. elegans*, *Z. montanum*, *Z. officinale*, *Z. spectabile* and *Z. zerumbet*), which were downloaded from GenBank. The primary aims of this study were as follows: (1) to compare and analyze the structure features of ten sequenced chloroplast genomes from four genera, *Globba*, *Hedychium*, *Kaempferia* and *Zingiber*; (2) to determine the sequence variation and molecular evolution among all 18 chloroplast genomes from the four genera in subfamily Zingiberoideae; and (3) to reconstruct phylogenetic relationships to verify the four genera’s relationships within subfamily Zingiberoideae.

## Results

### Features of ten sequenced Zingiberoideae chloroplast genomes

**Fig. 1:**
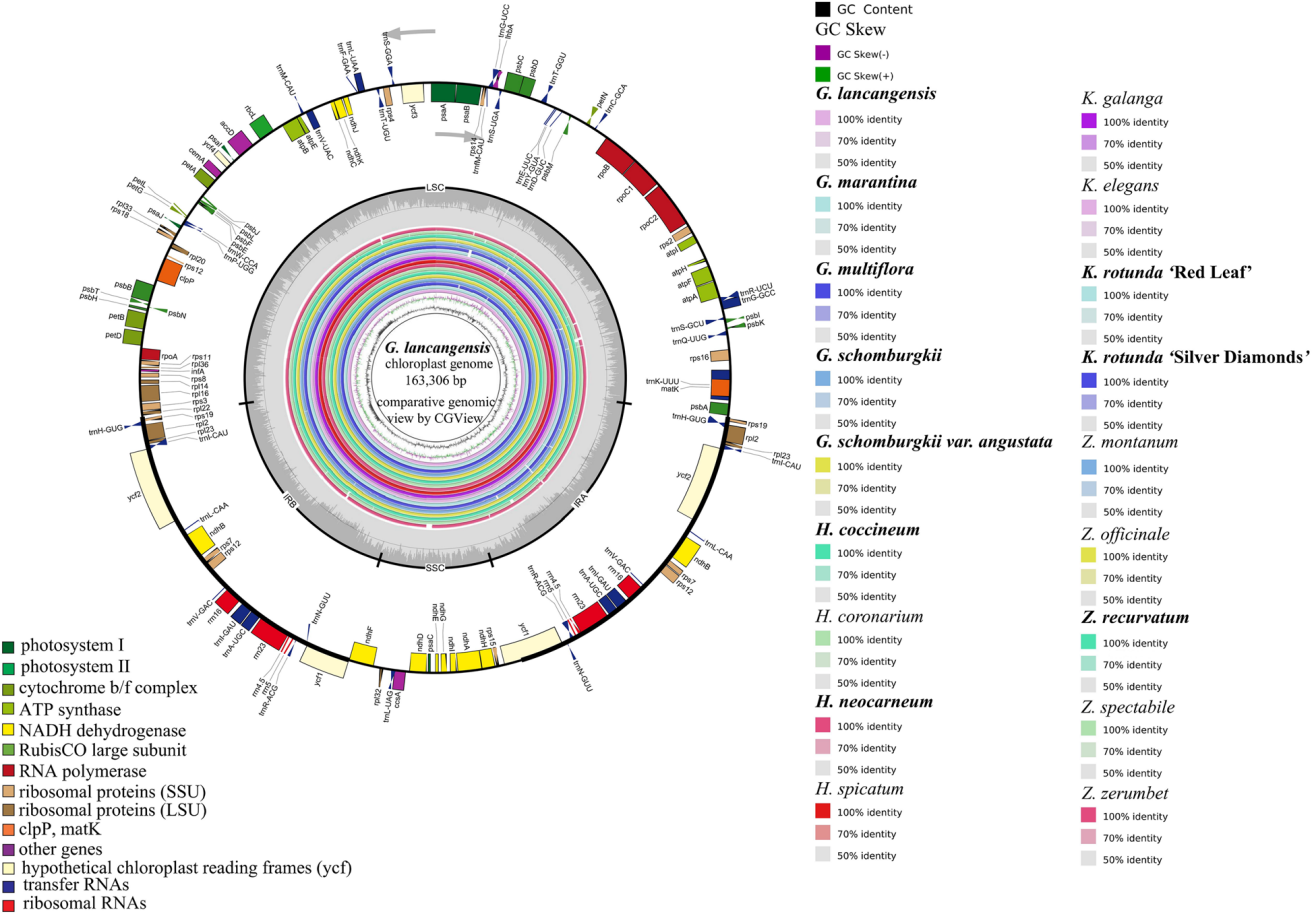
Chloroplast genome map of *G. lancangensis* (GenBank accession number: MT473704; the outermost three rings) and CGView comparison [31] of eighteen Zingiberoideae chloroplast genomes (the inter rings with different colors). Genes belonging to different functional groups are shown in different colors in the outermost first ring. Genes shown on the outside of the outermost first ring are transcribed counter-clockwise and on the inside clockwise.The gray arrowheads indicate the direction of the genes. The tRNA genes are indicated by one letter code of amino acids with anticodons. LSC, large single copy region; IR, inverted repeat; SSC, small single copy region. The outermost second ring with darker gray corresponds to GC content, whereas the outermost third ring with the lighter gray corresponds to AT content of *G. lancangensis* chloroplast genome by OGDRAW [30]. The innermost first black ring indicates the chloroplast genome size of *G. lancangensis*. The innermost second and third rings indicate GC content and GC skews deviations in chloroplast genome of *G. lancangensis*, respectively: GC skew + indicates G > C, and GC skew – indicates G < C. From innermost fourth color ring to outwards 21st ring in turn: *G. lancangensis* MT473704, *G. marantina* MT473705, *G. multiflora* MT473706, *G. schomburgkii* MK262735, *G. schomburgkii var. angustata* MT473707, *H. coccineum* MT473708, *H. coronarium* MK262736, *H. neocarneum* MT473709, *H. spicatum* NC_047248, *K. galanga* MK209001, *K. elegans* MK209002, *K. rotunda* ‘Red Leaf’ MT473710, *K. rotunda* ‘Silver Diamonds’ MT473711, *Z. montanum* MK262727, *Z. officinale* NC_044775, *Z. recurvatum* MT473712, *Z. spectabile* JX088661 and *Z. zerumbet* MK262726; chloroplast genome similar and highly divergent locations are represented by continuous and interrupted track lines, respectively. The sequenced species studied here were marked in bold

All ten sequenced Zingiberoideae chloroplast genomes have a typical quadripartite structure containing one large single copy (LSC), one small single copy (SSC) and two inverted repeat regions (IRA and IRB) by OGDRAW [30] and CGView tool [31] (Fig. 1, Fig. S1, Table 1). The ten sequenced Zingiberoideae chloroplast genomes size ranges from 162,630 bp (*K. rotunda* ‘Red Leaf’) to 163,968 bp (*H. coccineum*) (Table 1, Table S1, Fig. S1). They display four junction regions, namely, a separate LSC region of 87,172–88,632 bp, an SSC region of 15,393–15,917 bp, and a pair of IRs (IRA and IRB) of 29,673–29,833 bp each (Fig. 1, Fig. S1, Table 1, Table S1). The size of the *G. schomburgkii var. angustata* chloroplast genome (163,432 bp) is the largest among the five sequenced *Globba* species, with 126 bp, 658 bp, 233 bp, and 107 bp longer than *G. lancangensis*, *G. marantina*, *G. multiflora* and *G. schomburgkii*, respectively (Table 1, Table S1). The GC content of these 10 chloroplast genomes is very similar (35.73–36.18%) (Table 1, Table S1). Specifically, the GC content in the IR regions (41.02–41.15%) is higher than that in the SSC regions (28.83–29.60%) and LSC regions (33.35–34.02%) (Table 1, Table S1). Additionally, the GC content at the third codon position (28.49–34.05%) is lower than that at the first (41.20–44.90%) and second (34.15–38.89%) positions in the protein coding genes of these 10 chloroplast genomes (Table S1). The ten sequenced chloroplast genomes contain 140–141 predicted functional genes, which consist of 87 protein coding genes, 45–46 tRNA genes, and 8 rRNA genes (Tables 1, 2, Table S2). Among these genes, a total of 111–113 different genes are detected in our 10 sequenced chloroplast genomes, including 79 protein coding genes, 28–30 tRNA genes, and 4 rRNA genes (Tables 1, 2, Table S2). Among the different protein coding genes in our 10 sequenced chloroplast genomes, 61 genes are located in the LSC regions, 12 genes are located in the SSC regions, and 8–9 genes are duplicated in the IR regions (Table 1, Table S2). Furthermore, most of the protein coding genes and rRNAs in our 10 sequenced Zingiberoideae chloroplast genomes are similar, but there are slight differences. For instance, the chloroplast genomes of *H. coccineum* and *H. neocarneum* have the *psbZ* gene, while *lhbA* gene is missing in both genomes (Table 2, Fig. S1). Additionally, for tRNAs, the chloroplast genome of *G. schomburgkii* has two copies of *trnS-GCU* and *trnT-UGU*, respectively, while the *trnS-GGA* and *trnT-GGU* are missing; the *trnS-GCU* and *trnT-UGU* exist as single copies in the remaining 9 sequenced Zingiberoideae chloroplast genomes (Table 2, Fig. S1).

**Table 1:** Comparison of ten chloroplast genomes features among the nine Zingiberoideae species studied

Genome feature	*G. lancangensis*	*G. marantina*	*G. multiflora*	*G. schomburgkii*	*G. schomburgkii var. angustata*	*H. coccineum*	*H. neocarneum*	*K. rotunda* ‘Red Leaf’	*K. rotunda* ‘Silver Diamonds’	*Z. recurvatum*
Genome size (bp)	163,306	162,774	163,199	163,325	163,432	163,968	163,903	162,630	162,875	163,151
LSC length (bp)	88,545	87,989	87,994	88,451	88,556	88,632	88,541	87,172	87,306	87,780
SSC length (bp)	15,393	15,425	15,715	15,525	15,526	15,798	15,824	15,800	15,917	15,787
IR length (bp)	29,684	29,680	29,745/ 29,742	29,673/ 29,676	29,675/ 29,678	29,769	29,769	29,829/ 29,833	29,826	29,792
GC content (%)										
Total genome	35.73	35.92	35.85	35.85	35.83	36.08	36.08	36.18	36.13	36.12
LSC	33.35	33.58	33.60	33.51	33.47	33.83	33.85	34.02	33.97	33.91
SSC	29.07	29.40	28.83	29.17	29.16	29.56	29.51	29.60	29.44	29.53
IR	41.02/41.03	41.07	41.02/41.03	41.09/41.10	41.09/41.10	41.15	41.15	41.08/41.09	41.08/41.09	41.14
CDS	36.59	36.68	36.68	36.68	36.68	37.22	37.22	36.94	36.96	36.95
Genes (total/different)	141/113	140/113	140/113	141/111	140/113	141/113	141/113	140/113	140/113	140/113
CDS (total/different)	87/79	87/79	87/79	87/79	87/79	87/79	87/79	87/79	87/79	87/79
tRNA (total/different)	46/30	45/30	45/30	46/28	45/30	46/30	46/30	45/30	45/30	45/30
rRNA (total/different)	8/4	8/4	8/4	8/4	8/4	8/4	8/4	8/4	8/4	8/4
Genes with introns	18	17	17	18	17	17	17	17	17	17
Different CDS in LSC	61	61	61	61	61	61	61	61	61	61
Different CDS in SSC	12	12	12	12	12	12	12	12	12	12
Different CDS in IRA	9	8	8	9	8	8	8	9	8	8
Different CDS in IRB	8	8	8	9	8	8	8	8	8	8
Different genes in IRs	21	20	20	20	20	20	20	21	20	20
GenBank accession	MT473704	MT473705	MT473706	MK262735	MT473707	MT47308	MT473709	MT473710	MT473711	MT473712

Note: *LSC* large single copy region, *SSC* small single copy region, *IR* inverted repeat, *CDS* protein coding genes

**Table 2:** Genes present in the ten sequenced chloroplast genomes in subfamily Zingiberoideae

Category for genes	Group of genes	Name of genes
Photosynthesis	Subunits of photosystem I	*psaA*, *psaB*, *psaC*, *psaI*, *psaJ*
	Subunits of photosystem II	*psbA*, *psbB*, *psbC*, *psbD*, *psbE*, *psbF*, *psbH*, *psbI*, *psbJ*, *psbK*, *psbL*, *psbM*, *psbN*, *psbT*, ① *psbZ,* ② *lhbA*
	Subunits of cytochrome b/f complex	*petA*, petB*, petD*, *petG*, *petL*, *petN*
	Subunits of ATP synthase	*atpA*, *atpB*, *atpE*, atpF*, *atpH*, *atpI*
	Subunits of NADH dehydrogenase	ndhA*, *ndhB* (×2)*, *ndhC*, *ndhD*, *ndhE*, *ndhF*, *ndhG*, *ndhH*, *ndhI*, *ndhJ*, *ndhK*
	Subunit of rubisco	*rbcL*
Self-replication	RNA polymerase	*rpoA*, *rpoB*, rpoC1*, *rpoC2*
	Large subunit of ribosomal proteins	*rpl2* (×2)*, *rpl14*, rpl16*, *rpl20*, *rpl22*, *rpl23* (× 2), *rpl32*, *rpl33*, *rpl36*
	Small subunit of ribosomal proteins	*rps2*, *rps3*, *rps4*, *rps7* (×2), *rps8*, *rps11*, *rps12* (× 2)*, *rps14*, *rps15*, rps16*, *rps18*, *rps19* (× 2)
	Ribosomal RNAs	*rrn4.5* (×2), *rrn5* (× 2), *rrn16* (× 2), *rrn23* (× 2)
	Transfer RNAs	*trnA-UGC* (×4)*, *trnC-GCA*, *trnD-GUC*, *trnE-UUC*, *trnF-GAA*, *trnfM-CAU*, *trnG-GCC* (×2)*, *trnG-UCC*, *trnH-GUG* (× 2), *trnI-CAU* (× 2), *trnI-GAU* (×4)*, *trnK-UUU* (× 2)*, *trnL-CAA* (× 2), *trnL-UAA* (× 2)*, *trnL-UAG*, *trnM-CAU*, *trnN-GUU* (× 2), *trnP-UGG*, *trnQ-UUG*, *trnR-ACG* (× 2), *trnR-UCU*, ③ *trnS-GCU* (× 2), ④ *trnS-GGA*, *trnS-UGA*, ④ *trnT-GGU,* ③ *trnT-UGU* (× 2), *trnV-GAC* (× 2), *trnV-UAC* (× 2)*, *trnW-CCA*, *trnY-GUA*
Other genes	Subunit of acetyl-coA-carboxylase	accD*
	c-type cytochrome synthesis gene	*ccsA*
	Envelop membrane protein	*cemA*
	Protease	clpP**
	Translational initiation factor	*infA*
	Maturase	*matK*
Unknown function	Conserved open reading frames	*ycf1* (×2), *ycf2* (×2), ycf3**, *ycf4*

Note: (×2): gene with two copies; (×4): gene with four copies; *: gene containing one intron; **: gene containing two introns; ①: *psbZ* gene is only present in the chloroplast genomes of *H. neocarneum* and *H. coccineum*, respectively; ②: *lhbA* gene is missing in the chloroplast genomes of *H. neocarneum* and *H. coccineum*, respectively; ③: *trnS-GCU* and *trnT-UGU* exist two gene copies only in the chloroplast genome of *G. schomburgkii*, and only once in other 9 sequenced chloroplast genomes in this study; ④: *trnS-GGA* and *trnT-GGU* are missing in the chloroplast genome of *G. schomburgkii*

As shown in Table 1, both *G. lancangensis* and *K. rotunda* ‘Red Leaf’ contain 21 genes in two IR regions, including 9 protein coding genes (*ndhB*, *ndhF*, *rpl2*, *rpl23*, *rps7*, *rps12*, *rps19*, *ycf1* and *ycf2*), 8 tRNA genes (*trnA-UGC*, *trnH-GUG*, *trnI-CAU*, *trnI-GAU*, *trnL-CAA*, *trnN-GUU*, *trnR-ACG* and *trnV-GAC*), and all four rRNAs (*rrn4.5*, *rrn5*, *rrn16* and *rrn23*). The other 8 chloroplast genomes contain 20 genes in the two IR regions, which is the same as the *G. lancangensis* and *K. rotunda* ‘Red Leaf’ chloroplast genomes, with the exception of *ndhF* gene (Table 1, Table S2).

A total of 18 genes contain introns in the chloroplast genomes of *G. lancangensis* and *G. schomburgkii*. Sixteen genes (*atpF*, *ndhA*, *ndhB*, *petB*, *petD*, *rpl2*, *rpl16*, *rpoC1*, *rps12*, *rps16*, *trnA-UGC*, *trnG-GCC*, *trnI-GAU*, *trnK-UUU*, *trnL-UAA*, and *trnV-UAC*) contain one intron, while *clpP* and *ycf3* each contains two introns (Table 2, Table S3). Among the 18 intron-containing genes in the chloroplast genomes of *G. lancangensis* and *G. schomburgkii*, four genes (*ndhB*, *rpl2*, *trnA-UAC* and *trnI-GAU*) occur in both IRs, 12 genes (*atpF*, *clpP*, *petB*, *petD*, *rpl16*, *rpoC1*, *rps16*, *trnG-GCC*, *trnL-UAA*, *trnK-UUU*, *trnV-UAC* and *ycf3*) are distributed in the LSC, one gene (*ndhA*) is in the SSC, and one gene’s (*rps12*) first exon is located in the LSC with the other two exons in both IRs (Table S3). The other 8 sequenced chloroplast genomes all contain 17 intron-containing genes (Table S3). Among the 17 intron-containing genes in these 8 chloroplast genomes, fifteen genes (*atpF*, *ndhA*, *ndhB*, *petB*, *petD*, *rpl2*, *rpl16*, *rpoC1*, *rps12*, *rps16*, *trnA-UGC*, *trnI-GAU*, *trnL-UAA*, *trnK-UUU* and *trnV-UAC*) contain one intron, while *clpP* and *ycf3* contain two introns each (Table S3). The locations of these 17 genes are the same as in the two chloroplast genomes of *G. lancangensis* and *G. schomburgkii*.

### Analyses of codon usage and predicted RNA editing sites

**Fig. 2:**
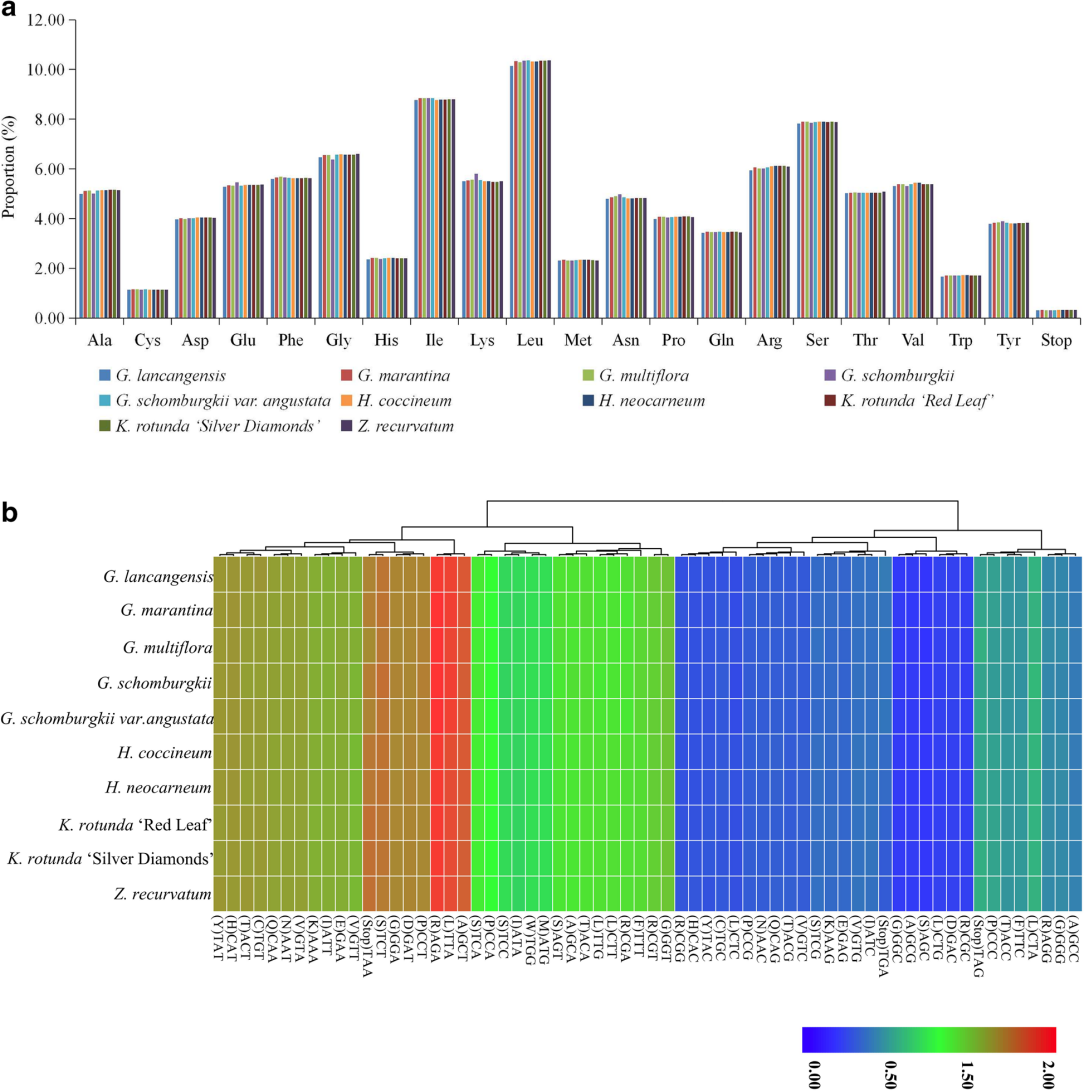
Codon content of all protein coding genes of ten sequenced chloroplast genomes in subfamily Zingiberoideae. **a** amino acids and stop codons proportion in protein coding sequences of ten sequenced chloroplast genomes and **b** heat map analysis for codon distribution of all protein coding genes of ten sequenced chloroplast genomes. Red colour indicates higher RSCU [32, 33] values and blue colour indicates lower RSCU values

A total of 79 protein coding genes in all 10 sequenced chloroplast genomes in subfamily Zingiberoideae are analyzed for codon usage frequency. They comprise 26,400 (*G. schomburgkii var. angustata*) to 27,730 (*G. schomburgkii*) codons. Of the 26,400–27,730 codons, leucine (Leu) is the most abundant amino acid, with a frequency of 10.14–10.69%, followed by isoleucine (Ile) with a frequency of 8.77–8.80%, while cysteine (Cys) is the least common, with a frequency of 1.13–1.15% (Fig. 2a, Table S4). This phenomenon is consistent with other Zingiberaceae plant chloroplast genomes, such as *Z. officinale* [15], *K. galanga* [18], *A. oxyphylla* [19] and *A. pumila* [19]. Because of the value of relative synonymous codon usage (RSCU) > 1.00, thirty codons show codon usage bias in the 10 sequenced chloroplast genomes’ protein coding genes (Fig. 2b, Table S5). Interestingly, out of the above 30 codons, twenty-nine are A/T-ending codons (Fig. 2b, Table S5). Conversely, C/G-ending codons have RSCU values of less than one, which indicates that they are less common in the 10 sequenced chloroplast genomes’ genes. Stop codon usage is biased toward TAA (RSCU > 1.00) (Fig. 2b, Table S5). Both methionine (Met) and tryptophan (Trp) exhibit no codon bias and have RSCU values of 1.00 (Fig. 2b, Table S5).

Furthermore, a total of 61 editing sites in 22 protein coding genes are identified in *G. schomburgkii*, while more numbers are found in *G. lancangensis* (76 sites), *G. marantina* (80 sites), *G. multiflora* (79 sites), *G. schomburgkii* var. *angustata* (78 sites), *H. coccineum* (79 sites), *H. neocarneum* (79 sites), *K. rotunda* ‘Red Leaf’ (81 sites), *K. rotunda* ‘Silver Diamonds’ (81 sites), and *Z. recurvatum* (80 sites) (Table S6). In the 10 identified chloroplast genomes that we sequenced, the *ndhB* gene has the highest number of potential editing sites (11), followed by the *ndhD* gene (9) (Table S6). Similar to other Zingiberaceae species, such as *K. galanga* [18], *A. pumila* [19], and *Z. zerumbet* [26], the *ndhB* gene contains the highest number of editing sites. All of these editing sites are C-to-T transitions that occur at the first or second positions of the codons. Interestingly, most RNA editing sites lead to amino acid changes for hydrophobic products, such as leucine, isoleucine, tryptophan, tyrosine, valine, methionine, and phenylalanine (Table S6). Similar RNA editing features have been identified in previous studies [18, 19, 26].

### Analyses of simple sequence repeats (SSRs) and long repeats

**Fig. 3:**
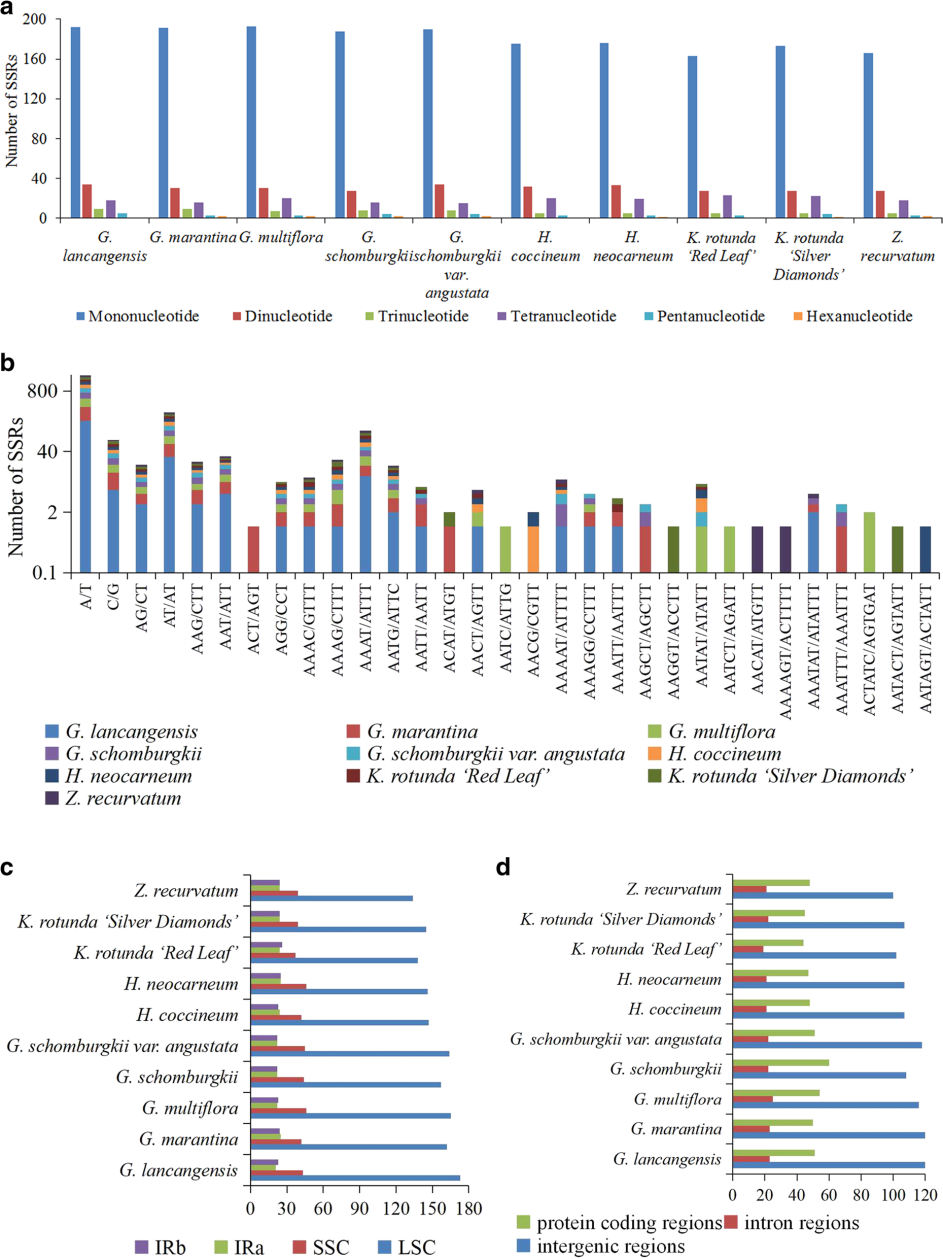
Comparison of the simple sequence repeats (SSRs) among ten sequenced chloroplast genomes in subfamily Zingiberoideae. **a** the number of different SSR types detected in ten Zingiberoideae chloroplast genomes. **b** the frequency of the identified SSRs in different repeat class types. **c** the frequencies of the identified SSRs in the LSC, SSC and IR regions. **d** the SSR distribution in protein coding regions, intron regions and intergenic regions detected in ten Zingiberoideae chloroplast genomes

In this study, there were 221 to 258 SSRs in each sequenced chloroplast genome that ranged from 8 to 27 bp in length (Fig. 3, Tables S7, S8). We discovered 6 types of SSRs, specifically, mononucleotide, dinucleotide, trinucleotide, tetranucleotide, pentanucleotide and hexanucleotide. Among these SSRs, only the chloroplast genomes of *G. marantina*, *G. multiflora*, *G. schomburgkii*, *G. schomburgkii var. angustata*, *H. neocarneum*, *K. rotunda* ‘Silver Diamonds’ and *Z. recurvatum* exhibited hexanucleotide repeats (Fig. 3a, Table S7). Among each sequenced chloroplast genome, mononucleotide repeats were the most frequent, with numbers ranging from 163 to 193, which accounted for 73.75–76.73% of all SSRs, followed by dinucleotide, ranging from 27 to 34 (11.02–13.92%), tetranucleotide, ranging from 15 to 23 (5.92–10.41%), trinucleotide, ranging from 5 to 9 (2.11–3.58%), pentanucleotide, ranging from 3 to 5 (1.17–1.93%), and hexanucleotide, ranging from 0 to 2 (0–0.90%) (Fig. 3a, Table S7). The majority of the mononucleotide SSRs were A/T repeats, which accounted for 94.79–97.59% of all the mononucleotide types among the ten sequenced chloroplast genomes (Fig. 3b, Table S7). In the dinucleotide repeats, the AT/AT repeats were observed most frequently, with 91.17–94.11% of the dinucleotide repeats (Fig. 3b, Table S7). In the tetranucleotide category, the AAAT/ATTT repeats were the most abundant type, with 48.14–66.67% of the loci in this category (Fig. 3b, Table S7). SSRs were more frequently located in the LSC regions (138–173 loci, 60.33–66.53%) than in the SSC regions (37–46 loci, 16.44–19.00%) and IR regions (44–50 loci, 17.39–22.22%) of the ten sequenced chloroplast genomes (Fig. 3c, Table S7). Likewise, SSRs were analyzed in the protein coding regions (exon, protein coding exon), intron regions and intergenic regions of the ten sequenced chloroplast genomes, which indicated that these ten sequenced chloroplast genomes contained 100 to 120 SSRs in intergenic regions, 19 to 25 SSRs in introns, and 44 to 60 SSRs in protein coding regions (Fig. 3d, Table S8).

**Fig. 4:**
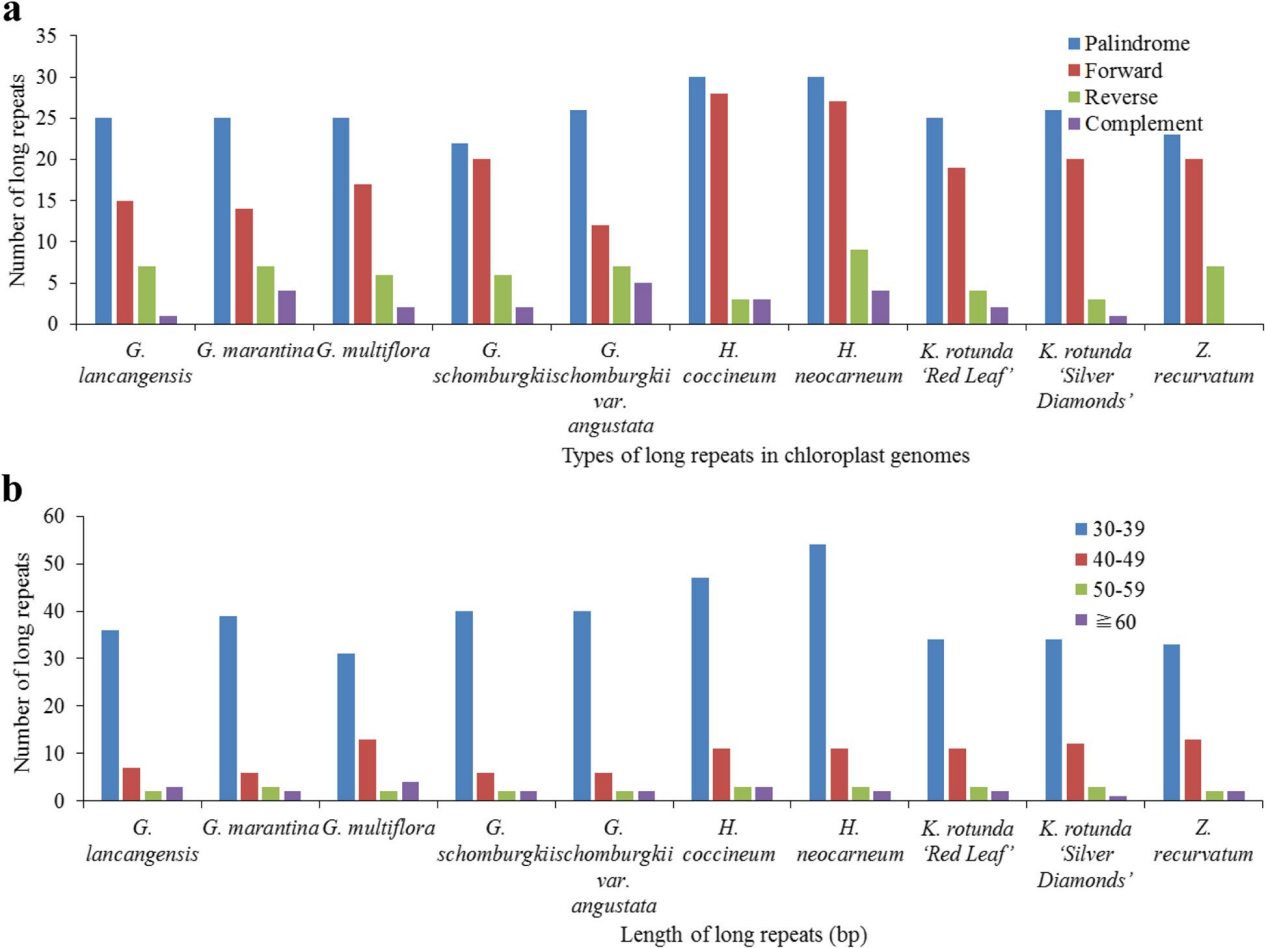
Long repeat sequences among ten sequenced chloroplast genomes in subfamily Zingiberoideae. **a** total of four long repeat types in ten Zingiberoideae chloroplast genomes and **b** numbers of long repeat sequences by length

Additionally, ten sequenced chloroplast genomes had 532 long repeats that consisted of 192 forward repeats, 24 complement repeats, 59 reverse repeats, and 257 palindromic repeats (Fig. 4a, Table S9). Among the ten sequenced genomes, *G. lancangensis* had the smallest number (48), and *H. neocarneum* had the largest number of long repeats (70) (Fig. 4a, Table S9). The number of forward repeats varied between 12 (*G. schomburgkii var. angustata*) and 28 (*H. coccineum*), the number of complement repeats varied from 1 (*G. lancangensis* and *K. rotunda* ‘Silver Diamonds’) to 5 (*G. schomburgkii var. angustata*), the number of reverse repeats varied between 3 (*H. coccineum* and *K. rotunda* ‘Silver Diamonds’) and 9 (*H. neocarneum*), and the number of palindromic repeats varied from 22 (*G. schomburgkii*) to 30 (*H. coccineum* and *H. neocarneum*) (Fig. 4a, Table S9). There was no complement repeat in *Z. recurvatum* (Fig. 4a, Table S9). Long repeats with 30–39 bp were found to be the most common in the ten sequenced chloroplast genomes (Fig. 4b, Table S9). Long repeats with a length of 30 bp were the most common (148), and those with lengths of 31 bp and 32 bp were the second (74) and third (63), most common, respectively (Table S9). Collectively, the number, length and distribution of these long repeats varied from one species to another among the nine tested species in the current study.

### Contraction and expansion of inverted repeats (IRs)

**Fig. 5:**
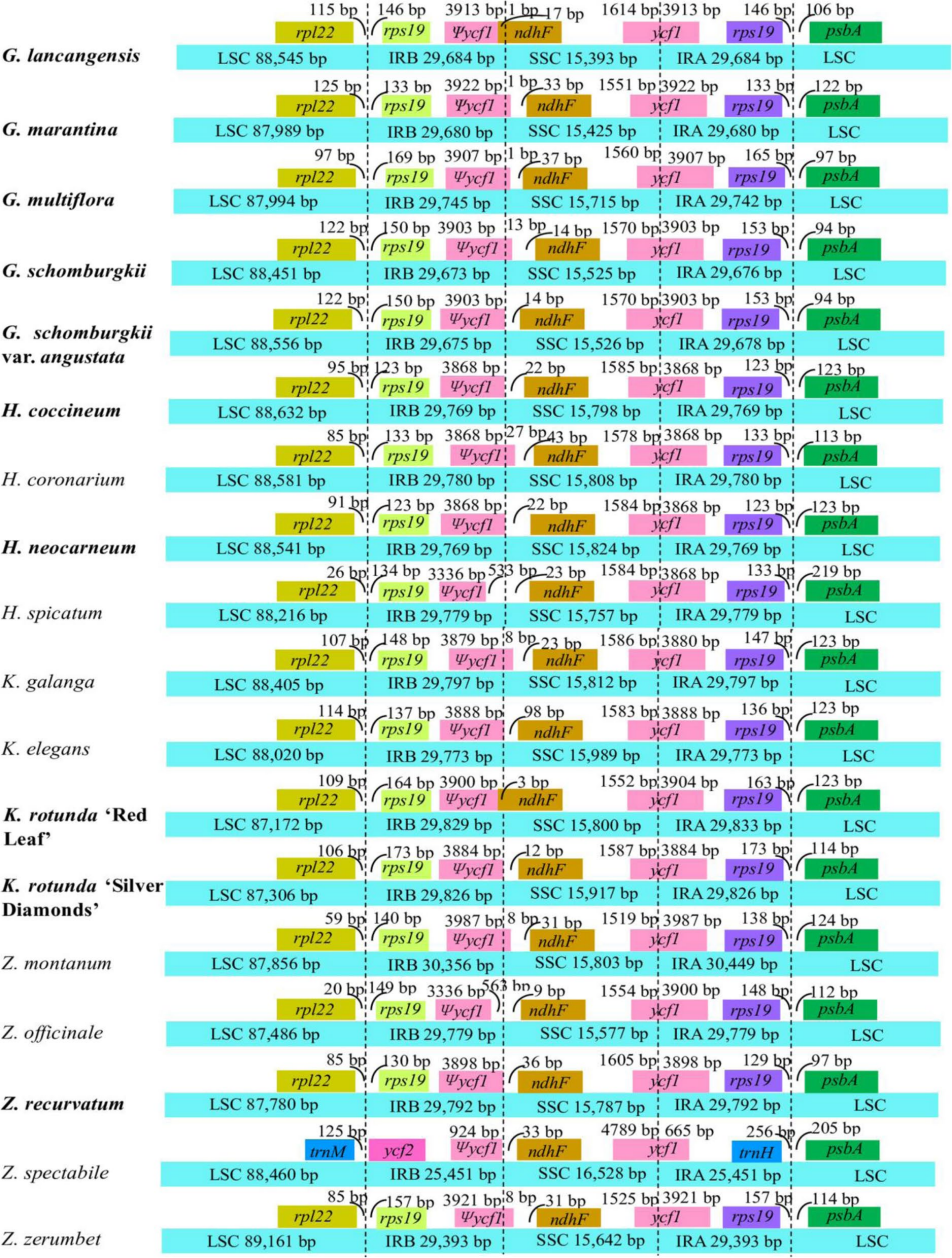
Comparisons of LSC, SSC and IR regions boundaries among 18 chloroplast genomes in subfamily Zingiberoideae. Ψ, pseudogenes. The figure was not to scale with respect to sequence length, and only showed relative changes at or near the IR/SC borders. The ten sequenced chloroplast genomes in this study were marked in bold

A comprehensive comparison at the LSC/IRs/SSC boundaries was performed among the 17 Zingiberoideae species, including 5 *Globba* species, 4 *Hedychium* species, 3 *Kaempferia* species and 5 *Zingiber* species (Fig. 5). Although the IR region of the 17 Zingiberoideae species’ chloroplast genomes was highly conserved, structure variation was still found in the IR/SC boundary regions. Within the 17 Zingiberoideae species, the *rpl22* and *rps19* genes were located in the boundaries of the LSC/IRB regions, except for *Z. spectabile*, in which there were *trnM* and *ycf2* genes, and the *rpl22/rps19* gene was absent in the boundaries of the LSC/IRB regions (Fig. 5). There were 20–125 bp between *rpl22* and the LSC/IRB borders within the rest of the 16 Zingiberoideae species, and the distance between *rps19* and the LSC/IRB boundary ranged from 123 bp to 173 bp (Fig. 5).

*Ψycf1*-*ndhF* genes were located at the boundaries of the IRB/SSC regions in all 17 Zingiberoideae species. The IRB/SSC borders of 8 species (*G. lancangensis*, *G. schomburgkii var. angustata*, *H. coccineum*, *H. neocarneum*, *K. elegans*, *K. rotunda* ‘Red Leaf’, *K. rotunda* ‘Silver Diamonds’, *Z. recurvatum* and *Z. spectabile*) were all situated adjacent to the end of *Ψycf1*, while 2 species (*H. spicatum* and *Z. officinale*) were found 533 bp and 563 bp distances between *Ψycf1* end and the IRB/SSC boundary, respectively. *Ψycf1* expanded into the SSC regions in 7 species, namely, *G. marantina*, *G. multiflora*, *G. schomburgkii*, *H. coronarium*, *K. galanga*, *Z. montanum* and *Z. zerumbet*, for 1 bp, 1 bp, 13 bp, 27 bp, 8 bp, 8 bp, and 8 bp, respectively (Fig. 5). There were 33 bp, 37 bp, 14 bp, 43 bp, 23 bp, 31 bp and 31 bp between the *ndhF* and *Ψycf1* border in *G. marantina*, *G. multiflora*, *G. schomburgkii*, *H. coronarium*, *K. galanga*, *Z. montanum* and *Z. zerumbet*, respectively (Fig. 5). In 9 species (*G. schomburgkii var. angustata*, *H. coccineum*, *H. neocarneum*, *H. spicatum*, *K. elegans*, *K. rotunda* ‘Silver Diamonds’, *Z. officinale*, *Z. recurvatum* and *Z. spectabile*), the distances between the *ndhF* and IRB/SSC border were 14 bp, 22 bp, 22 bp, 23 bp, 98 bp, 12 bp, 9 bp, 36 bp, and 33 bp, respectively. The *ndhF* gene was embedded in the IRB/SSC border and had a length of 1 bp in *G. lancangensis* and 3 bp in *K. rotunda* ‘Red Leaf’ (Fig. 5).

The SSC/IRA boundary was situated in the *ycf1* coding region, which crossed into the IRA region in all 17 Zingiberoideae species. However, the length of *ycf1* in the IRA region varied among the 17 Zingiberoideae species from 665 bp to 3987 bp, which indicated dynamic variation in the SSC/IRA boundaries (Fig. 5).

The *rps19* and *psbA* genes were situated in the boundaries of the IRA/LSC regions in all 17 Zingiberoideae species, except for *Z. spectabile*, in which the *trnH* gene was at one end of the IRA region 256 bp away from the IRA/LSC border; for the rest of the 16 Zingiberoideae species, the distances between *rps19* and the IRA/LSC border ranged from 123 bp to 173 bp (Fig. 5). For all 17 Zingiberoideae species, a 94–219 bp distance was observed between the *psbA* gene and the IRA/LSC border (Fig. 5).

### Comparative genomic and nucleotide diversity analyses

**Fig. 6:**
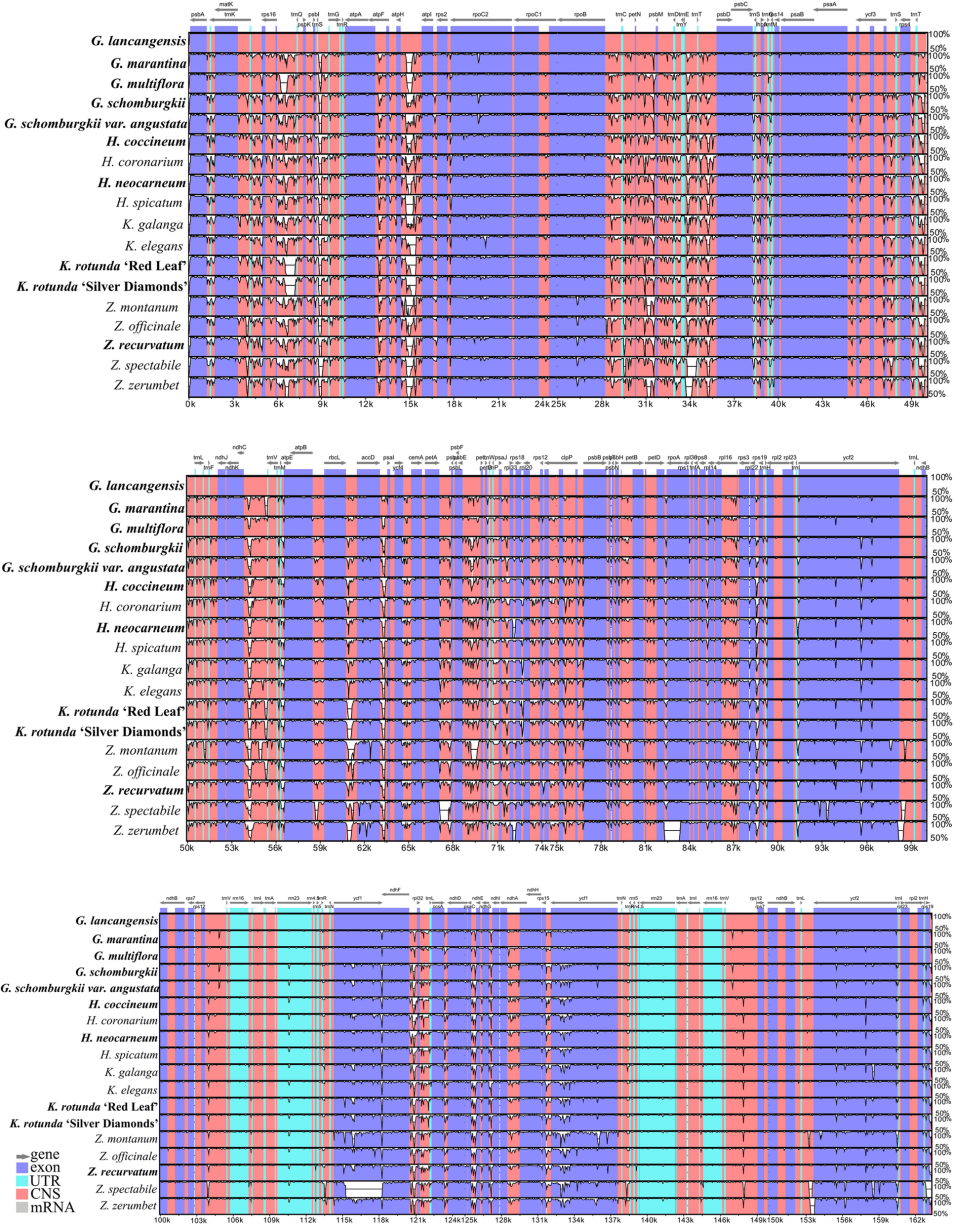
Comparative plots of percent sequence identity of 18 chloroplast genomes in subfamily Zingiberoideae. The chloroplast genome of *G. lancangensis* was used as a reference genome (upper plot). The percentage of sequence identities were visualized in mVISTA software [34]. Gray arrows and thick black lines indicated gene orientation. Purple bars represented exons, sky-blue bars represented untranslated regions (UTRs), red bars represented non-coding sequences (CNS), gray bars represented mRNA and white regions represented sequence differences among all analyzed chloroplast genomes. The horizontal axis indicated the coordinates within the chloroplast genome. The vertical scale represented the identity percentage that ranged from 50 to 100%. The ten sequenced chloroplast genomes in this study were marked in bold

Multiple alignments of 18 Zingiberoideae chloroplast genomes coming from 17 species of four genera, *Globba*, *Hedychium*, *Kaempferia* and *Zingiber*, were compared by using CGView and mVISTA, with the annotated *G. lancangensis* genome sequence as the reference (Figs. 1 and 6). The mVISTA comparison showed that the LSC and SSC regions were more divergent than the two IR regions and that a higher divergence was found in non-coding regions than in coding regions (Fig. 6). The main divergences for the coding regions were *accD*, *matK*, *psaJ*, *rpl32*, *rpl33*, *rps15*, *rps16* and *ycf1*. For the non-coding regions, strongly divergent regions were *psbA-trnK-UUU*, *rps16*-*trnQ-UUG*, *trnS-GCU*-*trnG-GCC*, *atpI-atpH*, *psbM-trnD-GUC*, *accD-psaI*, *ycf4*-*cemA*, *psaJ*-*rpl33*, *trnD-GUC-trnY-GUA*, *trnT-UGU*-*trnL-UAA*, *ndhF*-*rpl32*, *rpl32-trnL-UAG*, *ccsA-ndhD* and *rps15-ycf1* (Fig. 6). The CGView result also indicated that the two IR regions were less divergent than the LSC and SSC regions, and major differences originated from LSC and SSC regions (innermost fourth color ring to outwards 21st ring in Fig. 1). Compared to the chloroplast genome of *G. lancangensis* (innermost fourth color ring in Fig. 1), the other 17 chloroplast genomes shared five divergent regions in LSC (*trnS-GCU-trnG-GCC*, *atpI-atpH*, *trnT-GGU-psbD*, *psbM-trnD-GUC*, and *trnT-UGU-trnL-UAA*), one region in SSC (*ccsA-ndhD*) and one region in IRA (*ycf1*).

**Fig. 7:**
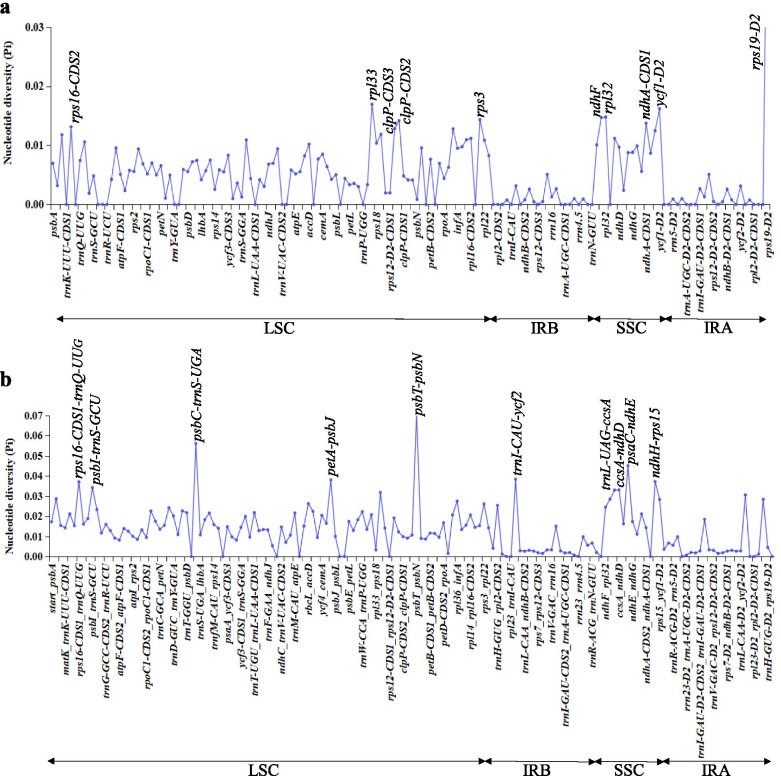
Nucleotide diversity (Pi) values of various regions in 18 chloroplast genomes in subfamily Zingiberoideae. **a** protein coding regions. Peak regions with a Pi value of > 0.0128 were labeled with loci tags of genic names. **b** intron and intergenic regions. Peak regions with a Pi value of > 0.033 were labeled with loci tags of intergenic region names

Furthermore, the nucleotide diversity (Pi) values were analyzed by DnaSP to test divergence level within different regions among the 18 Zingiberoideae chloroplast genomes from four genera, *Globba*, *Hedychium*, *Kaempferia* and *Zingiber* (Fig. 7). In the protein coding regions, the Pi values for each locus ranged from 0 to 0.00624 and had an average value of 0.00496 (Table S10a). Of these protein coding regions, ten regions (*rps16*-CDS2, *clpP*-CDS2, *clpP*-CDS3, *rps3*, *ndhF*, *rpl32*, *rpl33*, *ndhA*-CDS1, *ycf1*-D2 and *rps19*-D2) exhibited remarkably high values (Pi > 0.01313; Fig. 7a). For the intron and intergenic regions, the Pi values ranged from 0 to 0.07066 and had an average of 0.01327 (Table S10b). Among these intron and intergenic regions, ten most divergent regions of *rps16*-CDS1-*trnQ-UUG*, *psbI*-*trnS-GCU*, *psbC*-*trnS-UGA*, *petA*-*psbJ*, *psbT*-*psbN*, *trnI-CAU*-*ycf2*, *trnL-UAG*-*ccsA*, *ccsA*-*ndhD*, *psaC*-*ndhE* and *ndhH*-*rps15* with Pi values ranging from 0.03300 to 0.07066, were identified (Fig. 7b). Furthermore, using the region length ≧ 150 bp and Pi value ≥0.02018 for selection potential molecular markers, 23 regions were obtained: *accD-psaI*, *ccsA-ndhD*, *ndhF-rpl32*, *petA-psbJ*, *psaC-ndhE*, *psaJ-rpl33*, *psbA-trnK-UUU-CDS2*, *rbcL-accD*, *rpl32-trnL-UAG*, *rps18-rpl20*, *rps15-ycf1*, *rps16-CDS1-trnQ-UUG*, *psbM-trnD-GUC*, *trnD-GUC-trnY-GUA*, *trnE-UUC-trnT-GGU*, *trnG-UCC-trnfM-CAU*, *trnM-CAU-atpE*, *trnK-UUU-CDS1-rps16-CDS2*, *trnS-GCU-trnG-GCC-CDS1*, *trnT-UGU-trnL-UAA-CDS1*, *trnT-GGU-psbD*, *trnW-CCA-trnP-UGG* and *ycf4-cemA* (Table S10b).

Combing the results of mVISTA, CGView and DnaSP, 33 regions were extracted and constructed maximum likelihood (ML) trees to identify 17 Zingiberoideae species among four genera of *Globba*, *Hedychium*, *Kaempferia* and *Zingiber*. The resolution power of the divergent regions depended on the number of species successfully identified in ML trees. If the bootstrap value of the node was less than 50, species on the ML tree were not counted. Finally, based on the ML trees, 12 regions (*ndhF*, *ycf1*, *trnK-UUU-CDS1*-*rps16-CDS2*, *psaJ-rpl33*, *ycf4-cemA*, *trnT-UGU-trnL-UAA-CDS1*, *trnT-GGU-psbD*, *rpl32-trnL-UAG*, *psbM-trnD-GUC*, *ndhF-rpl32*, *rps15-ycf1* and *ccsA-ndhD*) showed relatively high resolution power at genus level. *ycf1* had the highest resolution power of 100%, followed by *trnK-UUU-CDS1*-*rps16-CDS2* at 88.89%, *ycf4-cemA* at 88.89%, *trnT-GGU-psbD* at 88.89%, *trnT-UGU-trnL-UAA-CDS1* at 88.89%, *ndhF* at 83.33%, *psaJ-rpl33* at 83.33%, and *rps15-ycf1* at 77.78% (Table 3, Fig. S2). Among these 8 regions, *ycf1* was shared by four genera of *Globba*, *Hedychium*, *Kaempferia* and *Zingiber*; *ndhF*, *trnK-UUU-CDS1*-*rps16-CDS2*, *psaJ-rpl33*, and *trnT-UGU-trnL-UAA-CDS1* were shared by *Globba*, *Kaempferia* and *Zingiber; trnT-GGU-psbD* was shared by *Hedychium*, *Kaempferia* and *Zingiber; rps15-ycf1* was shared by *Globba* and *Kaempferia*; and *ycf4-cemA* was shared by *Globba* and *Zingiber* (bootstrap values ≥50%) (Table 3). One DNA universal barcode, *matK,* exhibited resolution power of 77.78%. While four regions, *psbM-trnD-GUC*, *ndhF-rpl32*, *rpl32-trnL-UAG* and *ccsA*-*ndhD*, had the resolution power less than the resolution power of *matK*: *psbM-trnD-GUC* had the resolution power of 72.22%, *ndhF-rpl32* and *rpl32-trnL-UAG* had the same resolution power of 66.67%, and *ccsA*-*ndhD* had the lowest resolution power of 44.44% (Table 3). However, the three regions *rpl32-trnL-UAG, psbM-trnD-GUC*, and *ccsA*-*ndhD* could be used as candidate DNA barcodes for *Globba* species (all five species successfully differentiated with bootstrap values ≥71%); and *ndhF-rpl32* could be used as candidate DNA barcodes for *Globba* (bootstrap values ≥56%) and *Kaempferia* species (bootstrap values ≥87%), respectively (Table 3, Fig. S2i, j, m).

**Table 3:** Evaluation of the identification capability of thirteen regions among four genera in subfamily Zingiberoideae

Species	Bootstrap values of thirteen regions on ML trees												
	*matK*	*ndhF*	*ycf1*	*trnK-UUU-CDS1*-*rps16-CDS2*	*psaJ* *-rpl33*	*ycf4* *-cemA*	*trnT-UGU-trnL-UAA-CDS1*	*trnT-GGU-psbD*	*rpl32*-*trnL-UAG*	*psbM-trnD-GUC*	*ndhF* *-rpl32*	*rps15* *-ycf1*①	*ccsA* *-ndhD*
***G. lancangensis***	62	56	66	95	64	59	60	71	92	85	56	92	93
***G. marantina***	99	100	99	92	80	100	92	87	98	100	99	87	71
***G. multiflora***	99	56	100	99	98	82	99	71	92	99	56	98	71
***G. schomburgkii***	89	99	99	92	98	72	99	28	100	75	91	89	96
***G.schomburgkii*** **var. angustata**	89	99	99	92	98	72	99	28	100	75	91	89	96
***H. coccineum***	28	100	50	36	90	99	88	73	69	63	89	42	88
*H. coronarium*	100	27	100	36	20	26	100	100	99	100	100	26	20
***H. neocarneum***	28	24	82	71	17	70	30	54	29	28	31	26	14
*H. spicatum*	72	27	82	71	20	70	30	73	29	28	31	62	20
*K. galanga*	40	86	98	58	61	72	99	93	33	7	94	60	35
*K. elegans*	95	86	98	96	61	38	99	93	33	22	94	53	35
***K. rotunda*** **‘Red Leaf’**	96	100	100	99	97	95	87	99	82	97	87	93	62
***K. rotunda*** **‘Silver** **Diamonds’**	96	100	100	99	97	95	87	99	82	97	87	93	62
*Z. montanum*	63	55	56	87	84	72	84	98	54	63	56	61	35
*Z. officinale*	49	87	80	98	60	95	61	75	13	41	27	61	43
***Z. recurvatum***	85	94	100	93	94	93	100	94	91	81	44	47	21
*Z. spectabile*	63	98	56	86	60	72	81	64	13	89	20	53	35
*Z. zerumbet*	88	98	56	86	84	72	84	64	54	89	20	55	40
ratio(%)	77.78	83.33	100	88.89	83.33	88.89	88.89	88.89	66.67	72.22	66.67	77.78	44.44

Note: ratio (%) = [(the total number of species-the number of species with bootstrap values below 50%) /the total number of species] × 100%; ①: *ycf1* is here a protein coding gene in chloroplast genome. The sequenced species in this study were marked in bold

### Characterization of substitution rates and positive selection analyses

The nonsynonymous (Ka) and synonymous (Ks) nucleotide substitution rates of all 79 protein coding genes were analyzed across 17 Zingiberoideae species. Overall, the Ka/Ks ratios were less than 1.00 and invalid for most pairs comparison (95.37%) (Table S11a). There were 49 protein coding genes with Ka/Ks ratios greater than 1.00 and *p* values less than 0.05 at nucleotide level, such as *accD*, *psbJ*, *rbcL*, *rpl20*, *rps7*, *rps8*, *rps15*, *rps16*, *ycf1*, *ycf2* and so on (Table S11b). These data sets were so sophisticated and may generate false positives. To measure truly positive selection at the protein level for further, we used a Bayes empirical bayes (BEB) approach in PAML [35] to integrate over these uncertainties. The BEB method inferred that some amino acid sites of 14 protein coding genes were truly under positive selection with posterior probability greater than 0.95 (Table 4, Table S12). Among the 14 protein coding genes, *rps12* showed the highest number of positive amino acids sites (40), followed by *ycf1* (34) and *ycf2* (20) (Table 4). The other 11 protein coding genes, *accD*, *ccsA*, *ndhA*, *ndhB*, *psbJ*, *rbcL*, *rpl20*, *rpoC1*, *rpoC2*, *rps18*, and *ycf4*, presented 4, 4, 5, 3, 1, 8, 2, 2, 2, 1, and 2 amino acids sites truly under positive selection, respectively (Table 4). The amino acids encoded by the sites *rpl20* (118), *ycf1* (1341, 1433, 1452, 1453, 1528 and 1586), and *ycf2* (1343) were exclusively found in *G. lancangensis* among all 17 Zingiberoideae species studied here (Table S12). Additionally, some amino acids positions were highly variable, such as *accD* (4, 9 and 299), *ccsA* (180), *rbcL* (449), *rps18* (27) and *ycf1* (215, 928 and 1452), which displayed three or more amino acid changes among all 17 Zingiberoideae species studied here (Table S12).

**Table 4:** Positive selective amino acid loci and estimation of parameters for fourteen genes in subfamily Zingiberoideae

Gene	Ln L	Estimates of parameters	Positively selected sites
*accD*	−2726.384456	p0 = 0.96351 *p* = 0.00502 q = 0.00512 (p1 = 0.003649) ω = 11.82130	4 W 1.000**, 9 L 0.987*, 218H 0.958*, 299R 0.968*
*ccsA*	− 1712.097311	p0 = 0.97744 *p* = 0.00507 q = 0.02055 (p1 = 0.02256) ω = 13.95433	87 T 0.957*, 180 L 0.994**, 200Y 1.000**, 201 K 1.000**
*ndhA*	− 1991.585023	p0 = 0.98899 *p* = 0.01051 q = 0.03386 (p1 = 0.01101) ω = 57.72615	132F 0.964*, 189S 1.000**, 190S 1.000**, 191 T 1.000**, 192 V 1.000**
*ndhB*	− 2119.544926	p0 = 0.98391 *p* = 0.00500 q = 13.07861 (p1 = 0.01609) ω = 43.84612	133 V 0.953*, 181 T 0.957*, 246P 0.955*
*psbJ*	−162.569004	p0 = 0.97242 *p* = 0.00500 q = 18.09039 (p1 = 0.02758) ω = 999.00000	20 L 1.000**
*rbcL*	− 2346.044248	p0 = 0.97658 *p* = 0.01215 q = 0.20915 (p1 = 0.02342) ω = 14.08875	169 L 0.980*, 225I 0.996**, 226Y 0.997**, 247C 0.963*, 255I 0.955*, 407 L 0.980*, 424 L 0.999**, 449S 1.000**
*rpl20*	−806.763954	p0 = 0.94834 *p* = 0.00500 q = 0.01576 (p1 = 0.05166) ω = 8.41059	118 K 0.998**, 125Y 1.000**
*rpoC1*	− 3213.853612	p0 = 0.98613 *p* = 33.03585 q = 99.00000 (p1 = 0.01387) ω = 11.40945	147 N 0.972*, 606D 0.971*
*rpoC2*	− 6806.754011	p0 = 0.98887 *p* = 0.04146 q = 0.09596 (p1 = 0.01113) ω = 11.89405	711Y 0.995**, 1174 W 0.984*
*rps12*	− 759.324938	p0 = 0.73184 *p* = 17.58063 q = 0.00500 (p1 = 0.26816) ω = 772.95793	1 M 0.955*, 2P 0.996**, 3 T 0.961*, 4I 0.956*, 5 K 0.956*, 6Q 1.000**, 7 L 0.998**, 8I 0.999**, 9R 0.974*, 10 N 0.999**, 11A 0.998**, 12R 0.959*, 13Q 1.000**, 14P 0.966*, 15I 0.989*, 16R 0.959*, 17 N 0.999**, 18 V 0.999**, 19 T 1.000**, 20 K 1.000**, 21S 0.998**, 22P 0.998**, 23A 0.963*, 24 L 0.998**, 25R 0.986*, 26E 0.998**, 27C 0.964*, 28P 0.998**, 29Q 1.000**, 30R 0.998**, 31R 0.999**, 32G 0.999**, 33 T 0.999**, 34C 0.962*, 35 T 0.956*, 36R 0.958*, 37 V 0.998**, 38Y 0.960*, 94R 0.952*, 116Q 0.952*
*rps18*	− 553.014018	p0 = 0.98952 *p* = 55.09674 q = 99.00000 (p1 = 0.01048) ω = 49.80264	27P 0.973*
*ycf1*	−10,584.294185	p0 = 0.88238 *p* = 40.44610 q = 56.04336 (p1 = 0.11762) ω = 7.72414	14 L 0.994**, 16 M 0.985*, 48R 0.961*, 142 L 0.990**, 212A 0.989*, 215R 0.981*, 606D 0.975*, 663R 0.994**, 809Y 0.952*, 928P 0.992**, 1293 V 0.986*, 1302I 0.964*, 1341 M 1.000**, 1433 K 0.992**, 1439 N 0.999**, 1452 K 0.984*, 1453 K 0.982*, 1466 K 0.999**, 1469S 0.998**, 1473D 0.999**, 1499D 0.966*, 1506Q 0.991**, 1528E 0.988*, 1576F 0.990**, 1586Y 1.000**, 1590 K 0.990**, 1604P 0.990**, 1621A 0.987*, 1628 L 0.991**, 1629 N 0.993**, 1632D 0.993**, 1651G 0.987*, 1667S 0.995**, 1757 L 0.961*
*ycf2*	−10,373.971098	p0 = 0.93261 *p* = 0.10353 q = 0.15548 (p1 = 0.06739) ω = 20.73253	220P 0.993**, 998D 0.993**, 1069I 0.993**, 1324 L 0.994**, 1343F 1.000**, 1411S 0.993**, 1665I 0.993**, 1758R 0.993**, 1977A 0.993**, 2121D 0.999**, 2191R 0.994**, 2261 L 0.963*, 2263H 0.993**, 2265 T 0.999**, 2266G 0.995**, 2267E 0.993**, 2268R 0.993**, 2269F 0.999**, 2271I 0.993**, 2272P 0.994**
*ycf4*	−960.017298	p0 = 0.91059 *p* = 0.00500 q = 1.92107 (p1 = 0.08941) ω = 4.34827	181 M 0.962*, 184 L 0.971*

Note: the degree of freedom for each gene was 38; * and ** indicate posterior probability higher than 0.95 and 0.99, respectively

### Phylogenetic inference of subfamily Zingiberoideae

**Fig. 8:**
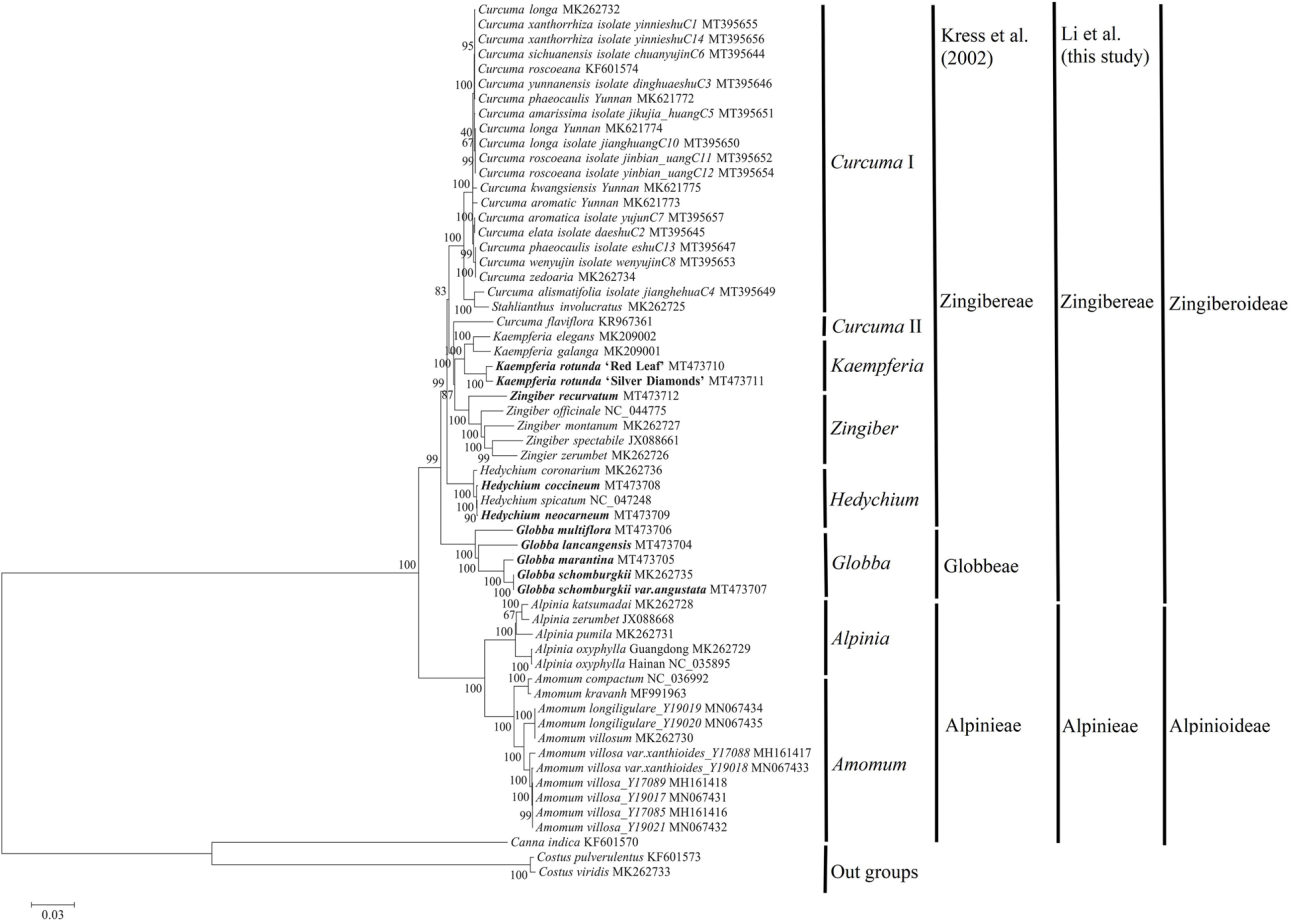
Molecular phylogenetic tree based on the SNPs from 56 chloroplast genomes of family Zingiberaceae. *C. indica*, *C. pulverulentus* and *C. viridis* set as the outgroups. The tree was constructed with maximum likelihood analysis of SNP matrix using MEGA software [32]. The stability of each tree node was tested by bootstrap method with 1000 replicates. Bootstrap values ≧ 50% were indicated numbers next to the branches. The ten sequenced chloroplast genomes in this study were marked in bold

To examine the phylogenetic positions of 5 *Globba* species, 4 *Hedychium* species, 3 *Kaempferia* species and 5 *Zingiber* species, and their relationships within subfamily Zingiberoideae, Bayesian inference (BI) and ML trees were constructed based on the single nucleotide polymorphism (SNP) matrix from 59 chloroplast genomes (Fig. 8, Fig. S3). These chloroplast genomes included 16 of subfamily Alpinioideae, 40 of subfamily Zingiberoideae, and 3 species (*Canna indica*, *Costus pulverulentus*, and *Costus viridis*) as the outgroups. The topological structures of the BI and ML trees were consistent, and were divided into two subfamilies of Alpinioideae and Zingiberoideae with strong support (posterior probability = 1.00 for the BI tree and bootstrap value = 100% for the ML tree) (Fig. 8, Fig. S3). In the BI tree, the posterior probabilities of all nodes reached 1.00 (Fig. S3), which indicated that all nodes were strongly supported. In the ML tree, there were two genera (*Amomum* and *Alpinia*) in subfamily Alpinioideae, and six genera (*Curcuma*, *Globba*, *Hedychium*, *Kaempferia*, *Stahlianthus*, and *Zingiber*) in subfamily Zingiberoideae with moderate to strong support (bootstrap values = 83–100%) (Fig. 8).

Within subfamily Zingiberoideae, there were two clusters *Curcuma* I and *Curcuma* II in genus *Curcuma*: *Curcuma* II comprised only one species (*C. flaviflora*), while the rest of the *Curcuma* species were grouped in *Curcuma* I; *Curcuma* I also included genus *Stahlianthus*; for genus *Globba*, *G. schomburgkii* was first grouped with *G. schomburgkii var. angustata* with strong support (bootstrap value = 100%), and had a nested relationship with *G. marantina*, *G. lancangensis*, and *G. multiflora* (bootstrap values = 100%); for genus *Hedychium, H. neocarneum* and *H. spicatum* were first clustered together with strong support (bootstrap value = 90%), and then clustered with *H. coccineum* and *H. coronarium* with strong support (bootstrap values = 100%); for genus *Kaempferia*, there were two clusters with strong support (all bootstrap values = 100%), one of which included two forms of *K. rotunda* (*K. rotunda* ‘Red Leaf’ and *K. rotunda* ‘Silver Diamonds’), and the other included *K. galanga* and *K. elegans*; for genus *Zingiber*, *Z. zerumbet*, *Z. spectabile*, *Z. montanum*, *Z. officinale* and *Z. recurvatum* were clustered step-by-step with strong support (bootstrap values ≥99%) (Fig. 8). Meanwhile, four genera *Globba, Hedychium*, *Kaempferia* and *Zingiber* were monophyletic in the ML tree; *Kaempferia* and *Zingiber* were strongly supported as sister genera (bootstrap value = 87%), and *Curcuma* II was the other sister for *Kaempferia* with high support (bootstrap value = 100%); *Curcuma* I and *“*the *Kaempferia* clade” consisting of *Curcuma* II, *Kaempferia* and *Zingiber*, were moderately supported as sisters (bootstrap value = 83%). Interestingly, *Curcuma* I + *“*the *Kaempferia* clade”, *Hedychium* and *Globba* had a nested evolutionary relationship with strong support (bootstrap values = 99%) in the ML tree (Fig. 8). In conclusion, the BI and ML phylogenetic trees showed clear relationships among the four genera of *Globba*, *Hedychium*, *Kaempferia* and *Zingiber* in subfamily Zingiberoideae.

## Discussion

### Chloroplast genome structure and sequence variation

In this study, 10 complete chloroplast genomes of 9 species from four genera of subfamily Zingiberoideae, namely, *Globba*, *Hedychium*, *Kaempferia* and *Zingiber*, were sequenced, assembled and applied for their comparative analyses with other related Zingiberoideae species [15, 21, 23, 26]. The genome size (between 162,630 bp and 163,968 bp), genome quadripartite structure (one LSC, one SSC and two IR regions), GC content (ranging from 35.73 to 36.18%), gene composition, most of the protein coding genes, tRNAs and rRNAs showed high similarities among the 10 sequenced chloroplast genomes, which had been observed in other Zingiberoideae chloroplast genomes [15, 21–23, 26]. Although the chloroplast genomes of Zingiberoideae species were highly conserved, gene loss, intron loss and gene duplication occurred in current study, for example, both *H. coccineum* and *H. neocarneum* lost *lhbA* gene, suggesting that gene loss and insertion had occurred during the evolutionary process of *H. coccineum* and *H. neocarneum*. It is important to mention that the chloroplast genome of *G. schomburgkii* lost both *trnS-GGA* and *trnT-GGU*, but had two copies of both *trnS-GCU* and *trnT-UGU*. Additionally, the *trnG-GCC* contained one intron in chloroplast genomes of *G. lancangensis* and *G. schomburgkii*, while the remaining sequenced genomes lost this intron. In comparison to other angiosperms chloroplast genomes, there have been many reports of gene loss of *accD*, *ndh* genes, *psbE, rpl2*, *rpl23*, *trnL*-*CCA*, *trnG*-*GCC*, and *ycf15*, as well as intron loss and gene duplication of *rpl2*, *rpl23*, *rps15* and *ycf1* [36–42].

Contraction and expansion at the borders of the IR regions of chloroplast genomes are considered to be important evolutionary events and may cause size variations, the origination of pseudogenes, gene duplication or the reduction of duplicate genes to single copies [41–44]. For instance, the IR region in *Heimia myrtifolia* is 25,643 bp long, which is shorter than the IR region of most *Lagerstroemia* species, indicating that the *Lagerstroemia* species differentiated later than *H. myrtifolia* in family Lythraceae [41]. After comparing the chloroplast genomes among 17 Zingiberoideae species, we found that the boundaries between the SSC and two IR regions were relatively conserved, and the distribution and locations of gene types in these regions were highly consistent. Compared with the other 16 Zingiberoideae species, the length of IR region in *Z. spectabile* was the smallest (25,451 bp), mainly because the *ycf2* gene located at the LSC/IRB boundary. Additionally, among studied 17 Zingiberoideae species, only two tRNAs of *Z. spectabile* were found at the LSC/IRB and LSC/IRA boundaries, respectively. Therefore, changes in the LSC/IR boundaries may be the main contributors to the contraction and expansion of IR regions in these Zingiberoideae species.

Highly variable regions can be used as potential DNA barcode markers for the studies on phylogenetic relationships, species identification and population genetics [37, 41, 42, 45]. However, for some *Globba*, DNA barcode markers have been considered to be difficult to identify. For example, the DNA barcode markers, *ITS*, *matK* and *trnK-matK*, could not discriminate *G. atrosanguinea* and *G. crutisii* [2], and *G. fecunda* and *G. multifolia* [10]. Meanwhile, based on the Pi values studied here, it is also obvious that the frequently used chloroplast markers, including *matK*, *trnK-matK* and *trnL-trnF*, present low polymorphisms (0.0117, 0.0154, 0.0134, respectively) at subfamily level. Therefore, it is important to explore more highly variable regions at subfamily level that represent potential markers, which can be used for future studies. Based on the results of mVISTA, CGView, nucleotide diversity and ML trees, 12 divergent regions among 17 Zingiberoideae species are suitable for species identification at subfamily and genus level. By comparison, we find that 5 of them are also reported in some Zingiberaceae species, such as *ccsA*-*ndhD*, *ndhF*-*rpl32*, *ycf1* and *ycf4-cemA* reported in *Alpinia* [13, 19], *ycf1* reported in *Curcuma* [18], *ndhF*-*rpl32* reported in *Kaempferia* [21], and *trnT-UGU-trnL-UAA* and *ycf1* reported in *Zingiber* [15, 26]. Our results further verify the reliability and effectiveness of these 5 divergent regions. In addition, among 9 divergent regions, *ndhF*, *ndhF-rpl32*, *psbM*-*trnD-GUC*, *rpl32*-*trnL-UAG* and *trnK-UUU*-*rps16* are also reported in Lythraceae [45], *ndhF* and *ycf1* are also reported in Monsteroideae [46], *rpl32*-*trnL-UAG*, *rps15-ycf1*, *trnK-UUU*-*rps16*, and *trnT-GGU-psbD* are also reported in *Spathiphyllum* [46], *rpl32-trnL-UAG* is also reported in *Euterpe* [47], *rps15-ycf1* is also reported in *Prunus* [48], and *trnK*-*rps16* (exon2-intron), *trnT-trnL* and *ycf1* are also reported in *Allium* [49]. Based on these results, we suggest that these divergent regions can be used for potential marker resources of subfamily Zingiberoideae in studies of species identification and phylogeny.

### Chloroplast genome evolution in subfamily Zingiberoideae

To resolve the evolutionary history of Zingiberoideae species, it is necessary to analyze their adaptive evolution. The Ka/Ks ratio is very useful for measuring selection pressure at the protein level: if Ka/Ks > 1, the protein is considered to be positively selected; if Ka/Ks = l, the protein is neutral; and if Ka/Ks < 1, the protein is considered to have undergone purifying selection [50, 51]. In this study, 14 genes with positive selection sites are identified in four genera of *Globba*, *Hedychium*, *Kaempferia* and *Zingiber* in subfamily Zingiberoideae. Among these genes containing amino acid positive sites, three genes of them encoding ribosome subunit proteins (*rpl20*, *rps12* and *rps18*), are involved in chloroplast gene expression, which is essential for chloroplast biogenesis and function [52]. *rps12* gene exhibits its variations in intragenic exon location and intron content, and has important effects on evolutionary rates and patterns of molecular evolution in fern [53]. Its spicing activity has been reported to impair photosynthesis and perturb development in *Arabidopsis* [52]. In our analyses, *rps12* gene harbors the highest number (40) of positive amino acid sites within 17 Zingiberoideae species, suggesting that the *rps12* gene may play important roles in Zingiberoideae species evolution and development.

Moreover, ten genes have also been identified with positive selection sites in current study, namely, *accD*, *ccsA*, *ndhB*, *psbJ*, *rbcL*, *rpoC1*, *rpoC2*, *ycf1*, *ycf2* and *ycf4*. Recent studies have indicated that these ten genes with positive selection in some angiosperms may be very common phenomena [14, 26, 37, 45–47, 50, 51, 54]. For examples, *accD*, *ndhB*, *ycf1* and *ycf2* have been reported as positive selection in some Zingiberaceae species [14, 26]; *accD*, *ccsA*, *rbcL*, *rpoC1*, *rpoC2*, *ycf1*, *ycf2* and *ycf4* have also been identified under positive selection in Orchidaceae [37]; *accD*, *ccsA*, *psbJ*, *rbcL*, *ycf1* and *ycf4* have also been identified under positive selection in Lythraceae [45, 50]; *accD*, *ccsA*, *rbcL*, *rpoC2*, *ycf1* and *ycf2* have also been identified under positive selection in *Euterpe* [47]; *accD*, *rbcL* and *ycf2*, have also been identified under positive selection in Monsteroideae [46]; *accD*, *ndhB* and *ycf2* have also been identified under positive selection in *Pterocarpus* [51]; and *ycf2* has also been identified under positive selection in *Pyrus* [54]. Additionally, among these ten genes, we find that *rbcL*, *ycf1* and *ycf2* genes possess higher number (8, 34, 20, respectively) of positive amino acid sites within Zingiberoideae species. *rbcL* gene encodes large subunit of ribulose-1,5-bisphosphate carboxylase/oxygenase (rubisco), and *ycf1* and *ycf2* genes encode unknown function proteins. Both *rbcL* and *ycf1* genes are intensively used for species identification, phylogenetic, phylogeography, germplasm conservation and innovative utilization of many plants [55–58]. Lastly, there is one gene (*ndhA*) encoding subunit of NADH-plastoquinone oxidoreductase, which is found under positive selection with 5 amino acid sites. Among our analyzed species of *Globba*, *Hedychium*, *Kaempferia* and *Zingiber* here, they own diverse pseudostem heights and habitats; for instance, *K. galanga* spreads flat on ground, living in open areas, while *G. schomburgkii*, *H. coronarium*, and *Z. officinale* have pseudostems heights of 30–50 cm, 1–3 m, and 50–100 cm, respectively, native to forests [1, 3–5]. In other words, they live in diverse environment in their respective habitats, such as temperature, light and humidity, and keep high levels of plant diversity. We accept that our analyzed plants do not fully contain all of Zingiberoideae plants, and that they may exist genetic variations by themselves. Nonetheless, based on our results, we propose that positive selection and environmental heterogeneity may interconnect together to contribute to Zingiberoideae species evolution and adaption.

### Phylogenetic analyses in subfamily Zingiberoideae

Over the past two decades, phylogenetic relationships within four genera of *Globba*, *Hedychium*, *Kaempferia* and *Zingiber* in subfamily Zingiberoideae, had been some ambiguous in previous phylogenies [2, 8–11], for examples, regarding *Globba*, nuclear *ITS* and chloroplast *matK* data had very low resolution or were generally lacking among *Globba* species [10]; and for *Hedychium*, nuclear *ITS* and chloroplast *matK/trnL-trnF* molecular data had strongly supported the monophyly of *Hedychium*, but its relationships with other genera had been poorly supported [2, 8, 9]. In this study, both BI and ML phylogenetic trees have demonstrated some congruence with previous phylogenies in subfamily Zingiberoideae, for instance, the monophyly of *Hedychium* [2, 8, 9]. Of course, our analyses also have strongly identified that *Globba*, *Hedychium*, and *Curcuma* I + *“*the *Kaempferia* clade” consisting of *Curcuma* II, *Kaempferia* and *Zingiber*, display a nested evolutionary relationship with high resolution in subfamily Zingiberoideae (Fig. 8, Fig. S3).

Regarding *Globba*, *Globba* was classified into tribe Globbeae, which was one of the two tribes of subfamily Zingiberoideae in previous phylogenies [2, 10]. In our analyses, the close evolutionary relationship between *Globba* and *Hedychium* as well as their genetic boundaries have been identified (Fig. 8, Fig. S3). Therefore, the taxonomic position of *Globba* requires some discussion. On the one hand, *Globba* owns the universal morphological characters of tribe Zingibereae, which has the parallel orientation of the plane of the distichy of the leafy shoots with respect to the rhizome, and the conspicuous and often well-developed lateral staminodes [2, 3, 10]. On the other hand, *Hedychium* is classified into tribe Zingibereae, and *Globba* is close to *Hedychium* with high resolution based on current phylogenetic results (Fig. 8, Fig. S3). We confirm that our results do not completely resolve all of relationships among genera in two tribes of subfamily Zingiberoideae, and that our results do not sample a great deal of *Globba* species. In spite of all that, based on the results of our molecular phylogenies, we suggest a realignment of the tribe of *Globba* in subfamily Zingiberoideae: *Globba* is here transferred into tribe Zingibereae (Fig. 8, Fig. S3). Because of two important genera, *Gagnepainia* and *Hemiorchis*, were classified into tribe Globbeae in past phylogenies [2, 10], we recommend that retaining the tribe Globbeae as previous recognized until future new evidence proves otherwise.

## Conclusions

In this study, ten complete chloroplast genomes from nine Zingiberoideae species, namely, *G. lancangensis*, *G. marantina*, *G. multiflora*, *G. schomburgkii*, *G. schomburgkii var. angustata*, *H. coccineum*, *H. neocarneum*, *K. rotunda* ‘Red Leaf’, *K. rotunda* ‘Silver Diamonds’ and *Z. recurvatum*, have been sequenced, assembled and annotated for the first time. The structural characteristics of these ten chloroplast genomes are shown to be conservative, which are similar to those reported chloroplast genomes of Zingiberoideae species. Meanwhile, comparative analyses of 18 Zingiberoideae chloroplast genomes have generated 12 highly variable regions, which may be used as a potential source of molecular markers for phylogenetic analysis and species identification. Based on whole chloroplast-derived SNP data, phylogenetic relationships among four genera of *Globba*, *Hedychium*, *Kaempferia* and *Zingiber* in subfamily Zingiberoideae have been clearly resolved. In addition, at level of amino acid sites, 14 genes are under positive selection with high posterior probabilities, which may play important roles in Zingiberoideae species evolution and adaption to diverse environment. These results increase the genomic resources available for subfamily Zingiberoideae, which will be useful for future studies of evolution and phylogenetic.

## Methods

### Plant material sampling and chloroplast DNA extraction

We generated data on ten chloroplast genomes for nine species within the Zingiberoideae subfamily. Fresh and healthy leaves (*G. lancangensis*, *G. marantina*, *G. multiflora*, *G. schomburgkii*, *G. schomburgkii var. angustata*, *H. coccineum*, *H. neocarneum*, two forms of *K. rotunda*, namely *K. rotunda* ‘Red Leaf’ and *K. rotunda* ‘Silver Diamonds’, and *Z. recurvatum*) were collected from the resource garden of the environmental horticulture research institute (23°23′N, 113°26′E) at the Guangdong Academy of Agricultural Sciences, Guangzhou, China, and were immediately frozen in liquid nitrogen and stored at − 80 °C. The total genomic DNA was extracted from these leaves using the improved sucrose gradient centrifugation method [59]. The concentration and quantity of each isolated genomic DNA sample were determined with a NanoDrop 2000 micro spectrometer (Wilmington, DE, USA) and 1% agarose gel electrophoresis, respectively.

### Chloroplast genome sequencing, assembly and annotation

For *G. schomburgkii*, two libraries with insert sizes of 300 bp and 10 kb were constructed after purification and then sequenced on an Illumina Hiseq X Ten instrument (Biozeron, Shanghai, China) and a PacBio Sequel platform (Biozeron, Shanghai, China), respectively. For the other species (*G. lancangensis*, *G. marantina*, *G. multiflora*, *G. schomburgkii var. angustata*, *H. coccineum*, *H. neocarneum*, *K. rotunda* ‘Red Leaf’, *K. rotunda* ‘Silver Diamonds’, and *Z. recurvatum*), a library with insert sizes of 300 bp was constructed after purification for each species and then sequenced on an Illumina Hiseq X Ten instrument (Biozeron, Shanghai, China).

The raw data were assessed using FastQC (http://www.bioinformatics.babraham.ac.uk/projects/fastqc/), and the adaptor sequences were removed by Trimmomatic v.0.3 [60]. For the final assembled chloroplast genomic sequence of *G. schomburgkii*, the Illumina paired-end clean reads and PacBio clean data were assembled using the previously described method [19, 21, 26], with the software SOAPdenovo v.2.04 [61], Geneious v.11.0.4 [62], BLASR [63], Celera Assembler v.8.0 [64] and GapCloser v.1.12 in SOAPdenovo v.2.04 [61]. The other species’ Illumina clean sequences were assembled into complete chloroplast genomes with SOAPdenovo v.2.04 with default parameters [61] using the chloroplast genome of *G. schomburgkii* as the reference (Table S1).

Complete chloroplast genomes were annotated using the online tool DOGMA (Dual Organellar Genome Annotator) [65] with default parameters, and then, it was checked manually. tRNAs and rRNAs were annotated by BLASTn searches on the NCBI website. A verification of tRNAs and rRNAs was performed using tRNAscanSE with default parameters [66] (Tables 1, 2; Tables S2, S3). Finally, the OGDRAW v.1.3.1 program was used with default settings to draw the circular chloroplast genome maps of the Zingiberoideae species and was manually edited [30] (Fig. 1; Fig. S1).

### Prediction of codon usage and RNA editing sites

To examine the deviation in synonymous codon usage, the relative synonymous codon usage (RSCU) was calculated using MEGA v.7 [32] (Fig. 2a, Table S4). When the RSCU value > 1.00, this means that the use of a codon is more frequent than expected, and vice versa [39, 43]. The clustered heat map of RSCU values of ten sequenced Zingiberoideae chloroplast genomes was conducted by R v.3.6.3 [33] (Fig. 2b, Table S5). To predict possible RNA editing sites in the ten sequenced chloroplast genomes, protein coding genes were used to predict potential RNA editing sites using the online program Predictive RNA Editor for Plants (PREP) suite (http://prep.unl.edu/) with a cutoff value of 0.8 [67] (Table S6).

### Analyses of SSRs and long repeats

MIcroSAtellite (MISA) (http://pgrc.ipk-gatersleben.de/misa/) was used to detect the location and number of SSRs of the ten sequenced chloroplast genomes with the settings as follows: ≥ 8 for mono-, ≥ 5 for di-, ≥ 4 for tri-, and ≥ 3 for tetra-, pena-, and hexa-nucleotide SSRs (Fig. 3, Tables S7, S8). The REPuter software was employed to identify long repeats such as forward, palindrome, reverse and complement repeats [68]. The criteria for determining long repeats were as follows: (1) a minimal repeat size of more than 30 bp; (2) a repeat identity of more than 90%; and (3) a hamming distance equal to 3 (Fig. 4, Table S9).

### Comparative genomic analysis

To detect the contractions and expansions of the IRs in the chloroplast genomes of the four genera (*Globba*, *Hedychium*, *Kaempferia* and *Zingiber*), 18 whole genomes within subfamily Zingiberoideae were compared (Fig. 5). Using the annotated chloroplast genome of *G. lancangensis* as the reference, the mVISTA tool with the Shuffle-LAGAN mode [34] was used to make pairwise alignments among these 18 whole chloroplast genomes (Fig. 6). Additionally, the *G. lancangensis* chloroplast genome was compared to 17 chloroplast genomes of the four genera (*Globba*, *Hedychium*, *Kaempferia* and *Zingiber*) using CGView [31] (Fig. 1). GC distributions were measured based on GC skew using the following equation: GC skew = (G-C)/(G + C). The nucleotide variability (Pi) of the protein coding, intron and intergenic regions among these 18 chloroplast genomes within the Zingiberoideae subfamily was extracted and then calculated using DnaSP v.6 [69] (Fig. 7, Table S10). To identify the highly divergent regions, the protein coding regions and intergenic regions were extracted and then manually aligned using ClustalW in MEGA v.7 [32]. To obtain the molecular markers for differentiating the four genera in subfamily Zingiberoideae, a maximum likelihood (ML) analysis based on the nucleotide substitution model of Tamura-Nei was conducted using MEGA v.7 with 1000 replicates [32] (Table 3, Fig. S2).

### Characterization of substitution rates and positive selection analyses

To calculate the nonsynonymous (Ka) and synonymous (Ks) substitution rates, the 79 protein coding genes of 18 Zingiberoideae chloroplast genomes, coming from four genera of *Globba*, *Hedychium*, *Kaempferia* and *Zingiber*, were extracted from their genomes, respectively. Then, the KaKs_Calculator with default parameters was used to calculate the rates of Ka, Ks and their ratio (Ka/Ks, denoted ω) for each gene of 18 Zingiberoideae chloroplast genomes by comparing pairwise [70]. A total of 11,835 Ka/Ks were obtained; the value could not be calculated if Ks = 0 or if the two aligned sequences existed as a perfect 100% match (Table S11). Next, to identify amino acid sites under the true occurrence of positive selection, program CODEML from PAML package v.4.8a [35] was used, with each corresponding protein coding sequence of chloroplast genome of *G. lancangensis* as the reference. A Bayes empirical bayes (BEB) approach [71] was then used to calculate posterior probabilities that a site came from the site class with ω > 1 by a site specific model 8 (β and ω). In the BEB analysis, posterior probability higher than 0.95 and 0.99 indicated sites that were under positive selection and strong positive selection, respectively (Table 4, Table S12).

### Phylogenetic tree analyses

To reconstruct the phylogenetic relationships and analyze the phylogenetic positions of 5 *Globba* species, 4 *Hedychium* species, 3 *Kaempferia* species and 5 *Zingiber* species within subfamily Zingiberoideae, 56 complete chloroplast genomes of family Zingiberaceae were used for analysis (Table S13). A total of 49 complete chloroplast genomes were downloaded from the GenBank database. *C. pulverulentus*, *C. viridis* and *C. indica* were used as the outgroups. The phylogenetic trees were constructed using an SNP matrix from 56 chloroplast genomes of family Zingiberaceae and 3 chloroplast genomes of outgroups, using the chloroplast genome of *G. lancangensis* as the reference. Reliable SNP sites were obtained using a previously described method that utilized MUMmer and BLAT software [19, 21, 26, 72–74]. For each chloroplast genome, all SNP sites were connected in the same order to obtain a sequence in FASTA format. Each connected FASTA format sequence contained 11,346 SNP markers (Table S14). Multiple FASTA format sequence alignments were performed with ClustalW in MEGA v.7 [32]. The ML analysis of MEGA v.7 was used for the reconstruction of the ML phylogenetic tree with default settings including 1000 bootstrap replications along with the nucleotide substitution model of Tamura-Nei [32] (Fig. 8). Bootstrap values were categorized as strong (> 85%), moderate (70–85%), weak (50–70%), or poor (< 50%) [2]. Lastly, BI analysis was performed using MrBayes v.3.2 [75], using the substitution model GTR with running parameters: the Markov Chain Monte Carlo algorithm was applied for 2 million generations with four Markov chains and sampled of trees every 100 generations, then the first 10% of trees were discarded as burn-in. The software iTOL v.3.4.3 (http://itol.embl.de/itol.cgi) was used to edit and visualize the final BI tree (Fig. S3).

## Supplementary Information

**Additional file 1: Table S1.** Characteristics of 10 assembled chloroplast genomes in subfamily Zingiberoideae.

**Additional file 2: Table S2.** Genes distribution in the 10 assembled chloroplast genomes in subfamily Zingiberoideae. **a** *G. lancangensis.* **b** *G. marantina.* **c** *G. multiflora.* **d** *G. schomburgkii.* **e** *G. schomburgkii var. angustata.* **f** *H. coccineum.* **g** *H. neocarneum*. **h** *K. rotunda* ‘Red Leaf’. **i** *K. rotunda* ‘Silver Diamonds’. **j** *Z. recurvatum.*

**Additional file 3: Table S3.** Genes with introns in 10 assembled chloroplast genomes of subfamily Zingiberoideae.

**Additional file 4: Table S4.** Codon content of all the protein coding genes of 10 assembled chloroplast genomes in subfamily Zingiberoideae.

**Additional file 5: Table S5.** Codon usages of all protein coding genes in 10 assembled chloroplast genomes in subfamily Zingiberoideae.

**Additional file 6: Table S6.** List of RNA editing sites in 10 assembled chloroplast genomes of subfamily Zingiberoideae as predicted by the PREP program [67].

**Additional file 7: Table S7.** Details of simple sequence repeats (SSRs) types distribution and abundance in 10 Zingiberoideae chloroplast genomes.

**Additional file 8: Table S8.** Statistics of SSRs sequences distribution and designed primers in 10 assembled chloroplast genomes of subfamily Zingiberoideae. **a** *G. lancangensis.* **b** *G. marantina.* **c** *G. multiflora.* **d** *G. schomburgkii.* **e** *G. schomburgkii var. angustata.* **f** *H. coccineum.* **g** *H. neocarneum.* **h** *K. rotunda* ‘Red Leaf’. **i** *K. rotunda* ‘Silver Diamonds’. **j** *Z. recurvatum.* p1: mononucleotide repeat; p2: dinucleotide repeat; p3: trinucleotide repeat; p4: tetranucleotide repeat; p5: pentanucleotide repeat; p6: hexanucleotide repeat; c: composite SSR.

**Additional file 9: Table S9.** Comparison of the long repeats among 10 assembled Zingiberoideae chloroplast genomes. **a** *G. lancangensis.* **b** *G. marantina.* **c** *G. multiflora.* **d** *G. schomburgkii.* **e** *G. schomburgkii var. angustata.* **f** *H. coccineum.* **g** *H. neocarneum.* **h** *K. rotunda* ‘Red Leaf’. **i** *K. rotunda* ‘Silver Diamonds’. **j** *Z. recurvatum.* **k** Total long repeat numbers of 10 assembled Zingiberoideae chloroplast genomes.

**Additional file 10: Table S10.** Nucleotide diversity (Pi) analysis of five *Globba* species, four *Hedychium* species, three *Kaempferia* species and five *Zingiber* species chloroplast genomes in subfamily Zingiberoideae computed by DnaSP V.6 [69]. **a** Pi values of 160 protein coding regions. The *rps12* was classified as three parts as the second gene divided into three independent transcription units. **b** Pi values of 161 intron and intergenic regions.

**Additional file 11: Table S11.** The rates of Ka, Ks and Ka/Ks of 79 protein coding genes in 17 Zingiberoideae species by comparing pairwise computed by KaKs_Calculator with default parameters [70]. **a** Total comparison rates of Ka, Ks and Ka/Ks in 17 Zingiberoideae species. When the Ks values were notably low or the two aligned sequences exhibited 100% perfect, the values of Ka/Ks were replaced by NA. **b** Total comparison rates of Ka, Ks and Ka/Ks in 17 Zingiberoideae species with Ka/Ks > 1.00 and *p* values < 0.05.

**Additional file 12: Table S12.** Details of positively selected amino acid loci of 14 protein coding genes in 17 Zingiberoideae species computed by PAML [35].

**Additional file 13: Table S13.** The 59 chloroplast genomes used for phylogenetic analysis.

**Additional file 14: Table S14.** The 59 chloroplast genomes’ SNP matrix data.

**Additional file 15: Figure S1.** Gene maps of the other 9 assembled Zingiberoideae chloroplast genomes in this study. Genes shown inside the circle are transcribed clockwise, and those outside are transcribed counterclockwise. The gray arrowheads indicate the direction of the genes. Different genes are color coded. The innermost darker gray corresponds to GC content, whereas the lighter gray corresponds to AT content. The inner circle also indicates that the chloroplast genome contains a large single copy region (LSC), a small single copy region (SSC) and two copies of the inverted repeat (IRA and IRB). **a** *G. marantina.* **b** *G. multiflora.* **c** G. schomburgkii.* indicates the two sites of the two genes *trnS-GCU* and *trnT-UGU* only present in *G. schomburgkii* instead of the two genes *trnS-GGA* and *trnT-GGU*, respectively. **d** *G. schomburgkii var. angustata.* **e** H. coccineum.* indicates the site of the gene *psbZ* present in *H. coccineum* instead of the gene *lhbA.* **f** *H. neocarneum.* * indicates the site of the gene *psbZ* present in *H. neocarneum* instead of the gene *lhbA.* **g** *K. rotunda* ‘Red Leaf’. **h** *K. rotunda* ‘Silver Diamonds’. **i** *Z. recurvatum.*

**Additional file 16: Figure S2.** Maximum likelihood (ML) trees of five *Globba* species, four *Hedychium* species, three *Kaempferia* species and five *Zingiber* species based on the chloroplast genomes divergent genes and intergenic regions. **a** ML tree based on the sequences of gene *matK*. **b** ML tree based on the sequences of gene *ndhF*. **c** ML tree based on the sequences of gene *ycf1*. **d** ML tree based on the intergenic sequences of *trnK-UUU-CDS1*-*rps16-CDS2*. **e** ML tree based on the intergenic sequences of *psaJ*-*rpl33*. **f** ML tree based on the intergenic sequences of *ycf4-cemA*. **g** ML tree based on the intergenic sequences of *trnT-UGU-trnL-UAA-CDS1*. **h** ML tree based on the intergenic sequences of *trnT-GGU-psbD*. **i** ML tree based on the intergenic sequences of *rpl32*-*trnL-UAG*. **j** ML tree based on the intergenic sequences of *psbM-trnD-GUC*. **k** ML tree based on the intergenic sequences of *ndhF-rpl32*. **l** ML tree based on the intergenic sequences of *rps15-ycf1*. **m** ML tree based on the intergenic sequences of *ccsA-ndhD*.

**Additional file 17: Figure S3.** Molecular phylogenetic tree based on the SNPs from 56 chloroplast genomes of family Zingiberaceae using Bayesian inference. The numbers at the nodes were Bayesian inference posterior probabilities. All nodes of the tree were supported by 1.00 Bayesian inference posterior probability. The branch length was proportional to the inferred divergence level and the scale bar indicated the number of inferred nucleic acids substitutions per site. *C. indica*, *C. pulverulentus* and *C. viridis* were used as the outgroups.

## Data Availability

All the ten sequenced complete chloroplast genomes in this study have been submitted to NCBI (https://www.ncbi.nlm.nih.gov) with accession numbers MK262735, and MT473704 - MT473712 (see Table 1). These data will remain private until the related manuscript has been accepted. Other chloroplast genomes for phylogenetic analysis can be obtained from NCBI, and their accession numbers are listed in Table S13. All other data generated in this manuscript are available from the corresponding author upon reasonable request.

## Abbreviations

bp: Base pairs
BLAST: Basic Local Alignment Search Tool
NCBI: National Center for Biotechnology Information
IR: Inverted repeat
LSC: Large single copy
SSC: Small single copy
SSRs: Simple Sequence Repeats
A: Adenine
T: Thymine
G: Guanine
C: Cytosine
DNA: Deoxyribonucleic Acid
RNA: Ribonucleic acid
tRNA: Transfer RNA
rRNA: Ribosomal RNA
SNPs: Single-nucleotide polymorphisms
