# Supplementary material for: Molecular evolution of chloroplast genomes in subfamily Zingiberoideae (Zingiberaceae)
Source: BMC Plant Biol. 2021 Nov 23;21:558. doi: 10.1186/s12870-021-03315-9 (PMC8611967; doi:10.1186/s12870-021-03315-9)
Supplement: Supplementary file 1 — Additional file 1: Table S1. Characteristics of 10 assembled chloroplast genomes in subfamily Zingiberoideae. [file 12870_2021_3315_MOESM1_ESM.docx]

**Table S1. Characteristics of ten assembled chloroplast genomes in subfamily Zingiberoideae.**

| **Taxa** | | **Regions** | | **Positions** | | **Length (bp)** | **T/U (%)** | | **C (%)** | **A (%)** | **G (%)** | **AT/U (%)** |
| --- | --- | --- | --- | --- | --- | --- | --- | --- | --- | --- | --- | --- |
| *G. lancangensis* | | Genome | |  | | 163,306 | 32.4 | | 18.18 | 31.87 | 17.56 | 64.27 |
|  |  | LSC | |  | | 88,545 | 33.93 | | 17.09 | 32.72 | 16.26 | 66.65 |
|  |  | IRa | |  | | 29,684 | 28.83 | | 19.78 | 30.14 | 21.24 | 58.98 |
|  |  | SSC | |  | | 15,393 | 34.8 | | 15.45 | 36.13 | 13.62 | 70.93 |
|  |  | IRb | |  | | 29,684 | 30.13 | | 21.25 | 28.84 | 19.78 | 58.97 |
|  |  | Protein coding genes | |  | | 83,039 | 31.71 | | 17.01 | 31.7 | 19.58 | 63.41 |
|  |  |  | | 1st position | | 27,679 | 27.86 | | 18.83 | 30.57 | 22.74 | 58.44 |
|  |  |  | | 2nd position | | 27,679 | 34.66 | | 16.97 | 31.19 | 17.19 | 65.85 |
|  |  |  | | 3rd position | | 27,679 | 32.62 | | 15.22 | 33.33 | 18.83 | 65.95 |
|  |  | tRNA | |  | | 2,875 | 25.08 | | 23.58 | 21.98 | 29.36 | 47.06 |
|  |  | rRNA | |  | | 9,048 | 18.72 | | 23.56 | 26.22 | 31.5 | 44.94 |
| *G. marantina* | | Genome | |  | | 162,774 | 32.3 | | 18.28 | 31.78 | 17.64 | 64.08 |
|  |  | LSC | |  | | 87,989 | 33.83 | | 17.22 | 32.59 | 16.36 | 66.42 |
|  |  | IRa | |  | | 29,680 | 28.81 | | 19.8 | 30.12 | 21.27 | 58.93 |
|  |  | SSC | |  | | 15,425 | 34.5 | | 15.62 | 36.1 | 13.78 | 70.60 |
|  |  | IRb | |  | | 29,680 | 30.11 | | 21.27 | 28.8 | 19.81 | 58.92 |
|  |  | Protein coding genes | |  | | 83,137 | 31.68 | | 17.03 | 31.64 | 19.65 | 63.32 |
|  |  |  | | 1st position | | 27,712 | 27.11 | | 16.96 | 31.52 | 24.4 | 58.64 |
|  |  |  | | 2nd position | | 27,712 | 30.44 | | 19.54 | 30.8 | 19.21 | 61.25 |
|  |  |  | | 3rd position | | 27,712 | 37.48 | | 14.59 | 32.6 | 15.33 | 70.08 |
|  |  | tRNA | |  | | 2,861 | 25.06 | | 23.66 | 21.99 | 29.29 | 47.05 |
|  |  | rRNA | |  | | 9,052 | 18.69 | | 23.6 | 26.23 | 31.48 | 44.92 |
| *G. multiflora* | Genome | |  | | 163,199 | | | 32.36 | 18.23 | 31.8 | 17.62 | 64.15 |
|  | LSC | |  | | 87,994 | | | 33.84 | 17.19 | 32.56 | 16.41 | 66.40 |
|  | IRa | |  | | 29,745 | | | 28.84 | 19.77 | 30.14 | 21.25 | 58.98 |
|  | SSC | |  | | 15,715 | | | 34.9 | 15.39 | 36.27 | 13.44 | 71.17 |
|  | IRb | |  | | 29,742 | | | 30.13 | 21.25 | 28.84 | 19.77 | 58.97 |
|  | Protein coding genes | |  | | 83,185 | | | 31.67 | 17.05 | 31.65 | 19.63 | 63.32 |
|  |  | | 1st position | | 27,728 | | | 27.12 | 16.97 | 31.53 | 24.38 | 58.65 |
|  |  | | 2nd position | | 27,728 | | | 30.4 | 19.57 | 30.87 | 19.16 | 61.27 |
|  |  | | 3rd position | | 27,728 | | | 37.5 | 14.61 | 32.56 | 15.33 | 70.05 |
|  | tRNA | |  | | 2,861 | | | 24.96 | 23.73 | 21.95 | 29.36 | 46.91 |
|  | rRNA | |  | | 9,050 | | | 18.72 | 23.56 | 26.25 | 31.47 | 44.97 |
| *G. schomburgkii* | Genome | |  | | 163,325 | | | 32.33 | 18.25 | 31.82 | 17.6 | 64.15 |
|  | LSC | |  | | 88,451 | | | 33.84 | 17.19 | 32.65 | 16.32 | 66.49 |
|  | IRa | |  | | 29,673 | | | 28.81 | 19.82 | 30.1 | 21.27 | 58.91 |
|  | SSC | |  | | 15,525 | | | 34.71 | 15.48 | 36.12 | 13.69 | 70.83 |
|  | IRb | |  | | 29,676 | | | 28.8 | 19.81 | 30.09 | 21.29 | 58.90 |
|  | Protein coding genes | |  | | 83,190 | | | 31.68 | 17.05 | 31.64 | 19.63 | 63.32 |
|  |  | | 1st position | | 27,730 | | | 24.04 | 18.13 | 31.67 | 26.16 | 55.71 |
|  |  | | 2nd position | | 27,730 | | | 32.48 | 19.94 | 30.26 | 17.32 | 62.74 |
|  |  | | 3rd position | | 27,730 | | | 38.5 | 13.09 | 33 | 15.4 | 71.51 |
|  | tRNA | |  | | 2,877 | | | 25.1 | 23.64 | 22 | 29.27 | 47.10 |
|  | rRNA | |  | | 9,046 | | | 18.73 | 23.62 | 26.16 | 31.49 | 44.88 |

**Table S1. Continued.**

| **Taxa** | | **Regions** | **Positions** | **Length (bp)** | | **T/U (%)** | **C (%)** | **A (%)** | **G (%)** | **AT/U (%)** |
| --- | --- | --- | --- | --- | --- | --- | --- | --- | --- | --- |
| *G. schomburgkii* *var. angustata* | | Genome |  | 163,432 | | 32.35 | 18.24 | 31.82 | 17.59 | 64.17 |
|  |  | LSC |  | 88,556 | | 33.88 | 17.17 | 32.65 | 16.3 | 66.53 |
|  |  | IRa |  | 29,675 | | 28.82 | 19.81 | 30.1 | 21.27 | 58.91 |
|  |  | SSC |  | 15,526 | | 34.71 | 15.48 | 36.13 | 13.68 | 70.84 |
|  |  | IRb |  | 29,678 | | 30.09 | 21.29 | 28.81 | 19.81 | 58.90 |
|  |  | Protein coding genes |  | 83,105 | | 31.67 | 17.03 | 31.64 | 19.65 | 63.32 |
|  |  |  | 1st position | 27,701 | | 26.44 | 18.38 | 31.18 | 24.01 | 57.61 |
|  |  |  | 2nd position | 27,701 | | 33.31 | 18.13 | 30.92 | 17.64 | 64.23 |
|  |  |  | 3rd position | 27,701 | | 35.27 | 14.59 | 32.83 | 17.31 | 68.09 |
|  |  | tRNA |  | 2,861 | | 25.06 | 23.7 | 21.99 | 29.26 | 47.05 |
|  |  | rRNA |  | 9,050 | | 18.7 | 23.6 | 26.21 | 31.49 | 44.91 |
| *H. coccineum* | | Genome |  | 163,968 | | 32.23 | 18.36 | 31.69 | 17.72 | 63.92 |
|  |  | LSC |  | 88,632 | | 33.70 | 17.35 | 32.47 | 16.48 | 66.17 |
|  |  | IRa |  | 29,769 | | 28.81 | 19.84 | 30.04 | 21.31 | 58.85 |
|  |  | SSC |  | 15,798 | | 34.57 | 15.60 | 35.87 | 13.96 | 70.44 |
|  |  | IRb |  | 29,769 | | 30.04 | 21.31 | 28.81 | 19.84 | 58.85 |
|  |  | Protein coding genes |  | 79,248 | | 31.64 | 17.27 | 31.13 | 19.95 | 62.78 |
|  |  |  | 1st position | 26,416 | | 23.90 | 18.33 | 31.19 | 26.58 | 55.10 |
|  |  |  | 2nd position | 26,416 | | 32.52 | 20.14 | 29.69 | 17.66 | 62.21 |
|  |  |  | 3rd position | 26,416 | | 38.50 | 13.36 | 32.52 | 15.62 | 71.03 |
|  |  | tRNA |  | 2,862 | | 24.95 | 23.76 | 21.94 | 29.35 | 46.89 |
|  |  | rRNA |  | 9,046 | | 18.68 | 23.62 | 26.17 | 31.53 | 44.85 |
| *H. neocarneum* | Genome | |  | 163,903 | 32.24 | | 18.35 | 31.68 | 17.73 | 63.92 |
|  | LSC | |  | 88,541 | 33.72 | | 17.36 | 32.43 | 16.49 | 66.15 |
|  | IRa | |  | 29,769 | 28.80 | | 19.84 | 30.05 | 21.31 | 58.85 |
|  | SSC | |  | 15,824 | 34.58 | | 15.58 | 35.91 | 13.93 | 70.49 |
|  | IRb | |  | 29,769 | 30.04 | | 21.31 | 28.80 | 19.84 | 58.85 |
|  | Protein coding genes | |  | 79,251 | 31.64 | | 17.27 | 31.13 | 19.95 | 62.78 |
|  |  | | 1st position | 26,417 | 23.91 | | 18.33 | 31.20 | 26.57 | 55.10 |
|  |  | | 2nd position | 26,417 | 32.52 | | 20.13 | 29.68 | 17.67 | 62.21 |
|  |  | | 3rd position | 26,417 | 38.50 | | 13.36 | 32.52 | 15.62 | 71.02 |
|  | tRNA | |  | 2,862 | 24.95 | | 23.76 | 21.94 | 29.35 | 46.89 |
|  | rRNA | |  | 9,046 | 18.68 | | 23.62 | 26.17 | 31.53 | 44.85 |
| *K. rotunda* ‘Red Leaf’ | Genome | |  | 162,630 | 32.15 | | 18.4 | 31.67 | 17.78 | 63.82 |
|  | LSC | |  | 87,172 | 33.56 | | 17.43 | 32.43 | 16.59 | 65.98 |
|  | IRa | |  | 29,829 | 28.85 | | 19.79 | 30.07 | 21.29 | 58.92 |
|  | SSC | |  | 15,800 | 34.55 | | 15.68 | 35.85 | 13.92 | 70.40 |
|  | IRb | |  | 29,833 | 30.06 | | 21.29 | 28.85 | 19.8 | 58.91 |
|  | Protein coding genes | |  | 83,167 | 31.55 | | 17.2 | 31.52 | 19.74 | 63.06 |
|  |  | | 1st position | 27,722 | 27.4 | | 16.99 | 31.4 | 24.2 | 58.80 |
|  |  | | 2nd position | 27,722 | 30.12 | | 19.41 | 30.99 | 19.48 | 61.11 |
|  |  | | 3rd position | 27,722 | 37.12 | | 15.19 | 32.15 | 15.54 | 69.27 |
|  | tRNA | |  | 2,861 | 24.96 | | 23.77 | 22.02 | 29.26 | 46.98 |
|  | rRNA | |  | 9,044 | 18.69 | | 23.6 | 26.14 | 31.58 | 44.83 |

**Table S1. Continued.**

| **Taxa** | **Regions** | | **Positions** | **Length (bp)** | | **T/U (%)** | **C (%)** | **A (%)** | | **G (%)** | | **AT/U (%)** | |
| --- | --- | --- | --- | --- | --- | --- | --- | --- | --- | --- | --- | --- | --- |
| *K. rotunda* ‘Silver Diamonds’ | Genome | |  | 162,875 | 32.19 | | 18.38 | 31.68 | 17.76 | | 63.87 | |  |
|  | LSC | |  | 87,306 | 33.6 | | 17.41 | 32.43 | 16.56 | | 66.03 | |  |
|  | IRa | |  | 29,826 | 28.87 | | 19.8 | 30.04 | 21.29 | | 58.92 | |  |
|  | SSC | |  | 15,917 | 34.62 | | 15.57 | 35.94 | 13.87 | | 70.56 | |  |
|  | IRb | |  | 29,826 | 30.04 | | 21.29 | 28.86 | 19.8 | | 58.91 | |  |
|  | Protein coding genes | |  | 83,166 | 31.55 | | 17.2 | 31.49 | 19.75 | | 63.04 | |  |
|  |  | | 1st position | 27,722 | 23.96 | | 18.24 | 31.52 | 26.28 | | 55.48 | |  |
|  |  | | 2nd position | 27,722 | 32.41 | | 20.01 | 30.19 | 17.39 | | 62.60 | |  |
|  |  | | 3rd position | 27,722 | 38.29 | | 13.35 | 32.76 | 15.59 | | 71.05 | |  |
|  | tRNA | |  | 2,861 | 24.92 | | 23.8 | 21.95 | 29.33 | | 46.87 | |  |
|  | rRNA | |  | 9,044 | 18.71 | | 23.6 | 26.12 | 31.58 | | 44.83 | |  |
| *Z. recurvatum* | | Genome |  | 163,151 | | 32.22 | 18.38 | 31.65 | 17.74 | | 63.88 | |  |
|  |  | LSC |  | 87,780 | | 33.69 | 17.37 | 32.4 | 16.54 | | 66.09 | |  |
|  |  | IRa |  | 29,792 | | 28.85 | 19.86 | 30.01 | 21.27 | | 58.86 | |  |
|  |  | SSC |  | 15,787 | | 34.61 | 15.73 | 35.86 | 13.8 | | 70.47 | |  |
|  |  | IRb |  | 29,792 | | 30.01 | 21.27 | 28.84 | 19.86 | | 58.86 | |  |
|  |  | Protein coding genes |  | 83,141 | | 31.55 | 17.19 | 31.5 | 19.76 | | 63.05 | |  |
|  |  |  | 1st position | 27,713 | | 26.22 | 18.84 | 30.74 | 24.2 | | 56.96 | |  |
|  |  |  | 2nd position | 27,713 | | 33.6 | 18.16 | 30.84 | 17.4 | | 64.44 | |  |
|  |  |  | 3rd position | 27,713 | | 34.83 | 14.58 | 32.92 | 17.67 | | 67.75 | |  |
|  |  | tRNA |  | 2,860 | | 24.93 | 23.81 | 21.96 | 29.3 | | 46.89 | |  |
|  |  | rRNA |  | 9,048 | | 18.68 | 23.63 | 26.15 | 31.54 | | 44.83 | |  |
