# Supplementary material for: Molecular evolution of chloroplast genomes in subfamily Zingiberoideae (Zingiberaceae)
Source: BMC Plant Biol. 2021 Nov 23;21:558. doi: 10.1186/s12870-021-03315-9 (PMC8611967; doi:10.1186/s12870-021-03315-9)
Supplement: Supplementary file 3 — Additional file 3: Table S3. Genes with introns in 10 assembled chloroplast genomes of subfamily Zingiberoideae. [file 12870_2021_3315_MOESM3_ESM.docx]

**Table S3.** Genes with introns in ten assembled chloroplast genomes of Zingiberoideae.

| **Species** | **Gene** | **Location** | **Exon І (bp)** | **Intron І (bp)** | **Exon ІІ (bp)** | **Intron ІІ (bp)** | **Exon ІІІ (bp)** |
| --- | --- | --- | --- | --- | --- | --- | --- |
| *G. lancangensis* | *trnA-UGC* | IR | 38 | 801 | 35 |  |  |
|  | *trnI-GAU* | IR | 42 | 935 | 35 |  |  |
|  | *trnG-GCC* | LSC | 14 | 726 | 48 |  |  |
|  | *trnK-UUU* | LSC | 35 | 2662 | 37 |  |  |
|  | *trnL-UAA* | LSC | 35 | 520 | 50 |  |  |
|  | *trnV-UAC* | LSC | 37 | 592 | 36 |  |  |
|  | *rps12** | LSC/IR | 114 | - | 27 | 540 | 231 |
|  | *rps16* | LSC | 212 | 731 | 40 |  |  |
|  | *rpl2* | IR | 432 | 659 | 387 |  |  |
|  | *rpl16* | LSC | 402 | 1055 | 9 |  |  |
|  | *petB* | LSC | 6 | 790 | 642 |  |  |
|  | *petD* | LSC | 8 | 748 | 481 |  |  |
|  | *atpF* | LSC | 411 | 830 | 144 |  |  |
|  | *ndhA* | SSC | 540 | 1070 | 552 |  |  |
|  | *ndhB* | IR | 756 | 700 | 723 |  |  |
|  | *rpoC1* | LSC | 1632 | 724 | 432 |  |  |
|  | *clpP* | LSC | 255 | 650 | 291 | 872 | 69 |
|  | *ycf3* | LSC | 153 | 786 | 228 | 717 | 132 |
| *G. marantina* | *trnA-UGC* | IR | 38 | 801 | 35 |  |  |
|  | *trnI-GAU* | IR | 42 | 935 | 35 |  |  |
|  | *trnK-UUU* | LSC | 35 | 2669 | 37 |  |  |
|  | *trnL-UAA* | LSC | 35 | 545 | 50 |  |  |
|  | *trnV-UAC* | LSC | 37 | 592 | 36 |  |  |
|  | *rps12** | LSC/IR | 114 | - | 27 | 540 | 231 |
|  | *rps16* | LSC | 212 | 743 | 40 |  |  |
|  | *rpl2* | IR | 432 | 659 | 387 |  |  |
|  | *rpl16* | LSC | 402 | 1053 | 9 |  |  |
|  | *petB* | LSC | 6 | 782 | 642 |  |  |
|  | *petD* | LSC | 8 | 748 | 481 |  |  |
|  | *atpF* | LSC | 411 | 818 | 144 |  |  |
|  | *ndhA* | SSC | 540 | 1072 | 552 |  |  |
|  | *ndhB* | IR | 756 | 700 | 777 |  |  |
|  | *rpoC1* | LSC | 1632 | 725 | 432 |  |  |
|  | *clpP* | LSC | 255 | 642 | 291 | 883 | 69 |
|  | *ycf3* | LSC | 153 | 787 | 228 | 717 | 132 |
| *G. multiflora* | *trnA-UGC* | IR | 38 | 801 | 35 |  |  |
|  | *trnI-GAU* | IR | 42 | 935 | 35 |  |  |
|  | *trnK-UUU* | LSC | 35 | 2653 | 37 |  |  |
|  | *trnL-UAA* | LSC | 35 | 535 | 50 |  |  |
|  | *trnV-UAC* | LSC | 37 | 592 | 36 |  |  |
|  | *rps12** | LSC/IR | 114 | - | 27 | 540 | 231 |
|  | *rps16* | LSC | 212 | 743 | 40 |  |  |
|  | *rpl2* | IR | 432 | 659 | 387 |  |  |
|  | *rpl16* | LSC | 402 | 1068 | 9 |  |  |
|  | *petB* | LSC | 6 | 789 | 642 |  |  |
|  | *petD* | LSC | 8 | 748 | 481 |  |  |
|  | *atpF* | LSC | 411 | 812 | 144 |  |  |
|  | *ndhA* | SSC | 541 | 1053 | 551 |  |  |
|  | *ndhB* | IR | 756 | 700 | 777 |  |  |
|  | *rpoC1* | LSC | 1632 | 726 | 432 |  |  |
|  | *clpP* | LSC | 240 | 632 | 291 | 876 | 69 |
|  | *ycf3* | LSC | 153 | 791 | 228 | 718 | 132 |

**Table S3. Continued.**

| **Species** | **Gene** | **Location** | **Exon І (bp)** | **Intron І (bp)** | **Exon ІІ (bp)** | **Intron ІІ (bp)** | **Exon ІІІ (bp)** |
| --- | --- | --- | --- | --- | --- | --- | --- |
| *G. schomburgkii* | *trnA-UGC* | IR | 38 | 801 | 35 |  |  |
|  | *trnI-GAU* | IR | 42 | 935 | 35 |  |  |
|  | *trnG-GCC* | LSC | 14 | 717 | 48 |  |  |
|  | *trnK-UUU* | LSC | 35 | 2689 | 37 |  |  |
|  | *trnL-UAA* | LSC | 35 | 540 | 50 |  |  |
|  | *trnV-UAC* | LSC | 37 | 590 | 38 |  |  |
|  | *rps12 ** | LSC/IR | 114 | - | 27 | 540 | 231 |
|  | *rps16* | LSC | 212 | 738 | 40 |  |  |
|  | *rpl2* | IR | 443 | 650 | 385 |  |  |
|  | *rpl16* | LSC | 402 | 1071 | 9 |  |  |
|  | *petB* | LSC | 6 | 792 | 642 |  |  |
|  | *petD* | LSC | 8 | 748 | 481 |  |  |
|  | *atpF* | LSC | 425 | 798 | 145 |  |  |
|  | *ndhA* | SSC | 518 | 1092 | 562 |  |  |
|  | *ndhB* | IR | 778 | 676 | 779 |  |  |
|  | *rpoC1* | LSC | 1632 | 725 | 432 |  |  |
|  | *clpP* | LSC | 252 | 651 | 306 | 877 | 60 |
|  | *ycf3* | LSC | 167 | 780 | 201 | 741 | 124 |
| *G. schomburgkii* *var. angustata* | *trnA-UGC* | IR | 38 | 801 | 35 |  |  |
|  | *trnI-GAU* | IR | 42 | 935 | 35 |  |  |
|  | *trnK-UUU* | LSC | 35 | 2689 | 37 |  |  |
|  | *trnL-UAA* | LSC | 35 | 540 | 50 |  |  |
|  | *trnV-UAC* | LSC | 37 | 590 | 36 |  |  |
|  | *rps12 ** | LSC/IR | 114 | - | 27 | 540 | 231 |
|  | *rps16* | LSC | 212 | 738 | 40 |  |  |
|  | *rpl2* | IR | 432 | 659 | 387 |  |  |
|  | *rpl16* | LSC | 402 | 1071 | 9 |  |  |
|  | *petB* | LSC | 6 | 792 | 642 |  |  |
|  | *petD* | LSC | 8 | 748 | 481 |  |  |
|  | *atpF* | LSC | 411 | 813 | 144 |  |  |
|  | *ndhA* | SSC | 541 | 1080 | 551 |  |  |
|  | *ndhB* | IR | 756 | 700 | 776 |  |  |
|  | *rpoC1* | LSC | 1632 | 725 | 432 |  |  |
|  | *clpP* | LSC | 255 | 648 | 291 | 883 | 69 |
|  | *ycf3* | LSC | 153 | 786 | 228 | 714 | 132 |
| *H. coccineum* | *trnA-UGC* | IR | 38 | 801 | 35 |  |  |
|  | *trnI-GAU* | IR | 42 | 935 | 35 |  |  |
|  | *trnK-UUU* | LSC | 35 | 2638 | 37 |  |  |
|  | *trnL-UAA* | LSC | 35 | 531 | 50 |  |  |
|  | *trnV-UAC* | LSC | 37 | 600 | 36 |  |  |
|  | *rps12 ** | LSC/IR | 114 | - | 27 | 540 | 231 |
|  | *rps16* | LSC | 212 | 737 | 40 |  |  |
|  | *rpl2* | IR | 432 | 659 | 387 |  |  |
|  | *rpl16* | LSC | 402 | 1040 | 9 |  |  |
|  | *petB* | LSC | 6 | 784 | 642 |  |  |
|  | *petD* | LSC | 8 | 738 | 481 |  |  |
|  | *atpF* | LSC | 411 | 825 | 144 |  |  |
|  | *ndhA* | SSC | 541 | 1065 | 551 |  |  |
|  | *ndhB* | IR | 756 | 700 | 777 |  |  |
|  | *rpoC1* | LSC | 1632 | 724 | 432 |  |  |
|  | *clpP* | LSC | 252 | 643 | 291 | 870 | 69 |
|  | *ycf3* | LSC | 155 | 794 | 226 | 716 | 132 |

**Table S3. Continued.**

| **Species** | **Gene** | **Location** | **Exon І (bp)** | **Intron І (bp)** | **Exon ІІ (bp)** | **Intron ІІ (bp)** | **Exon ІІІ (bp)** |
| --- | --- | --- | --- | --- | --- | --- | --- |
| *H. neocarneum* | *trnA-UGC* | IR | 38 | 801 | 35 |  |  |
|  | *trnI-GAU* | IR | 42 | 935 | 35 |  |  |
|  | *trnK-UUU* | LSC | 35 | 2638 | 37 |  |  |
|  | *trnL-UAA* | LSC | 35 | 523 | 50 |  |  |
|  | *trnV-UAC* | LSC | 37 | 600 | 36 |  |  |
|  | *rps12** | LSC/IR | 114 | - | 27 | 540 | 231 |
|  | *rps16* | LSC | 212 | 737 | 40 |  |  |
|  | *rpl2* | IR | 432 | 659 | 387 |  |  |
|  | *rpl16* | LSC | 402 | 1042 | 9 |  |  |
|  | *petB* | LSC | 6 | 784 | 642 |  |  |
|  | *petD* | LSC | 8 | 738 | 481 |  |  |
|  | *atpF* | LSC | 411 | 825 | 144 |  |  |
|  | *ndhA* | SSC | 541 | 1067 | 551 |  |  |
|  | *ndhB* | IR | 756 | 700 | 777 |  |  |
|  | *rpoC1* | LSC | 1632 | 723 | 432 |  |  |
|  | *clpP* | LSC | 252 | 641 | 294 | 870 | 69 |
|  | *ycf3* | LSC | 155 | 894 | 226 | 716 | 132 |
| *K. rotunda* ‘Red Leaf’ | *trnA-UGC* | IR | 38 | 801 | 35 |  |  |
|  | *trnI-GAU* | IR | 42 | 935 | 35 |  |  |
|  | *trnK-UUU* | LSC | 35 | 2697 | 37 |  |  |
|  | *trnL-UAA* | LSC | 35 | 533 | 50 |  |  |
|  | *trnV-UAC* | LSC | 37 | 600 | 36 |  |  |
|  | *rps12 ** | LSC/IR | 114 | - | 27 | 540 | 231 |
|  | *rps16* | LSC | 212 | 729 | 40 |  |  |
|  | *rpl2* | IR | 432 | 659 | 387 |  |  |
|  | *rpl16* | LSC | 402 | 1048 | 9 |  |  |
|  | *petB* | LSC | 6 | 793 | 642 |  |  |
|  | *petD* | LSC | 8 | 739 | 481 |  |  |
|  | *atpF* | LSC | 411 | 741 | 144 |  |  |
|  | *ndhA* | SSC | 540 | 1065 | 552 |  |  |
|  | *ndhB* | IR | 756 | 700 | 777 |  |  |
|  | *rpoC1* | LSC | 1632 | 723 | 432 |  |  |
|  | *clpP* | LSC | 255 | 627 | 291 | 862 | 69 |
|  | *ycf3* | LSC | 153 | 794 | 228 | 713 | 132 |
| *K. rotunda* ‘Silver Diamonds’ | *trnA-UGC* | IR | 38 | 801 | 35 |  |  |
|  | *trnI-GAU* | IR | 42 | 935 | 35 |  |  |
|  | *trnK-UUU* | LSC | 35 | 2682 | 37 |  |  |
|  | *trnL-UAA* | LSC | 35 | 535 | 50 |  |  |
|  | *trnV-UAC* | LSC | 37 | 600 | 36 |  |  |
|  | *rps12** | LSC/IR | 114 | - | 27 | 540 | 231 |
|  | *rps16* | LSC | 212 | 728 | 40 |  |  |
|  | *rpl2* | IR | 432 | 659 | 387 |  |  |
|  | *rpl16* | LSC | 402 | 1036 | 9 |  |  |
|  | *petB* | LSC | 6 | 808 | 642 |  |  |
|  | *petD* | LSC | 8 | 740 | 481 |  |  |
|  | *atpF* | LSC | 411 | 810 | 144 |  |  |
|  | *ndhA* | SSC | 539 | 1067 | 551 |  |  |
|  | *ndhB* | IR | 756 | 700 | 777 |  |  |
|  | *rpoC1* | LSC | 1632 | 723 | 432 |  |  |
|  | *clpP* | LSC | 255 | 630 | 291 | 867 | 69 |
|  | *ycf3* | LSC | 153 | 795 | 228 | 713 | 132 |

**Table S3. Continued.**

| **Species** | **Gene** | **Location** | **Exon І (bp)** | **Intron І (bp)** | **Exon ІІ (bp)** | **Intron ІІ (bp)** | **Exon ІІІ (bp)** |
| --- | --- | --- | --- | --- | --- | --- | --- |
| *Z. recurvatum* | *trnA-UGC* | IR | 38 | 801 | 35 |  |  |
|  | *trnI-GAU* | IR | 42 | 935 | 35 |  |  |
|  | *trnK-UUU* | LSC | 35 | 2683 | 37 |  |  |
|  | *trnL-UAA* | LSC | 35 | 533 | 50 |  |  |
|  | *trnV-UAC* | LSC | 37 | 600 | 36 |  |  |
|  | *rps12** | LSC/IR | 114 | - | 27 | 540 | 231 |
|  | *rps16* | LSC | 212 | 737 | 40 |  |  |
|  | *rpl2* | IR | 432 | 659 | 387 |  |  |
|  | *rpl16* | LSC | 402 | 1047 | 9 |  |  |
|  | *petB* | LSC | 6 | 788 | 642 |  |  |
|  | *petD* | LSC | 8 | 740 | 481 |  |  |
|  | *atpF* | LSC | 411 | 805 | 144 |  |  |
|  | *ndhA* | SSC | 540 | 1065 | 552 |  |  |
|  | *ndhB* | IR | 756 | 700 | 777 |  |  |
|  | *rpoC1* | LSC | 1632 | 723 | 432 |  |  |
|  | *clpP* | LSC | 255 | 623 | 291 | 870 | 69 |
|  | *ycf3* | LSC | 153 | 794 | 228 | 711 | 132 |

* The *rps12* gene is divided into 5′-*rps12* in the LSC region and 3′-*rps12* in the IR region.
