# Supplementary material for: Molecular evolution of chloroplast genomes in subfamily Zingiberoideae (Zingiberaceae)
Source: BMC Plant Biol. 2021 Nov 23;21:558. doi: 10.1186/s12870-021-03315-9 (PMC8611967; doi:10.1186/s12870-021-03315-9)
Supplement: Supplementary file 5 — Additional file 5: Table S5. Codon usages of all protein coding genes in 10 assembled chloroplast genomes in subfamily Zingiberoideae. [file 12870_2021_3315_MOESM5_ESM.docx]

**Table S5. Codon usages of all protein-coding genes in ten assembled sequenced chloroplast genomes in subfamily Zingiberoideae.**

| Amino acid | Codon | *G. lancangensis* | *G. marantina* | *G. multiflora* | *G. schomburgkii* | *G. schomburgkii* *var. angustata* | *H. coccineum* | *H. neocarneum* | *K. rotunda* ‘Red Leaf’ | *K. rotunda* ‘Silver Diamonds’ | *Z. recurvatum* |
| --- | --- | --- | --- | --- | --- | --- | --- | --- | --- | --- | --- |
|  |  | RSCU* | | | | | | | | | |
| Ala | GCA | 1.2574 | 1.2544 | 1.2487 | 1.2766 | 1.2525 | 1.2461 | 1.2461 | 1.2452 | 1.2452 | 1.2432 |
| Ala | GCC | 0.5808 | 0.5887 | 0.5963 | 0.585 | 0.5878 | 0.5966 | 0.5966 | 0.6138 | 0.6138 | 0.6201 |
| Ala | GCG | 0.3203 | 0.3136 | 0.3217 | 0.3198 | 0.319 | 0.3232 | 0.3232 | 0.3289 | 0.3289 | 0.3262 |
| Ala | GCT | 1.8413 | 1.8431 | 1.8332 | 1.8184 | 1.8404 | 1.8339 | 1.8339 | 1.812 | 1.812 | 1.8104 |
| Cys | TGC | 0.4169 | 0.4379 | 0.4407 | 0.4507 | 0.4379 | 0.4437 | 0.4488 | 0.4304 | 0.4304 | 0.4437 |
| Cys | TGT | 1.583 | 1.562 | 1.5592 | 1.5492 | 1.562 | 1.5562 | 1.5511 | 1.5695 | 1.5695 | 1.5562 |
| Asp | GAC | 0.3452 | 0.3471 | 0.351 | 0.3561 | 0.3493 | 0.3426 | 0.3426 | 0.3476 | 0.3479 | 0.3443 |
| Asp | GAT | 1.6547 | 1.6528 | 1.6489 | 1.6438 | 1.6506 | 1.6573 | 1.6573 | 1.6523 | 1.652 | 1.6556 |
| Glu | GAA | 1.5024 | 1.4989 | 1.5074 | 1.5122 | 1.4996 | 1.4943 | 1.4939 | 1.4943 | 1.4915 | 1.4848 |
| Glu | GAG | 0.4975 | 0.501 | 0.4925 | 0.4877 | 0.5003 | 0.5056 | 0.506 | 0.5056 | 0.5084 | 0.5151 |
| Phe | TTC | 0.6835 | 0.693 | 0.6937 | 0.6879 | 0.6912 | 0.7146 | 0.7138 | 0.7137 | 0.7154 | 0.7096 |
| Phe | TTT | 1.3164 | 1.3069 | 1.3062 | 1.312 | 1.3087 | 1.2853 | 1.2861 | 1.2862 | 1.2845 | 1.2903 |
| Gly | GGA | 1.6541 | 1.6489 | 1.6368 | 1.6574 | 1.6484 | 1.6331 | 1.6331 | 1.6478 | 1.6432 | 1.6385 |
| Gly | GGC | 0.3123 | 0.321 | 0.3135 | 0.321 | 0.3227 | 0.3197 | 0.3197 | 0.3222 | 0.3222 | 0.3212 |
| Gly | GGG | 0.6362 | 0.6374 | 0.6501 | 0.6308 | 0.6363 | 0.6578 | 0.6578 | 0.6444 | 0.6467 | 0.6563 |
| Gly | GGT | 1.3973 | 1.3926 | 1.3994 | 1.3906 | 1.3925 | 1.3893 | 1.3893 | 1.3855 | 1.3878 | 1.3838 |
| His | CAC | 0.4177 | 0.4106 | 0.4149 | 0.4121 | 0.4144 | 0.4149 | 0.4149 | 0.4213 | 0.4238 | 0.4346 |
| His | CAT | 1.5822 | 1.5893 | 1.585 | 1.5878 | 1.5855 | 1.585 | 1.585 | 1.5786 | 1.5761 | 1.5653 |
| Ile | ATA | 0.9854 | 0.9739 | 0.9816 | 0.9857 | 0.9743 | 0.9611 | 0.9607 | 0.9659 | 0.9625 | 0.9643 |
| Ile | ATC | 0.5375 | 0.5427 | 0.5376 | 0.5368 | 0.5404 | 0.5536 | 0.5534 | 0.554 | 0.5548 | 0.5492 |
| Ile | ATT | 1.4769 | 1.4833 | 1.4807 | 1.4773 | 1.4852 | 1.4851 | 1.4857 | 1.4799 | 1.4825 | 1.4864 |
| Lys | AAA | 1.4887 | 1.4774 | 1.486 | 1.4813 | 1.4798 | 1.481 | 1.4814 | 1.4706 | 1.4727 | 1.4807 |
| Lys | AAG | 0.5112 | 0.5225 | 0.5139 | 0.5186 | 0.5201 | 0.5189 | 0.5185 | 0.5293 | 0.5272 | 0.5192 |
| Leu | CTA | 0.8252 | 0.82 | 0.8221 | 0.8443 | 0.8289 | 0.8291 | 0.8266 | 0.8251 | 0.8245 | 0.8214 |
| Leu | CTC | 0.396 | 0.3913 | 0.4099 | 0.3845 | 0.3903 | 0.4134 | 0.4177 | 0.4038 | 0.4035 | 0.4074 |

**Table S5. Continued.**

| Amino acid | Codon | *G. lancangensis* | *G. marantina* | *G. multiflora* | *G. schomburgkii* | *G. schomburgkii* *var. angustata* | *H. coccineum* | *H. neocarneum* | *K. rotunda* ‘Red Leaf’ | *K. rotunda* ‘Silver Diamonds’ | *Z. recurvatum* |
| --- | --- | --- | --- | --- | --- | --- | --- | --- | --- | --- | --- |
|  |  | RSCU | | | | | | | | | |
| Leu | CTG | 0.3495 | 0.3451 | 0.3438 | 0.3427 | 0.3464 | 0.3387 | 0.3385 | 0.3357 | 0.3421 | 0.3461 |
| Leu | CTT | 1.2455 | 1.2532 | 1.2454 | 1.2497 | 1.25 | 1.269 | 1.2663 | 1.275 | 1.2719 | 1.2705 |
| Leu | TTA | 1.9336 | 1.9435 | 1.9177 | 1.9435 | 1.9385 | 1.887 | 1.8886 | 1.8939 | 1.8925 | 1.8926 |
| Leu | TTG | 1.25 | 1.2466 | 1.2608 | 1.2351 | 1.2456 | 1.2624 | 1.262 | 1.2662 | 1.2653 | 1.2617 |
| Met | ATG | 1 | 1 | 1 | 1 | 1 | 1 | 1 | 1 | 1 | 1 |
| Asn | AAC | 0.4567 | 0.4563 | 0.4626 | 0.4637 | 0.461 | 0.4716 | 0.4689 | 0.4663 | 0.4639 | 0.4611 |
| Asn | AAT | 1.5432 | 1.5436 | 1.5373 | 1.5362 | 1.5389 | 1.5283 | 1.531 | 1.5336 | 1.536 | 1.5388 |
| Pro | CCA | 1.1396 | 1.1386 | 1.1449 | 1.1418 | 1.138 | 1.1529 | 1.1539 | 1.1375 | 1.1359 | 1.1348 |
| Pro | CCC | 0.746 | 0.7441 | 0.7286 | 0.7243 | 0.7313 | 0.7488 | 0.7495 | 0.7571 | 0.7585 | 0.7702 |
| Pro | CCG | 0.4611 | 0.4651 | 0.4572 | 0.4567 | 0.4626 | 0.4633 | 0.4638 | 0.4653 | 0.4662 | 0.4613 |
| Pro | CCT | 1.6532 | 1.652 | 1.6691 | 1.677 | 1.6679 | 1.6348 | 1.6326 | 1.6398 | 1.6392 | 1.6334 |
| Gln | CAA | 1.5431 | 1.5342 | 1.5377 | 1.5421 | 1.5332 | 1.5256 | 1.5256 | 1.5315 | 1.531 | 1.5285 |
| Gln | CAG | 0.4568 | 0.4657 | 0.4622 | 0.4578 | 0.4667 | 0.4743 | 0.4743 | 0.4684 | 0.4689 | 0.4714 |
| Arg | AGA | 1.9635 | 2.0012 | 1.9949 | 1.9952 | 2.0012 | 1.9653 | 1.9641 | 1.9641 | 1.9666 | 1.9453 |
| Arg | AGG | 0.6456 | 0.6445 | 0.6561 | 0.6434 | 0.6483 | 0.6538 | 0.6534 | 0.6559 | 0.6555 | 0.6484 |
| Arg | CGA | 1.2762 | 1.2404 | 1.2595 | 1.2582 | 1.2367 | 1.278 | 1.2772 | 1.2711 | 1.2703 | 1.2708 |
| Arg | CGC | 0.336 | 0.3372 | 0.3318 | 0.3307 | 0.3372 | 0.338 | 0.3378 | 0.3372 | 0.3407 | 0.3428 |
| Arg | CGG | 0.404 | 0.4309 | 0.4148 | 0.4313 | 0.4309 | 0.4123 | 0.4158 | 0.4224 | 0.4222 | 0.436 |
| Arg | CGT | 1.3744 | 1.3454 | 1.3425 | 1.3409 | 1.3454 | 1.3523 | 1.3514 | 1.3489 | 1.3444 | 1.3565 |
| Ser | AGC | 0.2956 | 0.3051 | 0.2846 | 0.3055 | 0.3025 | 0.2992 | 0.2991 | 0.2994 | 0.3017 | 0.3023 |
| Ser | AGT | 1.2602 | 1.2667 | 1.2735 | 1.2357 | 1.268 | 1.2489 | 1.2511 | 1.2495 | 1.25 | 1.2466 |
| Ser | TCA | 1.2315 | 1.212 | 1.2161 | 1.233 | 1.2219 | 1.2143 | 1.2138 | 1.2149 | 1.2097 | 1.2178 |
| Ser | TCC | 0.976 | 0.9702 | 0.9746 | 0.9633 | 0.9711 | 0.9755 | 0.975 | 0.9875 | 0.9885 | 1.0047 |
| Ser | TCG | 0.5167 | 0.5211 | 0.5404 | 0.5119 | 0.5129 | 0.5352 | 0.5349 | 0.5297 | 0.5344 | 0.5412 |
| Ser | TCT | 1.7196 | 1.7245 | 1.7105 | 1.7504 | 1.7233 | 1.7266 | 1.7257 | 1.7188 | 1.7155 | 1.6871 |

**Table S5. Continued.**

| Amino acid | Codon | *G. lancangensis* | *G. marantina* | *G. multiflora* | *G. schomburgkii* | *G. schomburgkii* *var. angustata* | *H. coccineum* | *H. neocarneum* | *K. rotunda* ‘Red Leaf’ | *K. rotunda* ‘Silver Diamonds’ | *Z. recurvatum* |
| --- | --- | --- | --- | --- | --- | --- | --- | --- | --- | --- | --- |
|  |  | RSCU | | | | | | | | | |
| Thr | ACA | 1.2738 | 1.2872 | 1.2724 | 1.2837 | 1.2792 | 1.2762 | 1.2772 | 1.2672 | 1.2642 | 1.2578 |
| Thr | ACC | 0.7053 | 0.6947 | 0.7065 | 0.7005 | 0.6936 | 0.7117 | 0.7122 | 0.7087 | 0.7117 | 0.6974 |
| Thr | ACG | 0.4494 | 0.4571 | 0.467 | 0.4689 | 0.4624 | 0.4594 | 0.4598 | 0.4594 | 0.4654 | 0.4709 |
| Thr | ACT | 1.5714 | 1.5609 | 1.5538 | 1.5468 | 1.5645 | 1.5525 | 1.5507 | 1.5645 | 1.5585 | 1.5737 |
| Val | GTA | 1.5341 | 1.5449 | 1.5347 | 1.5478 | 1.5482 | 1.5365 | 1.5365 | 1.5319 | 1.5298 | 1.5432 |
| Val | GTC | 0.4475 | 0.4466 | 0.4497 | 0.4507 | 0.4475 | 0.4704 | 0.4704 | 0.4722 | 0.4659 | 0.4609 |
| Val | GTG | 0.5404 | 0.5393 | 0.5397 | 0.5485 | 0.5404 | 0.5567 | 0.5567 | 0.5509 | 0.5529 | 0.5565 |
| Val | GTT | 1.4778 | 1.4691 | 1.4757 | 1.4528 | 1.4637 | 1.4363 | 1.4363 | 1.4448 | 1.4512 | 1.4392 |
| Trp | TGG | 1 | 1 | 1 | 1 | 1 | 1 | 1 | 1 | 1 | 1 |
| Tyr | TAC | 0.4201 | 0.4225 | 0.4247 | 0.4269 | 0.4201 | 0.4274 | 0.4274 | 0.4396 | 0.4384 | 0.4229 |
| Tyr | TAT | 1.5798 | 1.5774 | 1.5752 | 1.573 | 1.5798 | 1.5725 | 1.5725 | 1.5603 | 1.5615 | 1.577 |
| STOP* | TAA | 1.6588 | 1.6744 | 1.6235 | 1.6896 | 1.6588 | 1.7093 | 1.7093 | 1.7441 | 1.7441 | 1.7441 |
| STOP* | TAG | 0.7411 | 0.7674 | 0.8117 | 0.7586 | 0.7764 | 0.7674 | 0.7674 | 0.7674 | 0.8023 | 0.8023 |
| STOP* | TGA | 0.6 | 0.5581 | 0.5647 | 0.5517 | 0.5647 | 0.5232 | 0.5232 | 0.4883 | 0.4534 | 0.4534 |

RSCU*, Relative synonymous codon usage; STOP*, Stop codon.
