# Supplementary material for: Molecular evolution of chloroplast genomes in subfamily Zingiberoideae (Zingiberaceae)
Source: BMC Plant Biol. 2021 Nov 23;21:558. doi: 10.1186/s12870-021-03315-9 (PMC8611967; doi:10.1186/s12870-021-03315-9)
Supplement: Supplementary file 6 — Additional file 6: Table S6. List of RNA editing sites in 10 assembled chloroplast genomes of subfamily Zingiberoideae as predicted by the PREP program [67]. [file 12870_2021_3315_MOESM6_ESM.docx]

**Table S6. List of RNA editing sites in ten assembled chloroplast genomes of subfamily Zingiberoideae predicted by PREP program [**59**].**

| Gene | A.A position | *G. lancangensis* | *G. marantina* | *G. multiflora* | *G. schomburgkii* | *G. schomburgkii* var. angustata | *H. coccineum* | *H. neocarneum* | *K. rotunda* ‘Red Leaf’ | *K. rotunda* ‘Silver Diamonds’ | *Z. recurvatum* |
| --- | --- | --- | --- | --- | --- | --- | --- | --- | --- | --- | --- |
|  |  | Codon (A.A) Conversion | | | | | | | | | |
| *accD* | 5 | CGG (R)≧TGG (W) | CGG (R) ≧TGG (W) | CGG (R)≧TGG (W) | CGG (R)≧TGG (W) | CGG (R)≧TGG (W) | CGG (R)≧TGG (W) | CGG (R)≧TGG (W) | CGG (R) ≧TGG (W) | CGG (R)≧TGG (W) | CGG (R)≧TGG (W) |
|  | 21 | CCA (P)≧CTA (L) | CCA (P) ≧CTA (L) | CCA (P)≧CTA (L) | CCA (P)≧CTA (L) | CCA (P)≧CTA (L) | CCA (P)≧CTA (L) | CCA (P)≧CTA (L) | CCG (P)≧CTG (L) | CCG (P)≧CTG (L) | CCG (P)≧CTG (L) |
|  | 52 | CGG (R)≧TGG (W) | CGG (R)≧TGG (W) | CGG (R)≧TGG (W) | - | CGG (R)≧TGG (W) | - | - | - | - | - |
|  | 131 | CAT (H)≧TAT (Y) | CAT (H)≧TAT (Y) | CAT (H)≧TAT (Y) | CAT (H)≧TAT (Y) | CAT (H)≧TAT (Y) | CAT (H)≧TAT (Y) | CAT (H)≧TAT (Y) | 133/CAT (H)≧TAT (Y) | 133/CAT (H)≧TAT (Y) | 133/CAT (H)≧TAT (Y) |
|  | 231 | TCA (S)≧TTA (L) | TCA (S)≧TTA (L) | TCA (S)≧TTA (L) | TCA (S)≧TTA (L) | TCA (S)≧TTA (L) | TCA (S)≧TTA (L) | TCA (S)≧TTA (L) | 233/TCA (S)≧TTA (L) | 233/TCA (S)≧TTA (L) | 233/TCA (S)≧TTA (L) |
|  | 262 | TCG (S)≧TTG (L) | TCG (S)≧TTG (L) | TCG (S)≧TTG (L) | TCG (S)≧TTG (L) | TCG (S)≧TTG (L) | TCG (S)≧TTG (L) | TCG (S)≧TTG (L) | 264/TCG (S)≧TTG (L) | 264/TCG (S)≧TTG (L) | 264/TCG (S)≧TTG (L) |
| *atpA* | 305 | TCA (S)≧TTA (L) | TCA (S)≧TTA (L) | TCA (S)≧TTA (L) | TCA (S)≧TTA (L) | - | TCA (S)≧TTA (L) | TCA (S)≧TTA (L) | TCA (S)≧TTA (L) | TCA (S)≧TTA (L) | TCA (S)≧TTA (L) |
| *atpB* | 132 | CCC (P)≧TCC (S) | CCC (P)≧TCC (S) | CCC (P)≧TCC (S) | CCC (P)≧TCC (S) | CCC (P)≧TCC (S) | CCC (P)≧TCC (S) | CCC (P)≧TCC (S) | CCC (P)≧TCC (S) | CCC (P)≧TCC (S) | CCC (P)≧TCC (S) |
|  | 392 | TCA (S)≧TTA (L) | TCA (S)≧TTA (L) | TCA (S)≧TTA (L) | TCA (S)≧TTA (L) | TCA (S)≧TTA (L) | TCA (S)≧TTA (L) | TCA (S)≧TTA (L) | TCA (S)≧TTA (L) | TCA (S)≧TTA (L) | TCA (S)≧TTA (L) |
| *atpF* | 31 | CCA (P)≧CTA (L) | CCA (P)≧CTA (L) | CCA (P)≧CTA (L) | CCA (P)≧CTA (L) | CCA (P)≧CTA (L) | CCA (P)≧CTA (L) | CCA (P)≧CTA (L) | CCA (P)≧CTA (L) | CCA (P)≧CTA (L) | CCA (P)≧CTA (L) |
| *aptI* | 210 | TCA (S)≧TTA (L) | TCA (S)≧TTA (L) | TCA (S)≧TTA (L) | TCA (S)≧TTA (L) | TCA (S)≧TTA (L) | TCA (S)≧TTA (L) | TCA (S)≧TTA (L) | TCA (S)≧TTA (L) | TCA (S)≧TTA (L) | TCA (S)≧TTA (L) |
| *ccsA* | 217 | ACT (T)≧ATT (I) | ACT (T)≧ATT (I) | ACT (T)≧ATT (I) | ACT (T)≧ATT (I) | ACT (T)≧ATT (I) | ACT (T)≧ATT (I) | ACT (T)≧ATT (I) | ACT (T)≧ATT (I) | ACT (T)≧ATT (I) | ACT (T)≧ATT (I) |
| *clpP* | 186 | CAC (H)≧TAC (Y) | CAC (H)≧TAC (Y) | CAC (H)≧TAC (Y) | 187/CAC (H)≧TAC (Y) | CAC (H)≧TAC (Y) | 185/ CAC (H)≧TAC (Y) | CAC (H)≧TAC (Y) | CAC (H)≧TAC (Y) | CAC (H)≧TAC (Y) | CAC (H)≧TAC (Y) |
| *matK* | 111 | - | - | - | - | - | - | - | ACT (T)≧ATT (I) | ACT (T)≧ATT (I) | - |
|  | 232 | - | CCT (P)≧CTT (L) | CCT (P)≧CTT (L) | CCT (P)≧CTT (L) | CCT (P)≧CTT (L) | CCT (P)≧CTT (L) | CCT (P)≧CTT (L) | CCT (P)≧CTT (L) | CCT (P)≧CTT (L) | CCT (P)≧CTT (L) |
|  | 305 | CAC (H)≧TAC (Y) | CAC (H)≧TAC (Y) | CAC (H)≧TAC (Y) | CAC (H)≧TAC (Y) | CAC (H)≧TAC (Y) | CAC (H)≧TAC (Y) | CAC (H)≧TAC (Y) | CAC (H)≧TAC (Y) | CAC (H)≧TAC (Y) | CAC (H)≧TAC (Y) |
|  | 421 | CAC (H)≧TAC (Y) | CAC (H)≧TAC (Y) | CAC (H)≧TAC (Y) | CAC (H)≧TAC (Y) | CAC (H)≧TAC (Y) | CAC (H)≧TAC (Y) | CAC (H)≧TAC (Y) | CAC (H)≧TAC (Y) | CAC (H)≧TAC (Y) | CAC (H)≧TAC (Y) |
|  | 453 | GCA (A)≧GTA (V) | GCA (A)≧GTA (V) | GCA (A)≧GTA (V) | GCA (A)≧GTA (V) | GCA (A)≧GTA (V) | GCA (A)≧GTA (V) | GCA (A)≧GTA (V) | GCA (A)≧GTA (V) | GCA (A)≧GTA (V) | GCA (A)≧GTA (V) |
| *ndhA* | 17 | TCG (S)≧TTG (L) | TCG (S)≧TTG (L) | TCG (S)≧TTG (L) | - | TCG (S)≧TTG (L) | TCG (S)≧TTG (L) | TCG (S)≧TTG (L) | TCG (S)≧TTG (L) | TCG (S)≧TTG (L) | TCG (S)≧TTG (L) |
|  | 23 | - | - | - | - | - | - | - | - | - | GCC (A)≧GTC (V) |
|  | 24 | CAT (H)≧TAT (Y) | CAT (H)≧TAT (Y) | CAT (H)≧TAT (Y) | - | CAT (H)≧TAT (Y) | CAT (H)≧TAT (Y) | CAT (H)≧TAT (Y) | CAT (H)≧TAT (Y) | CAT (H)≧TAT (Y) | CAT (H)≧TAT (Y) |
|  | 132 | - | TCT (S)≧TTT (F) | - | - | TCT (S)≧TTT (F) | TCT (S)≧TTT (F) | TCT (S)≧TTT (F) | TCT (S)≧TTT (F) | TCT (S)≧TTT (F) | - |
|  | 159 | TCA (S)≧TTA (L) | TCA (S)≧TTA (L) | TCA (S)≧TTA (L) | - | TCA (S)≧TTA (L) | TCA (S)≧TTA (L) | TCA (S)≧TTA (L) | TCA (S)≧TTA (L) | TCA (S)≧TTA (L) | TCA (S)≧TTA (L) |
|  | 189 | TCA (S)≧TTA (L) | TCA (S)≧TTA (L) | TCA (S)≧TTA (L) | - | TCA (S)≧TTA (L) | TCA (S)≧TTA (L) | TCA (S)≧TTA (L) | TCA (S)≧TTA (L) | TCA (S)≧TTA (L) | TCA (S)≧TTA (L) |
|  | 308 | CTT (L)≧TTT (F) | CTT (L)≧TTT (F) | CTT (L)≧TTT (F) | - | CTT (L)≧TTT (F) | CTT (L)≧TTT (F) | CTT (L)≧TTT (F) | CTT (L)≧TTT (F) | CTT (L)≧TTT (F) | CTT (L)≧TTT (F) |
|  | 358 | TCT (S)≧TTT (F) | TCT (S)≧TTT (F) | TCT (S)≧TTT (F) | - | TCT (S)≧TTT (F) | TCG (S)≧TTG (L) | TCG (S)≧TTG (L) | TCG (S)≧TTG (L) | TCG (S)≧TTG (L) | TCT (S)≧TTT (F) |

**Table S6. Continued.**

| Gene | A.A position | *G. lancangensis* | *G. marantina* | *G. multiflora* | *G. schomburgkii* | *G. schomburgkii* var. angustata | *H. coccineum* | *H. neocarneum* | *K. rotunda* ‘Red Leaf’ | *K. rotunda* ‘Silver Diamonds’ | *Z. recurvatum* |
| --- | --- | --- | --- | --- | --- | --- | --- | --- | --- | --- | --- |
|  |  | Codon (A.A) Conversion | | | | | | | | | |
| *ndhB* | 50 | 32/TCA (S)≧TTA (L) | TCA (S)≧TTA (L) | TCA (S)≧TTA (L) | TCA (S)≧TTA (L) | TCA (S)≧TTA (L) | TCA (S)≧TTA (L) | TCA (S)≧TTA (L) | TCA (S)≧TTA (L) | TCA (S)≧TTA (L) | TCA (S)≧TTA (L) |
|  | 156 | 138/TCA (S)≧TTA (L) | TCA (S)≧TTA (L) | TCA (S)≧TTA (L) | TCA (S)≧TTA (L) | TCA (S)≧TTA (L) | TCA (S)≧TTA (L) | TCA (S)≧TTA (L) | TCA (S)≧TTA (L) | TCA (S)≧TTA (L) | TCA (S)≧TTA (L) |
|  | 181 | 163/ACG (T)≧ATG (M) | ACG (T ≧ATG (M) | ACG (T)≧ATG (M) | - | ACG (T)≧ATG (M) | ACG (T)≧ATG (M) | ACG (T)≧ATG (M) | - | - | ACG (T)≧ATG (M) |
|  | 204 | 186/TCA (S)≧TTA (L) | TCA (S)≧TTA (L) | TCA (S)≧TTA (L) | TCA (S)≧TTA (L) | TCA (S)≧TTA (L) | TCA (S)≧TTA (L) | TCA (S)≧TTA (L) | TCA (S)≧TTA (L) | TCA (S)≧TTA (L) | TCA (S)≧TTA (L) |
|  | 235 | 217/TCC (S)≧TTC (F) | TCC (S)≧TTC (F) | TCC (S)≧TTC (F) | TCC (S)≧TTC (F) | TCC (S)≧TTC (F) | TCC (S)≧TTC (F) | TCC (S)≧TTC (F) | TCC (S)≧TTC (F) | TCC (S)≧TTC (F) | TCC (S)≧TTC (F) |
|  | 246 | 228/CCA (P)≧CTA (L) | CCA (P)≧CTA (L) | CCA (P)≧CTA (L) | CCA (P)≧CTA (L) | - | CCA (P)≧CTA (L) | CCA (P)≧CTA (L) | CCA (P)≧CTA (L) | CCA (P)≧CTA (L) | CCA (P)≧CTA (L) |
|  | 277 | 259/TCA (S)≧TTA (L) | TCA (S)≧TTA (L) | TCA (S)≧TTA (L) | 288/TCA (S)≧TTA (L) | TCA (S)≧TTA (L) | TCA (S)≧TTA (L) | TCA (S)≧TTA (L) | TCA (S)≧TTA (L) | TCA (S)≧TTA (L) | TCA (S)≧TTA (L) |
|  | 279 | 261/TCA (S)≧TTA (L) | TCA (S)≧TTA (L) | TCA (S)≧TTA (L) | - | TCA (S)≧TTA (L) | TCA (S)≧TTA (L) | TCA (S)≧TTA (L) | TCA (S)≧TTA (L) | TCA (S)≧TTA (L) | TCA (S)≧TTA (L) |
|  | 398 | 380/TCA (S)≧TTA (L) | TCA (S)≧TTA (L) | TCA (S)≧TTA (L) | - | TCA (S)≧TTA (L) | TCA (S)≧TTA (L) | TCA (S)≧TTA (L) | TCA (S)≧TTA (L) | TCA (S)≧TTA (L) | TCA (S)≧TTA (L) |
|  | 419 | 401/CAT (H)≧TAT (Y) | CAT (H)≧TAT (Y) | CAT (H)≧TAT (Y) | 428/CAT (H)≧TAT (Y) | CAT (H)≧TAT (Y) | CAT (H)≧TAT (Y) | CAT (H)≧TAT (Y) | CAT (H)≧TAT (Y) | CAT (H)≧TAT (Y) | CAT (H)≧TAT (Y) |
|  | 494 | 476/CCA (P)≧CTA (L) | CCA (P)≧CTA (L) | CCA (P)≧CTA (L) | 503/CCA (P)≧CTA (L) | CCA (P)≧CTA (L) | CCA (P)≧CTA (L) | CCA (P)≧CTA (L) | CCA (P)≧CTA (L) | CCA (P)≧CTA (L) | CCA (P)≧CTA (L) |
| *ndhD* | 2 | ACG (T)≧ATG (M) | ACG (T)≧ATG (M) | ACG (T)≧ATG (M) | - | ACG (T)≧ATG (M) | ACG (T)≧ATG (M) | ACG (T)≧ATG (M) | ACG (T)≧ATG (M) | ACG (T)≧ATG (M) | ACG (T)≧ATG (M) |
|  | 17 | - | - | - | - | - | - | - | - | - | TCC (S)≧TTC (F) |
|  | 50 | CAC (H)≧TAC (Y) | CAC (H)≧TAC (Y) | CAC (H)≧TAC (Y) | - | CAC (H)≧TAC (Y) | CAC (H)≧TAC (Y) | CAC (H)≧TAC (Y) | CAC (H)≧TAC (Y) | CAC (H)≧TAC (Y) | CAC (H)≧TAC (Y) |
|  | 54 | CAT (H)≧TAT (Y) | CAT (H)≧TAT (Y) | CAT (H)≧TAT (Y) | - | CAT (H)≧TAT (Y) | CAT (H)≧TAT (Y) | CAT (H)≧TAT (Y) | CAT (H)≧TAT (Y) | CAT (H)≧TAT (Y) | CAT (H)≧TAT (Y) |
|  | 129 | CCA (P)≧CTA (L) | CCA (P)≧CTA (L) | CCA (P)≧CTA (L) | - | CCA (P)≧CTA (L) | CCA (P)≧CTA (L) | CCA (P)≧CTA (L) | CCA (P)≧CTA (L) | CCA (P)≧CTA (L) | CCA (P)≧CTA (L) |
|  | 294 | TCG (S)≧TTG (L) | TCG (S)≧TTG (L) | TCG (S)≧TTG (L) | - | TCG (S)≧TTG (L) | TCG (S)≧TTG (L) | TCG (S)≧TTG (L) | TCG (S)≧TTG (L) | TCG (S)≧TTG (L) | TCG (S)≧TTG (L) |
|  | 317 | ACA (T)≧ATA (I) | ACA (T)≧ATA (I) | ACA (T)≧ATA (I) | - | ACA (T)≧ATA (I) | ACA (T)≧ATA (I) | ACA (T)≧ATA (I) | ACA (T)≧ATA (I) | ACA (T)≧ATA (I) | ACA (T)≧ATA (I) |
|  | 399 | TCA (S)≧TTA (L) | TCA (S)≧TTA (L) | TCA (S)≧TTA (L) | - | TCA (S)≧TTA (L) | TCA (S)≧TTA (L) | TCA (S)≧TTA (L) | TCA (S)≧TTA (L) | TCA (S)≧TTA (L) | TCA (S)≧TTA (L) |
|  | 476 | ACT (T)≧ATT (I) | ACT (T)≧ATT (I) | ACT (T)≧ATT (I) | - | ACT (T)≧ATT (I) | ACT (T)≧ATT (I) | ACT (T)≧ATT (I) | ACT (T)≧ATT (I) | ACT (T)≧ATT (I) | ACT (T)≧ATT (I) |
| *ndhF* | 21 | TCA (S)≧TTA (L) | TCA (S)≧TTA (L) | TCA (S)≧TTA (L) | - | TCA (S)≧TTA (L) | TCA (S)≧TTA (L) | TCA (S)≧TTA (L) | TCA (S)≧TTA (L) | TCA (S)≧TTA (L) | TCA (S)≧TTA (L) |
|  | 97 | TCA (S)≧TTA (L) | TCA (S)≧TTA (L) | TCA (S)≧TTA (L) | - | TCA (S)≧TTA (L) | TCA (S)≧TTA (L) | TCA (S)≧TTA (L) | TCA (S)≧TTA (L) | TCA (S)≧TTA (L) | TCA (S)≧TTA (L) |
|  | 224 | TCA (S)≧TTA (L) | TCA (S)≧TTA (L) | TCA (S)≧TTA (L) | - | TCA (S)≧TTA (L) | TCA (S)≧TTA (L) | TCA (S)≧TTA (L) | TCA (S)≧TTA (L) | TCA (S)≧TTA (L) | TCA (S)≧TTA (L) |
|  | 474 | CAT (H)≧TAT (Y) | CAT (H)≧TAT (Y) | CAT (H)≧TAT (Y) | - | CAT (H)≧TAT (Y) | CAT (H)≧TAT (Y) | CAT (H)≧TAT (Y) | CAT (H)≧TAT (Y) | CAT (H)≧TAT (Y) | CAT (H)≧TAT (Y) |
|  | 579 | ACA (T)≧ATA (I) | ACA (T)≧ATA (I) | ACA (T)≧ATA (I) | - | ACA (T)≧ATA (I) | ACA (T)≧ATA (I) | ACA (T)≧ATA (I) | ACA (T)≧ATA (I) | ACA (T)≧ATA (I) | - |
|  | 724 | - | GCA (A)≧GTA (V) | GCA (A)≧GTA (V) | - | GCA (A)≧GTA (V) | GCA (A)≧GTA (V) | GCA (A)≧GTA (V) | GCA (A)≧GTA (V) | GCA (A)≧GTA (V) | 738/GCA (A)≧GTA (V) |

**Table S6. Continued.**

| Gene | A.A position | *G. lancangensis* | *G. marantina* | *G. multiflora* | *G. schomburgkii* | *G. schomburgkii* var. *angustata* | *H. coccineum* | *H. neocarneum* | *K. rotunda* ‘Red Leaf’ | *K. rotunda* ‘Silver Diamonds’ | *Z. recurvatum* |
| --- | --- | --- | --- | --- | --- | --- | --- | --- | --- | --- | --- |
|  |  | Codon (A.A) Conversion | | | | | | | | | |
| *ndhG* | 7 | ACA (T)≧ATA (I) | ACA (T)≧ATA (I) | ACA (T)≧ATA (I) | - | ACA (T)≧ATA (I) | ACA (T)≧ATA (I) | ACA (T)≧ATA (I) | ACA (T)≧ATA (I) | ACA (T)≧ATA (I) | ACA (T)≧ATA (I) |
|  | 52 | TCA (S)≧TTA (L) | TCA (S)≧TTA (L) | - | - | TCA (S)≧TTA (L) | TCA (S)≧TTA (L) | TCA (S)≧TTA (L) | TCA (S)≧TTA (L) | TCA (S)≧TTA (L) | TCA (S)≧TTA (L) |
| *petB* | 140 | CGG (R)≧TGG (W) | CGG (R)≧TGG (W) | CGG (R)≧TGG (W) | 142/CGG (R)≧TGG (W) | CGG (R)≧TGG (W) | CGG (R)≧TGG (W) | CGG (R)≧TGG (W) | CGG (R)≧TGG (W) | CGG (R)≧TGG (W) | CGG (R)≧TGG (W) |
|  | 204 | CCA (P)≧CTA (L) | CCA (P)≧CTA (L) | CCA (P)≧CTA (L) | 206/CCA (P)≧CTA (L) | CCA (P)≧CTA (L) | CCA (P)≧CTA (L) | CCA (P)≧CTA (L) | CCA (P)≧CTA (L) | CCA (P)≧CTA (L) | CCA (P)≧CTA (L) |
| *psaB* | 20 | - | - | - | CTT (L)≧TTT (F) | - | - | - | - | - | - |
|  | 110 | - | - | - | GCA (A)≧GTA (V) | - | - | - | - | - | - |
|  | 164 | - | - | - | CCT (P)≧TCT (S) | - | - | - | - | - | - |
|  | 192 | - | - | - | CCT (P)≧CTT (L) | - | - | - | - | - | - |
|  | 262 | - | - | - | ACA (T)≧ATA (I) | - | - | - | - | - | - |
|  | 306 | - | - | - | CCC (P)≧CTC (L) | - | - | - | - | - | - |
|  | 344 | - | - | - | CCT (P)≧CTT (L) | - | - | - | - | - | - |
|  | 388 | - | - | - | TCA (S)≧TTA (L) | - | - | - | - | - | - |
| *psbB* | 51 | GCT (A)≧GTT (V) | GCT (A)≧GTT (V) | GCT (A)≧GTT (V) | - | GCT (A)≧GTT (V) | GCT (A)≧GTT (V) | GCT (A)≧GTT (V) | GCT (A)≧GTT (V) | GCT (A)≧GTT (V) | GCT (A)≧GTT (V) |
| *psbF* | 26 | TCT (S)≧TTT (F) | TCT (S)≧TTT (F) | TCT (S)≧TTT (F) | TCT (S)≧TTT (F) | TCT (S)≧TTT (F) | TCT (S)≧TTT (F) | TCT (S)≧TTT (F) | TCT (S)≧TTT (F) | TCT (S)≧TTT (F) | TCT (S)≧TTT (F) |
| *rpl2* | 1 | - | - | - | ACG (T)≧ATG (M) | - | - | - | - | - | - |
| *rpl20* | 44 | GCT (A)≧GTT (V) | GCT (A)≧GTT (V) | GCT (A)≧GTT (V) | GCT (A)≧GTT (V) | GCT (A)≧GTT (V) | GCT (A)≧GTT (V) | GCT (A)≧GTT (V) | GCT (A)≧GTT (V) | GCT (A)≧GTT (V) | GCT (A)≧GTT (V) |
|  | 97 | CCT (P)≧CTT (L) | CCT (P)≧CTT (L) | CCT (P)≧CTT (L) | CCT (P)≧CTT (L) | CCT (P)≧CTT (L) | CCT (P)≧CTT (L) | CCT (P)≧CTT (L) | CCT (P)≧CTT (L) | CCT (P)≧CTT (L) | CCT (P)≧CTT (L) |
|  | 103 | TCA (S)≧TTA (L) | TCA (S)≧TTA (L) | TCA (S)≧TTA (L) | TCA (S)≧TTA (L) | TCA (S)≧TTA (L) | TCA (S)≧TTA (L) | TCA (S)≧TTA (L) | TCA (S)≧TTA (L) | TCA (S)≧TTA (L) | TCA (S)≧TTA (L) |
| *rpoA* | 101 | CAT (H)≧TAT (Y) | CAT (H)≧TAT (Y) | CAT (H)≧TAT (Y) | CAT (H)≧TAT (Y) | CAT (H)≧TAT (Y) | CAT (H)≧TAT (Y) | CAT (H)≧TAT (Y) | CAT (H)≧TAT (Y) | CAT (H)≧TAT (Y) | CAT (H)≧TAT (Y) |
|  | 123 | TCA (S)≧TTA (L) | TCA (S)≧TTA (L) | TCA (S)≧TTA (L) | TCA (S)≧TTA (L) | TCA (S)≧TTA (L) | TCA (S)≧TTA (L) | TCA (S)≧TTA (L) | TCA (S)≧TTA (L) | TCA (S)≧TTA (L) | TCA (S)≧TTA (L) |
|  | 279 | TCA (S)≧TTA (L) | 277/TCA (S)≧TTA (L) | TCA (S)≧TTA (L) | TCA (S)≧TTA (L) | TCA (S)≧TTA (L) | TCA (S)≧TTA (L) | TCA (S)≧TTA (L) | TCA (S)≧TTA (L) | TCA (S)≧TTA (L) | TCA (S)≧TTA (L) |
| *rpoB* | 159 | TCA (S)≧TTA (L) | TCA (S)≧TTA (L) | TCA (S)≧TTA (L) | TCA (S)≧TTA (L) | TCA (S)≧TTA (L) | TCA (S)≧TTA (L) | TCA (S)≧TTA (L) | TCA (S)≧TTA (L) | TCA (S)≧TTA (L) | TCA (S)≧TTA (L) |
|  | 185 | TCG (S)≧TTG (L) | TCG (S)≧TTG (L) | TCG (S)≧TTG (L) | TCG (S)≧TTG (L) | TCG (S)≧TTG (L) | TCG (S)≧TTG (L) | TCG (S)≧TTG (L) | TCG (S)≧TTG (L) | TCG (S)≧TTG (L) | TCG (S)≧TTG (L) |
|  | 190 | TCG (S)≧TTG (L) | TCG (S)≧TTG (L) | TCG (S)≧TTG (L) | TCG (S)≧TTG (L) | TCG (S)≧TTG (L) | TCG (S)≧TTG (L) | TCG (S)≧TTG (L) | TCG (S)≧TTG (L) | TCG (S)≧TTG (L) | TCG (S)≧TTG (L) |
|  | 450 | - | - | - | CGG (R)≧TGG (W) | - | - | - | - | - | - |
|  | 823 | TCA (S)≧TTA (L) | TCA (S)≧TTA (L) | TCA (S)≧TTA (L) | 814/TCA (S)≧TTA (L) | TCA (S)≧TTA (L) | 821/TCA (S)≧TTA (L) | 821/TCA (S)≧TTA (L) | TCA (S)≧TTA (L) | TCA (S)≧TTA (L) | 814/TCA (S)≧TTA (L) |

**Table S6. Continued.**

| Gene | A.A position | *G. lancangensis* | *G. marantina* | *G. multiflora* | *G. schomburgkii* | *G. schomburgkii* var. *angustata* | *H. coccineum* | *H. neocarneum* | *K. rotunda* ‘Red Leaf’ | *K. rotunda* ‘Silver Diamonds’ | *Z. recurvatum* |
| --- | --- | --- | --- | --- | --- | --- | --- | --- | --- | --- | --- |
|  |  | Codon (A.A) Conversion | | | | | | | | | |
| *rpoC1* | 61 | TCT (S)≧TTT (F) | TCT (S)≧TTT (F) | TCT (S)≧TTT (F) | TCT (S)≧TTT (F) | TCT (S)≧TTT (F) | TCT (S)≧TTT (F) | TCT (S)≧TTT (F) | TCT (S)≧TTT (F) | TCT (S)≧TTT (F) | TCT (S)≧TTT (F) |
|  | 209 | TCA (S)≧TTA (L) | TCA (S)≧TTA (L) | TCA (S)≧TTA (L) | 206/TCA (S)≧TTA (L) | TCA (S)≧TTA (L) | TCA (S)≧TTA (L) | TCA (S)≧TTA (L) | /TCA (S)≧TTA (L) | TCA (S)≧TTA (L) | TCA (S)≧TTA (L) |
| *rpoC2* | 661 | - | - | - | - | - | - | - | CTT (L)≧TTT (F) | CTT (L)≧TTT (F) | 7/CCG (P)≧CTG (L) |
|  | 763 | CGG (R)≧TGG (W) | 755/CGG (R)≧TGG (W) | CGG (R)≧TGG (W) | CGG (R)≧TGG (W) | 755/CGG (R)≧TGG (W) | 761/CGG (R)≧TGG (W) | 761/CGG (R)≧TGG (W) | 761/CGG (R)≧TGG (W) | 761/CGG (R)≧TGG (W) | 761/CGG (R)≧TGG (W) |
|  | 774 | TCG (S)≧TTG (L) | 766/TCG (S)≧TTG (L) | TCG (S)≧TTG (L) | TCG (S)≧TTG (L) | 766/TCG (S)≧TTG (L) | 772/TCG (S)≧TTG (L) | 772/TCG (S)≧TTG (L) | 772/TCG (S)≧TTG (L) | 772/TCG (S)≧TTG (L) | 772/TCG (S)≧TTG (L) |
|  | 1237 | TCA (S)≧TTA (L) | 1229/TCA (S)≧TTA (L) | TCA (S)≧TTA (L) | TCA (S)≧TTA (L) | 1229/TCA (S)≧TTA (L) | 1234/TCA (S)≧TTA (L) | 1234/TCA (S)≧TTA (L) | 1174/CGG (R)≧TGG (W) | 1174/CGG (R)≧TGG (W) | 1238/TCA (S)≧TTA (L) |
|  | 1367 | - | - | CCA (P)≧TCA (S) | - | - | 1364/CCA (P)≧TCA (S) | 1364/CCA (P)≧TCA (S) | 1234/TCA (S)≧TTA (L) | 1234/TCA (S)≧TTA (L) | - |
| *rps2* | 83 | TCA (S)≧TTA (L) | TCA (S)≧TTA (L) | TCA (S)≧TTA (L) | TCA (S)≧TTA (L) | TCA (S)≧TTA (L) | - | - | TCA (S)≧TTA (L) | TCA (S)≧TTA (L) | TCA (S)≧TTA (L) |
| *rps8* | 73 | CAT (H)≧TAT (Y) | CAT (H)≧TAT (Y) | CAT (H)≧TAT (Y) | CAT (H)≧TAT (Y) | CAT (H)≧TAT (Y) | 75/CAT (H)≧TAT (Y) | 75/CAT (H)≧TAT (Y) | CAT (H)≧TAT (Y) | CAT (H)≧TAT (Y) | CAT (H)≧TAT (Y) |
| *rps14* | 27 | TCA (S)≧TTA (L) | TCA (S)≧TTA (L) | TCA (S)≧TTA (L) | TCA (S)≧TTA (L) | TCA (S)≧TTA (L) | TCA (S)≧TTA (L) | TCA (S)≧TTA (L) | TCA (S)≧TTA (L) | TCA (S)≧TTA (L) | TCA (S)≧TTA (L) |
|  | 50 | CCA (P)≧CTA (L) | CCA (P)≧CTA (L) | CCA (P)≧CTA (L) | CCA (P)≧CTA (L) | CCA (P)≧CTA (L) | CCA (P)≧CTA (L) | CCA (P)≧CTA (L) | CCA (P)≧CTA (L) | CCA (P)≧CTA (L) | CCA (P)≧CTA (L) |
|  | 73 | CAT (H)≧TAT (Y) | CAT (H)≧TAT (Y) | CAT (H)≧TAT (Y) | CAT (H)≧TAT (Y) | CAT (H)≧TAT (Y) | CAT (H)≧TAT (Y) | CAT (H)≧TAT (Y) | CAT (H)≧TAT (Y) | CAT (H)≧TAT (Y) | CAT (H)≧TAT (Y) |
| *rps16* | 48 | TCA (S)≧TTA (L) | TCA (S)≧TTA (L) | TCA (S)≧TTA (L) | - | TCA (S)≧TTA (L) | TCA (S)≧TTA (L) | TCA (S)≧TTA (L) | TCA (S)≧TTA (L) | TCA (S)≧TTA (L) | TCA (S)≧TTA (L) |
| *ycf3* | 15 | - | TCT (S)≧TTT (F) | TCT (S)≧TTT (F) | TCT (S)≧TTT (F) | TCT (S)≧TTT (F) | TCT (S)≧TTT (F) | TCT (S)≧TTT (F) | TCT (S)≧TTT (F) | TCT (S)≧TTT (F) | TCT (S)≧TTT (F) |
|  | 64 | ACG (T)≧ATG (M) | ACG (T)≧ATG (M) | ACG (T)≧ATG (M) | 55/ACG (T)≧ATG (M) | ACG (T)≧ATG (M) | ACG (T)≧ATG (M) | ACG (T)≧ATG (M) | ACG (T)≧ATG (M) | ACG (T)≧ATG (M) | ACG (T)≧ATG (M) |
|  | 66 | CCA (P)≧CTA (L) | CCA (P)≧CTA (L) | CCA (P)≧CTA (L) | 57/CCA (P)≧CTA (L) | CCA (P)≧CTA (L) | CCA (P)≧CTA (L) | CCA (P)≧CTA (L) | CCA (P)≧CTA (L) | CCA (P)≧CTA (L) | CCA (P)≧CTA (L) |
|  | 138 | TCC (S)≧TTC (F) | TCC (S)≧TTC (F) | TCC (S)≧TTC (F) | TCC (S)≧TTC (F) | TCC (S)≧TTC (F) | TCC (S)≧TTC (F) | TCC (S)≧TTC (F) | TCC (S)≧TTC (F) | TCC (S)≧TTC (F) | TCC (S)≧TTC (F) |
