# Supplementary material for: Molecular evolution of chloroplast genomes in subfamily Zingiberoideae (Zingiberaceae)
Source: BMC Plant Biol. 2021 Nov 23;21:558. doi: 10.1186/s12870-021-03315-9 (PMC8611967; doi:10.1186/s12870-021-03315-9)
Supplement: Supplementary file 13 — Additional file 13: Table S13. The 59 chloroplast genomes used for phylogenetic analysis. [file 12870_2021_3315_MOESM13_ESM.docx]

**Table S13. The 59 chloroplast genomes using in phylogenetic analysis.**

| Taxa | Genbank  number | Taxa | Genbank number |
| --- | --- | --- | --- |
| *Amomum compactum* | NC_036992 | *Curcuma phaeocaulis* Yunnan | MK621772 |
| *Amomum longiligulare _Y19019* | MN067434 | *Curcuma roscoeana* | KF601574 |
| *Amomum longiligulare _Y19020* | MN067435 | *Curcuma roscoeana isolate jinbian_uang C11* | MT395652 |
| *Alpinia katsumadai* | MK262728 | *Curcuma roscoeana isolate yinbian_uangC12* | MT395654 |
| *Amomum kravanh* | MF991963 | *Curcuma sichuanensis isolate chuanyujin C6* | MT395644 |
| *Alpinia oxyphylla* Hainan | NC_035895 | *Curcuma wenyujin isolate wenyujin C8* | MT395653 |
| *Alpinia oxyphylla* Guangdong | MK262729 | *Curcuma xanthorrhiza isolate yinnieshuC1* | MT395655 |
| *Alpinia pumila* | MK262731 | *Curcuma xanthorrhiza isolate yinnieshuC14* | MT395656 |
| *Amomum villosa_Y17085* | MH161416 | *Curcuma yunnanensis isolate dinghuaeshu C3* | MT395646 |
| *Amomum villosa_Y17089* | MH161418 | *Curcuma zedoaria* | MK262734 |
| *Amomum villosa_Y19017* | MN067431 | *Globba lancangensis* | MT473704 |
| *Amomum villosa_Y19021* | MN067432 | *Globba marantina* | MT473705 |
| *Amomum villosa var. xanthioides_Y17088* | MH161417 | *Globba multiflora* | MT473706 |
| *Amomum villosa var. xanthioides_Y19018* | MN067433 | *Globba schomburgkii* | MK262735 |
| *Amomum villosum* | MK262730 | *Globba schomburgkii* *var. angustata* | MT473707 |
| *Alpinia zerumbet* | JX088668 | *Hedychium coccineum* | MT473708 |
| *Canna indica* | KF601570 | *Hedychium coronarium* | MK262736 |
| *Costus pulverulentus* | KF601573 | *Hedychium neocarneum* | MT473709 |
| *Costus viridis* | MK262733 | *Hedychium spicatum* | NC_047248 |
| *Curcuma alismatifolia isolate jianghehua C4* | MT395649 | *Kaempferia galanga* | MK209001 |
| *Curcuma amarissima isolate jikujia_huang C5* | MT395651 | *Kaempferia elegans* | MK209002 |
| *Curcuma aromatica isolate yujunC7* | MT395657 | *Kaempferia rotunda* ‘Red Leaf’ | MT473710 |
| *Curcuma aromatic* Yunnan | MK621773 | *Kaempferia rotunda* ‘Silver Diamonds’ | MT473711 |
| *Curcuma flaviflora* | KR967361 | *Stahlianthus involucratus* | MK262725 |
| *Curcuma elata isolate daeshu C2* | MT395645 | *Zingiber montanum* | MK262727 |
| *Curcuma kwangsiensis* Yunnan | MK621775 | *Zingiber officinale* | NC_044775 |
| *Curcuma longa* | MK262732 | *Zingiber recurvatum* | MT473712 |
| *Curcuma longa isolate jianghuang C10* | MT395650 | *Zingiber spectabile* | JX088661 |
| *Curcuma longa* Yunnan | MK621774 | *Zingier zerumbet* | MK262726 |
| *Curcuma phaeocaulis isolate eshu C13* | MT395647 |  |  |
