# Supplementary material for: Molecular evolution of chloroplast genomes in subfamily Zingiberoideae (Zingiberaceae)
Source: BMC Plant Biol. 2021 Nov 23;21:558. doi: 10.1186/s12870-021-03315-9 (PMC8611967; doi:10.1186/s12870-021-03315-9)
Supplement: Supplementary file 15 — Additional file 15: Figure S1. Gene maps of the other 9 assembled Zingiberoideae chloroplast genomes in this study. Genes shown inside the circle are transcribed clockwise, and those outside are transcribed counterclockwise. The gray arrowheads indicate the direction of the genes. Different genes are color coded. The innermost darker gray corresponds to GC content, whereas the lighter gray corresponds to AT content. The inner circle also indicates that the chloroplast genome contains a large single copy region (LSC), a small single copy region (SSC) and two copies of the inverted repeat (IRA and IRB). a G. marantina. b G. multiflora. c G. schomburgkii.* indicates the two sites of the two genes trnS-GCU and trnT-UGU only present in G. schomburgkii instead of the two genes trnS-GGA and trnT-GGU, respectively. d G. schomburgkii var. angustata. e H. coccineum.* indicates the site of the gene psbZ present in H. coccineum instead of the gene lhbA. f H. neocarneum. * indicates the site of the gene psbZ present in H. neocarneum instead of the gene lhbA. g K. rotunda ‘Red Leaf’. h K. rotunda ‘Silver Diamonds’. i Z. recurvatum. [file 12870_2021_3315_MOESM15_ESM.docx]

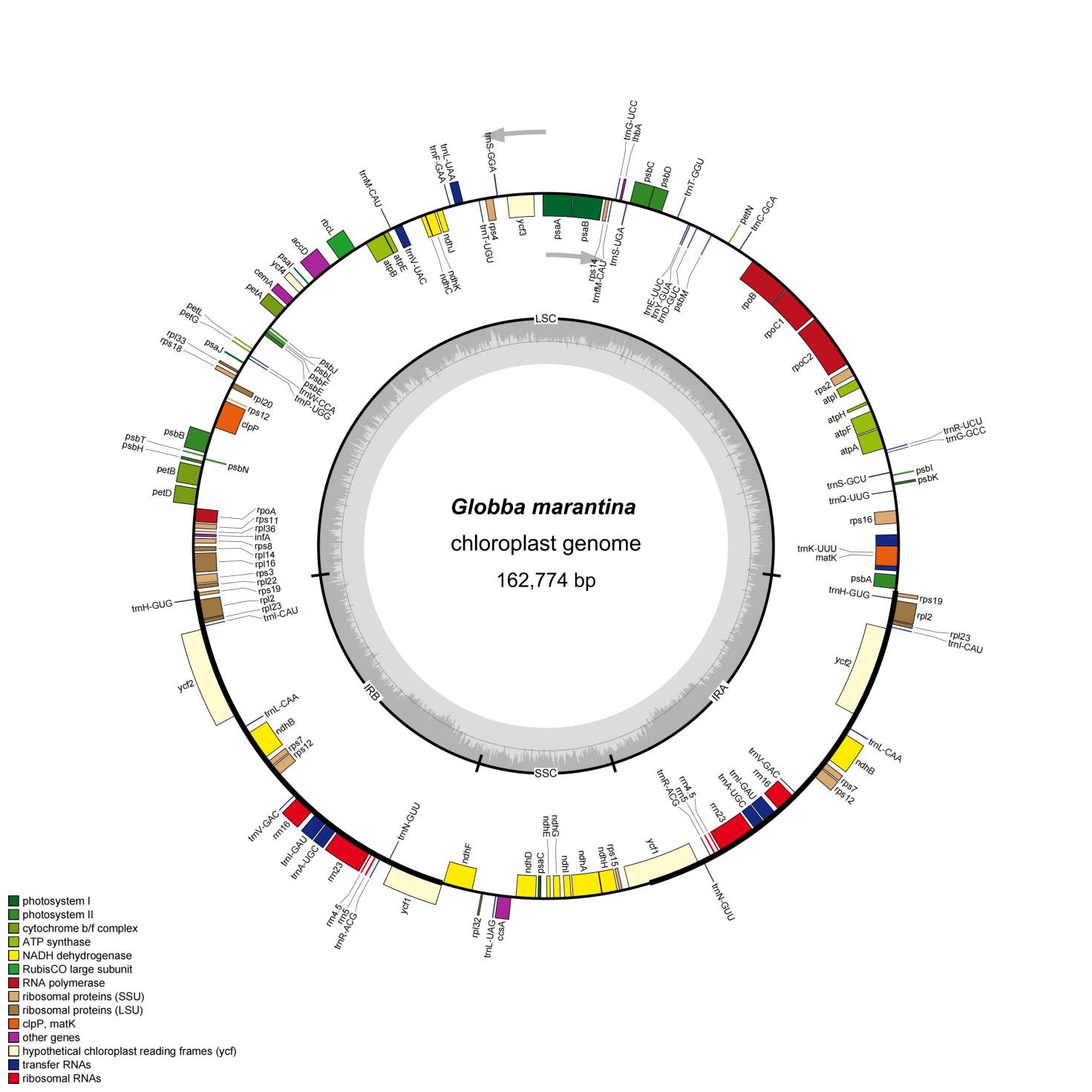


**a**

**Figure S1.** Gene maps of the other 9 assembled Zingiberoideae chloroplast genomes in this study. Genes shown inside the circle are transcribed clockwise, and those outside are transcribed counterclockwise. The gray arrowheads indicate the direction of the genes. Different genes are color coded. The innermost darker gray corresponds to GC content, whereas the lighter gray corresponds to AT content. The inner circle also indicates that the chloroplast genome contains a large single copy region (LSC), a small single copy region (SSC) and two copies of the inverted repeat (IRA and IRB). **a** *G. marantina.* **b** *G. multiflora.* **c** *G. schomburgkii.******** indicates the two sites of the two genes *trnS-GCU* and *trnT-UGU* only present in *G. schomburgkii* instead of the two genes *trnS-GGA* and *trnT-GGU*, respectively. **d** *G. schomburgkii* *var. angustata.* **e** *H. coccineum.******** indicates the site of the gene *psbZ* present in *H. coccineum* instead of the gene *lhbA.* **f** *H. neocarneum.* ******* indicates the site of the gene *psbZ* present in *H. neocarneum* instead of the gene *lhbA.* **g** *K. rotunda* ‘Red Leaf’. **h** *K. rotunda* ‘Silver Diamonds’. **i** *Z. recurvatum.*


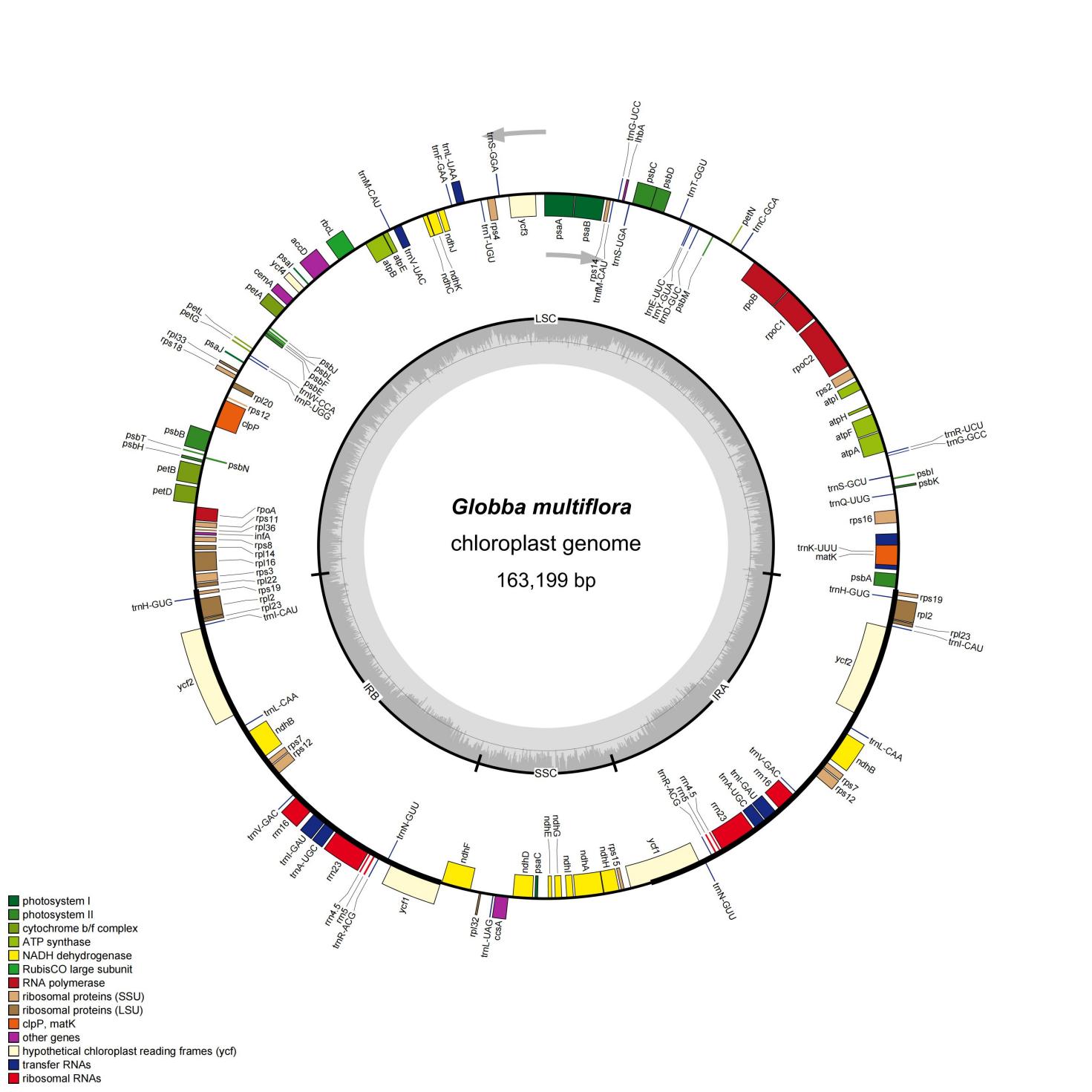


**b**

**Figure S1.** continued.


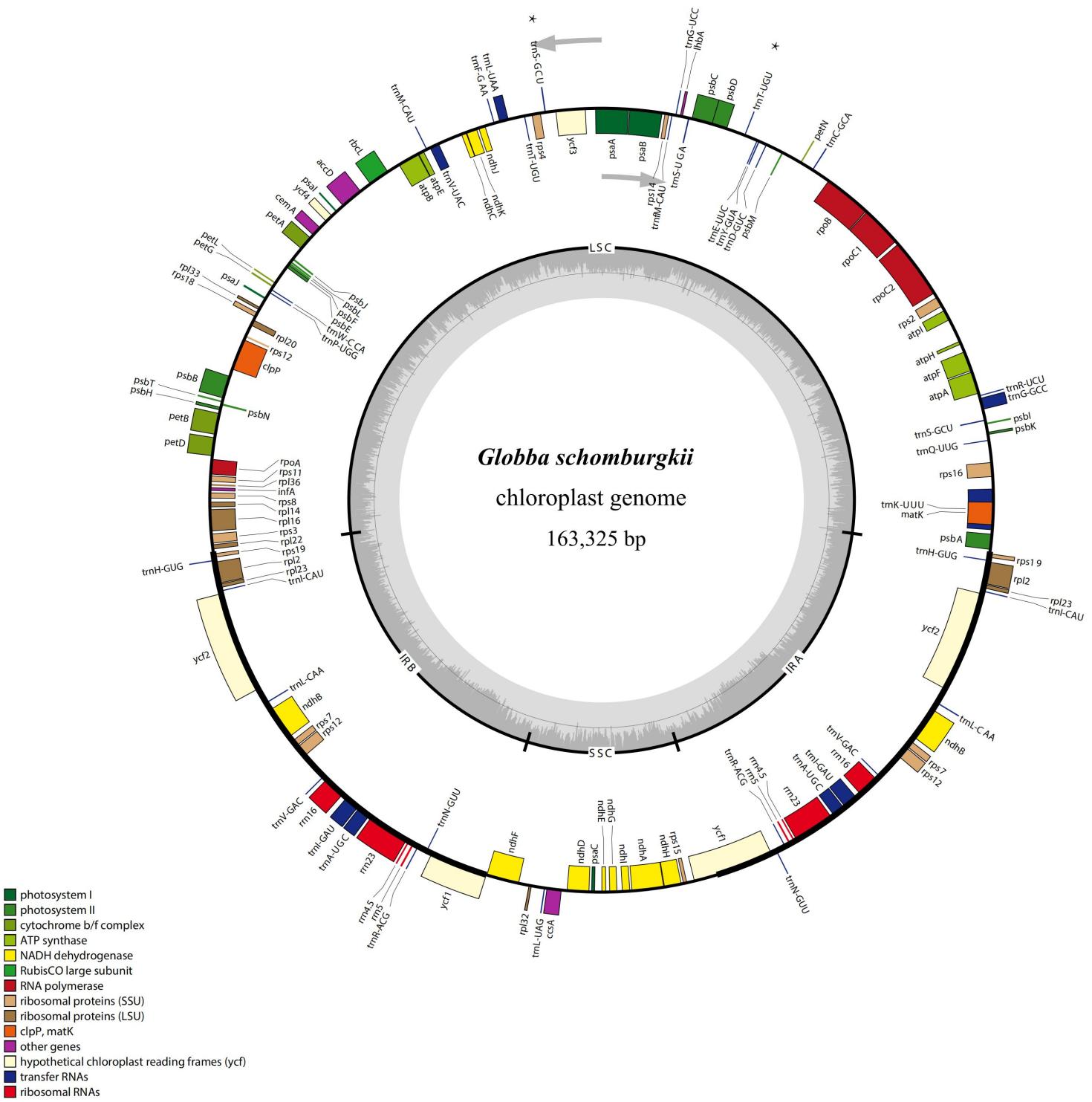


**c**

**Figure S1.** continued.


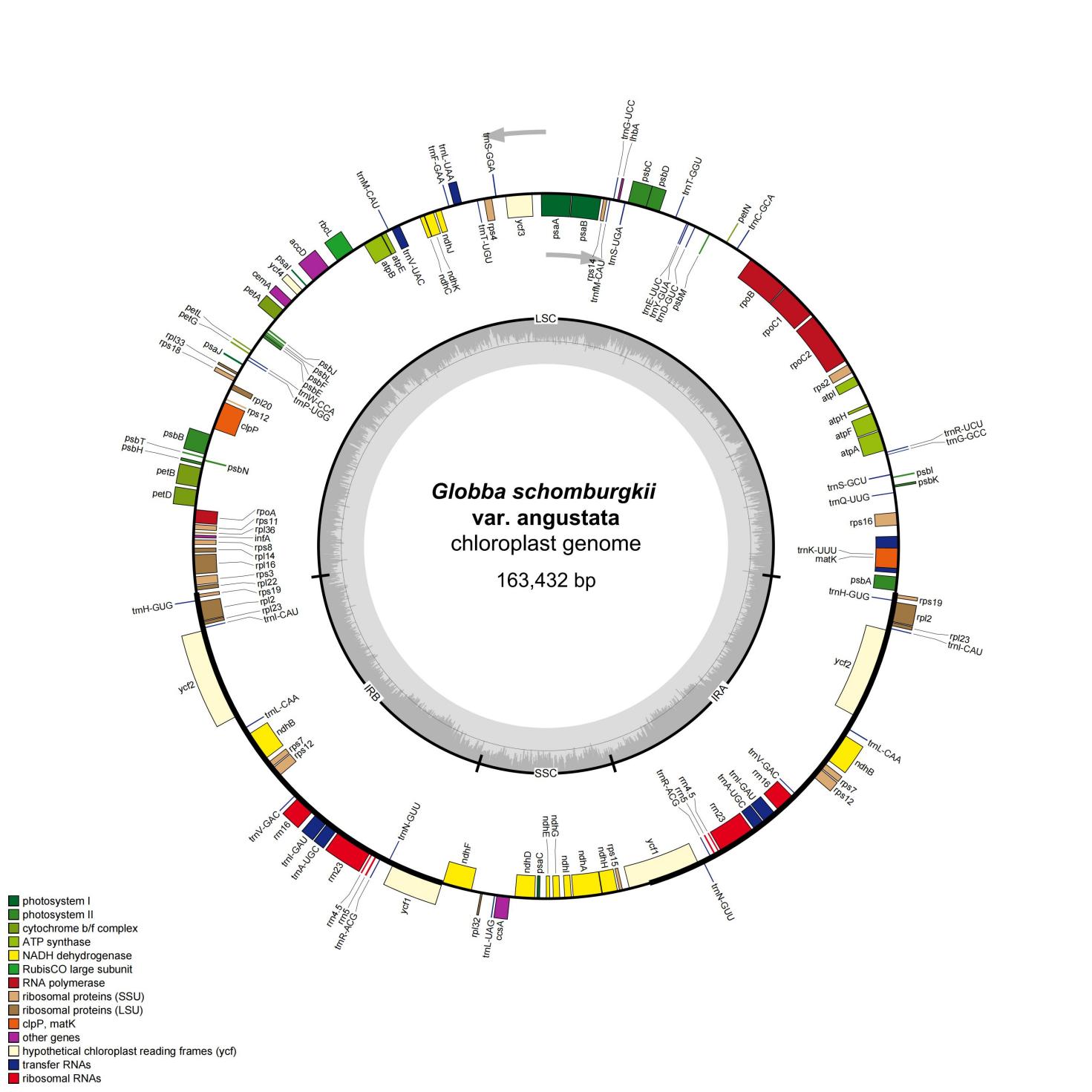


**d**

**Figure S1.** continued.


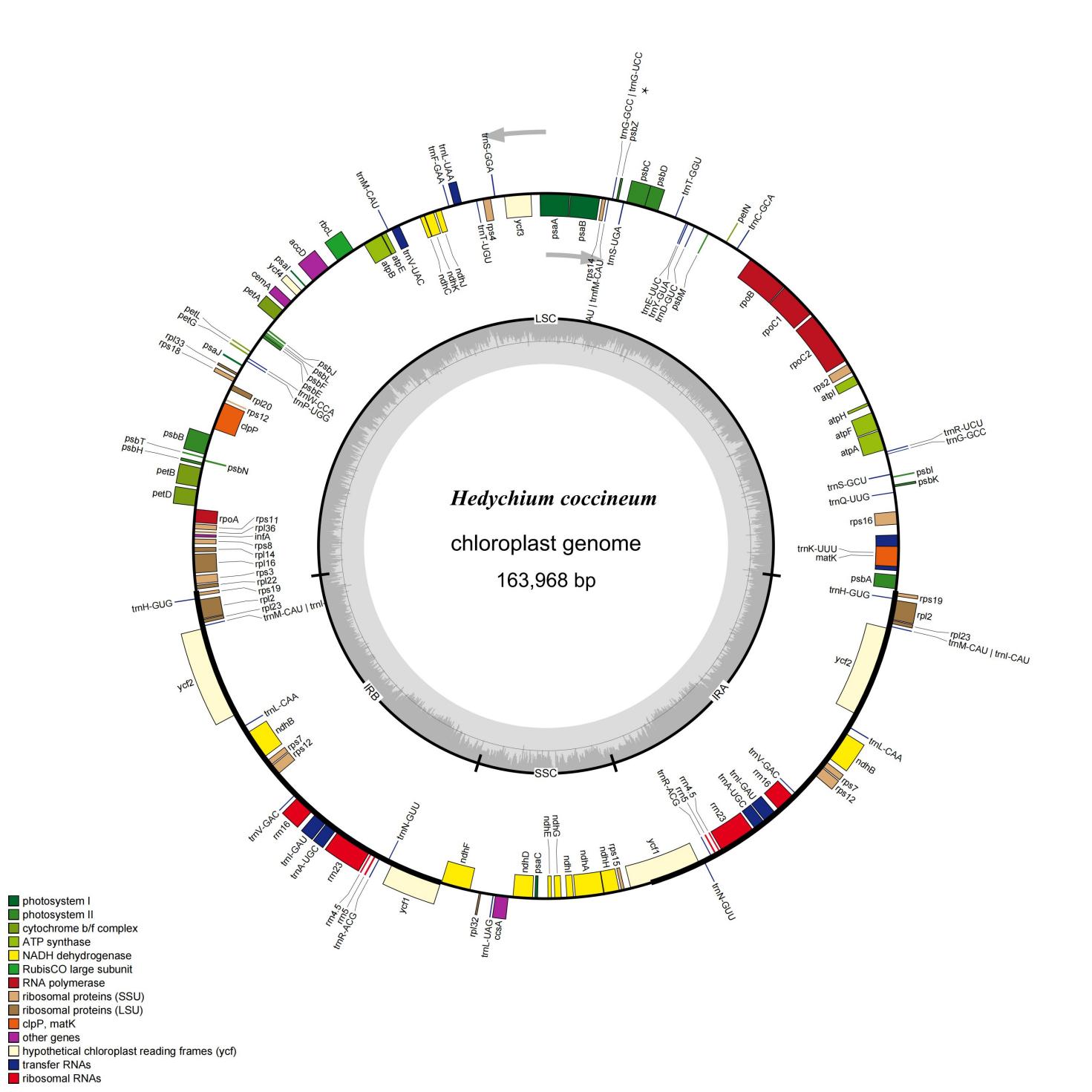


**e**

**Figure S1.** continued.


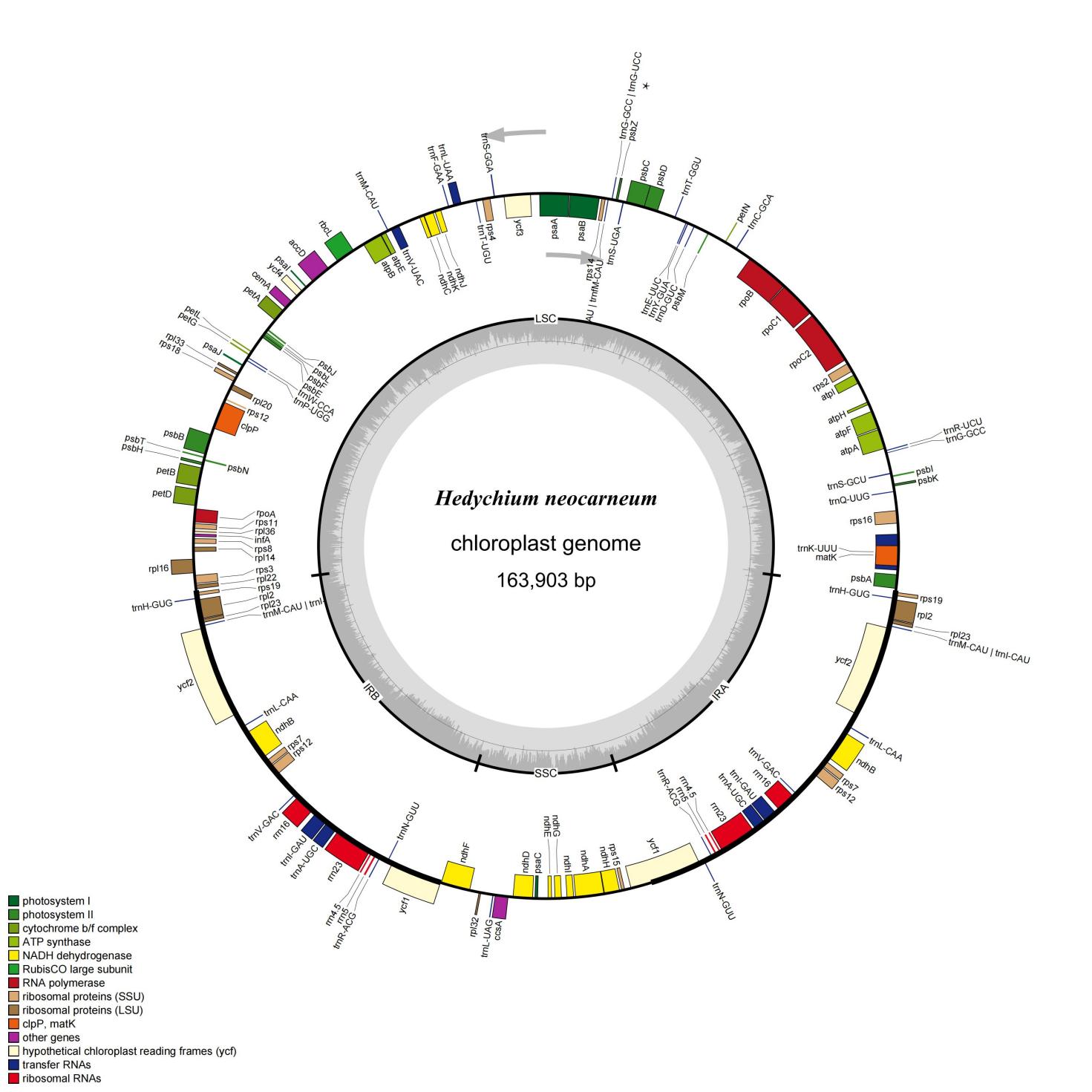


**f**

**Figure S1.** continued.


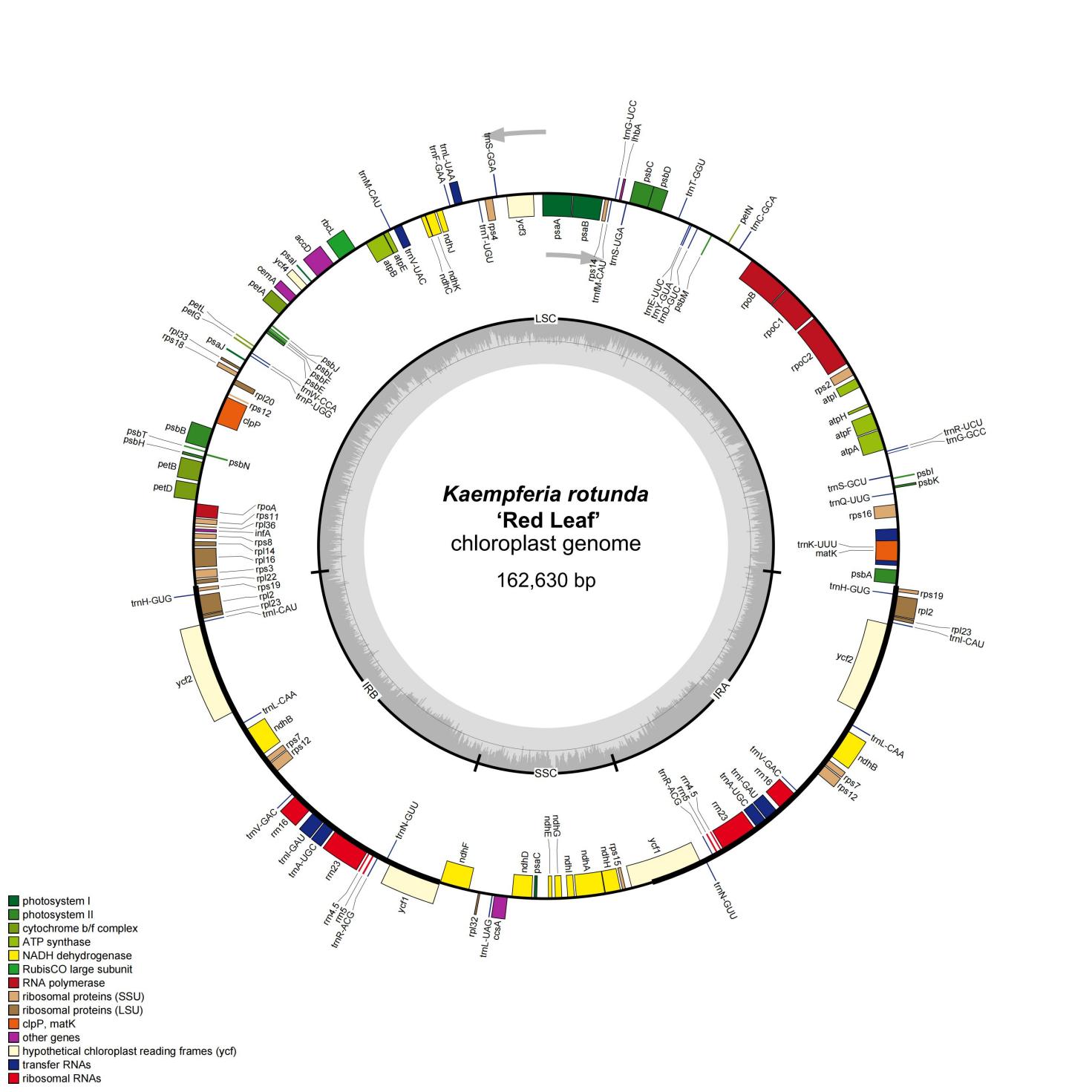


**g**

**Figure S1.** continued.


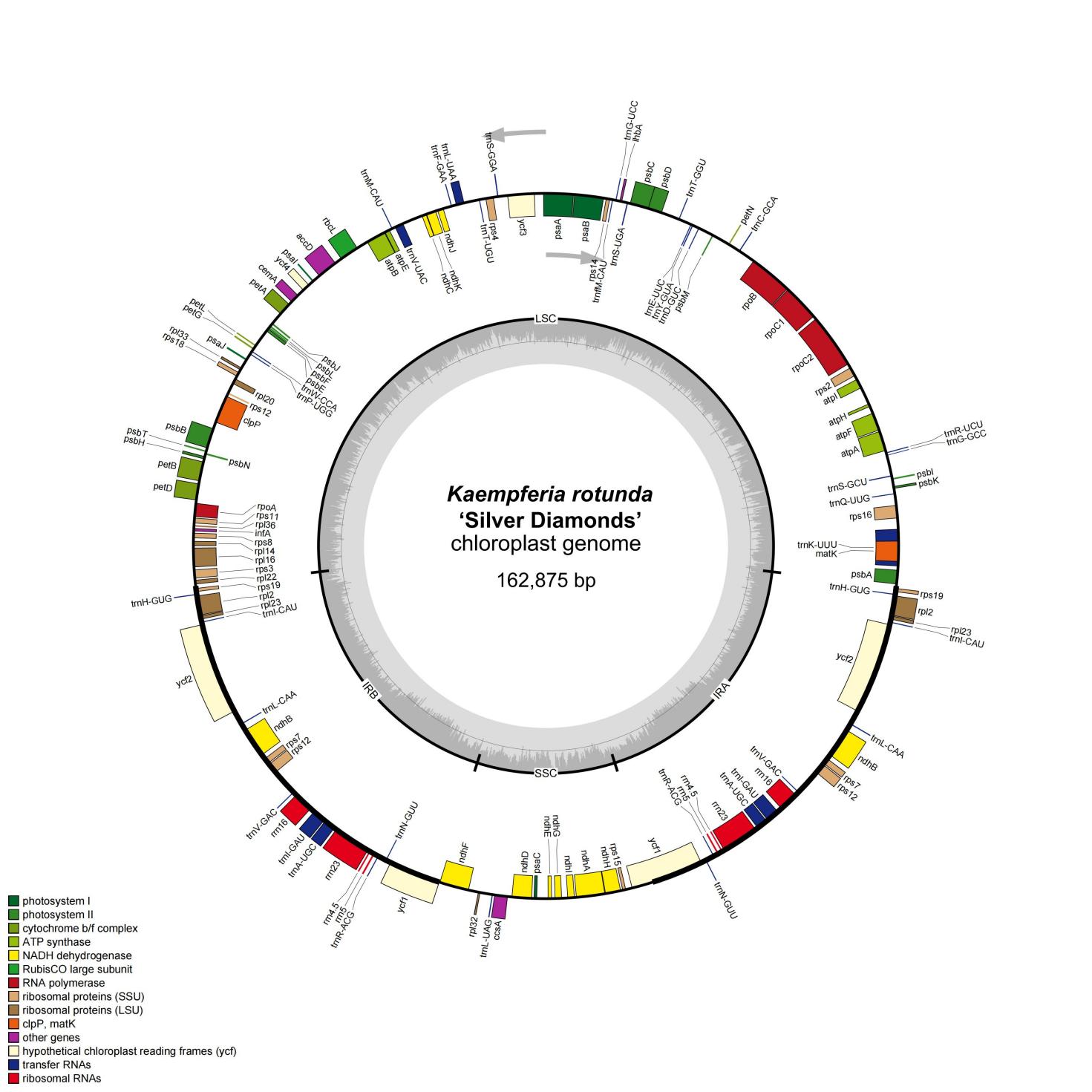


**h**

**Figure S1.** continued.


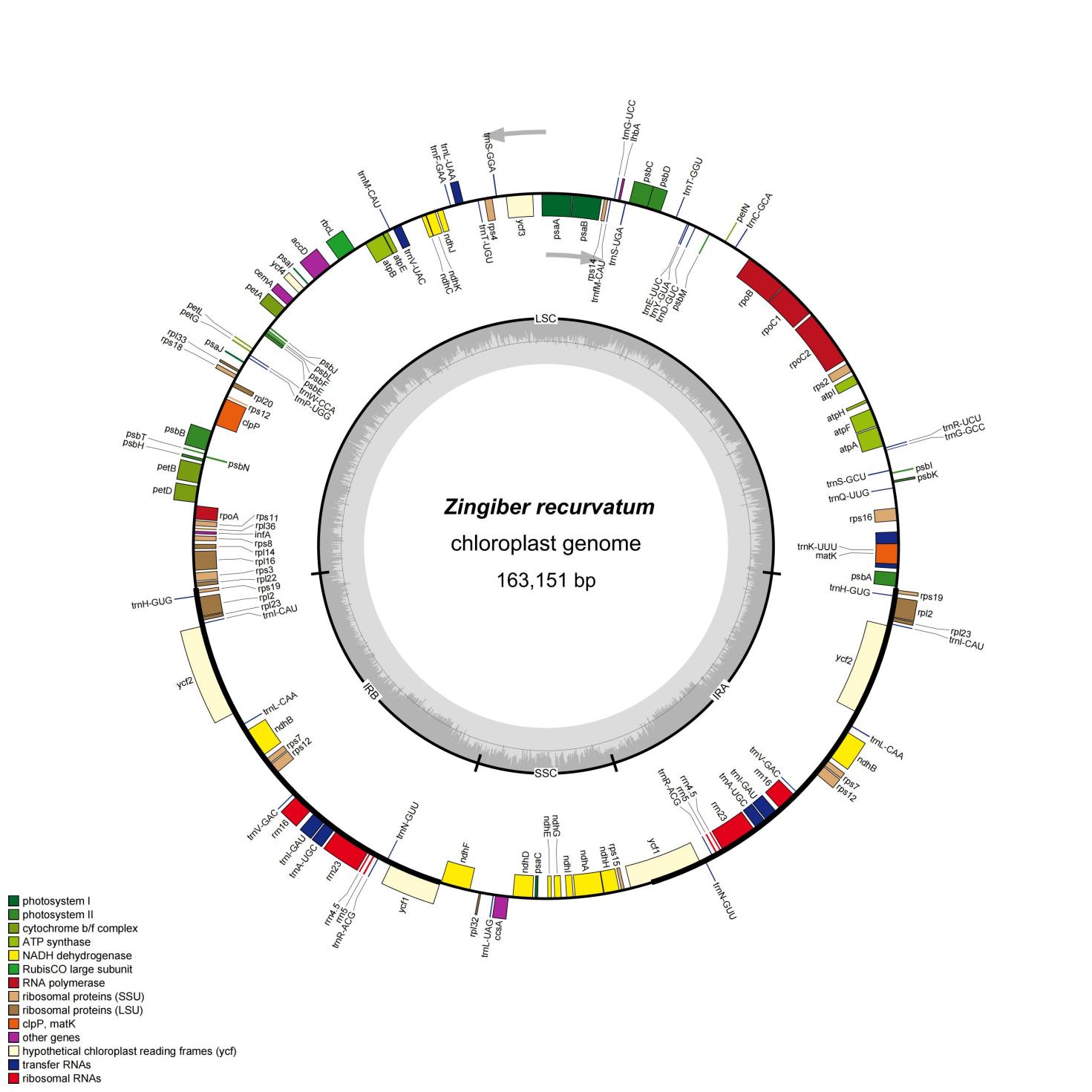


**i**

**Figure S1.** continued.
