# Supplementary material for: Molecular evolution of chloroplast genomes in subfamily Zingiberoideae (Zingiberaceae)
Source: BMC Plant Biol. 2021 Nov 23;21:558. doi: 10.1186/s12870-021-03315-9 (PMC8611967; doi:10.1186/s12870-021-03315-9)
Supplement: Supplementary file 16 — Additional file 16: Figure S2. Maximum likelihood (ML) trees of five Globba species, four Hedychium species, three Kaempferia species and five Zingiber species based on the chloroplast genomes divergent genes and intergenic regions. a ML tree based on the sequences of gene matK. b ML tree based on the sequences of gene ndhF. c ML tree based on the sequences of gene ycf1. d ML tree based on the intergenic sequences of trnK-UUU-CDS1-rps16-CDS2. e ML tree based on the intergenic sequences of psaJ-rpl33. f ML tree based on the intergenic sequences of ycf4-cemA. g ML tree based on the intergenic sequences of trnT-UGU-trnL-UAA-CDS1. h ML tree based on the intergenic sequences of trnT-GGU-psbD. i ML tree based on the intergenic sequences of rpl32-trnL-UAG. j ML tree based on the intergenic sequences of psbM-trnD-GUC. k ML tree based on the intergenic sequences of ndhF-rpl32. l ML tree based on the intergenic sequences of rps15-ycf1. m ML tree based on the intergenic sequences of ccsA-ndhD. [file 12870_2021_3315_MOESM16_ESM.docx]

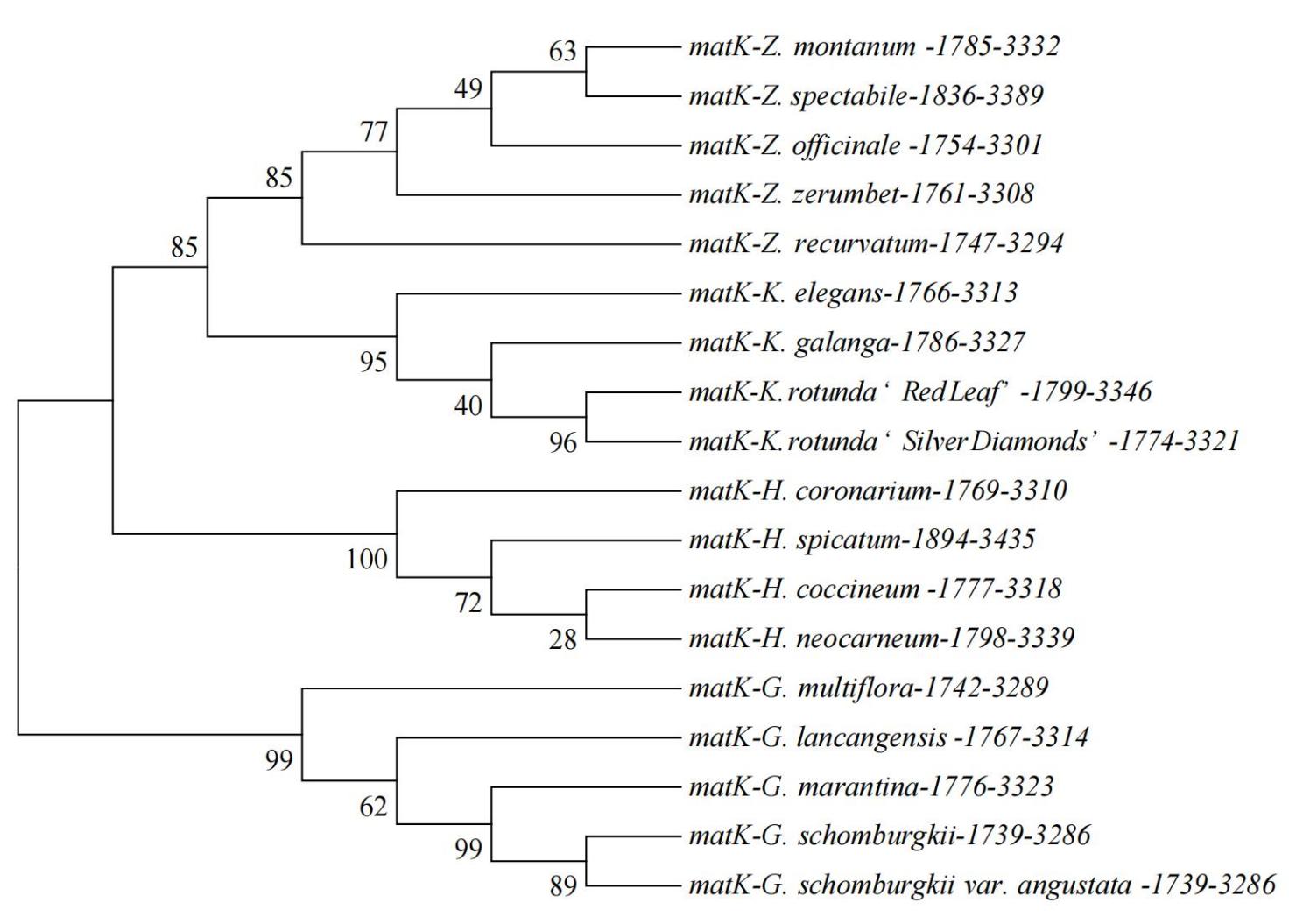


**a**

**Figure S2.** Maximum likelihood (ML) trees of five *Globba* species, four *Hedychium* species, three *Kaempferia* species and five *Zingiber* species based on the chloroplast genomes divergent genes and intergenic regions. **a** ML tree based on the sequences of gene *matK*. **b** ML tree based on the sequences of gene *ndhF*. **c** ML tree based on the sequences of gene *ycf1*. **d** ML tree based on the intergenic sequences of *trnK-UUU-CDS1*-*rps16-CDS2*. **e** ML tree based on the intergenic sequences of *psaJ*-*rpl33*. **f** ML tree based on the intergenic sequences of *ycf4-cemA*. **g** ML tree based on the intergenic sequences of *trnT-UGU-trnL-UAA-CDS1*. **h** ML tree based on the intergenic sequences of *trnT-GGU-psbD*. **i** ML tree based on the intergenic sequences of *rpl32*-*trnL-UAG*. **j** ML tree based on the intergenic sequences of *psbM-trnD-GUC*. **k** ML tree based on the intergenic sequences of *ndhF-rpl32*. **l** ML tree based on the intergenic sequences of *rps15-ycf1*. **m** ML tree based on the intergenic sequences of *ccsA-ndhD*.


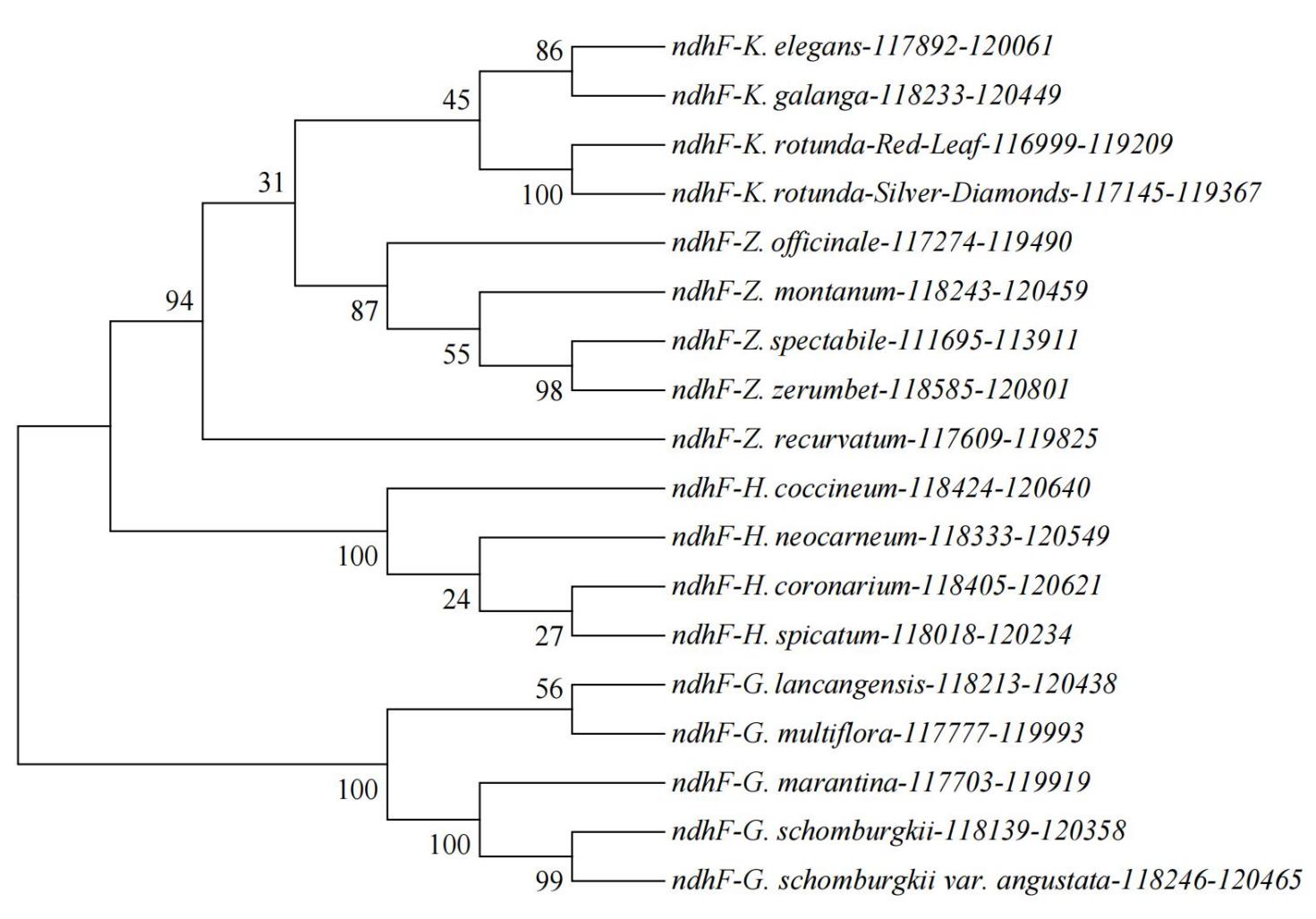


**b**


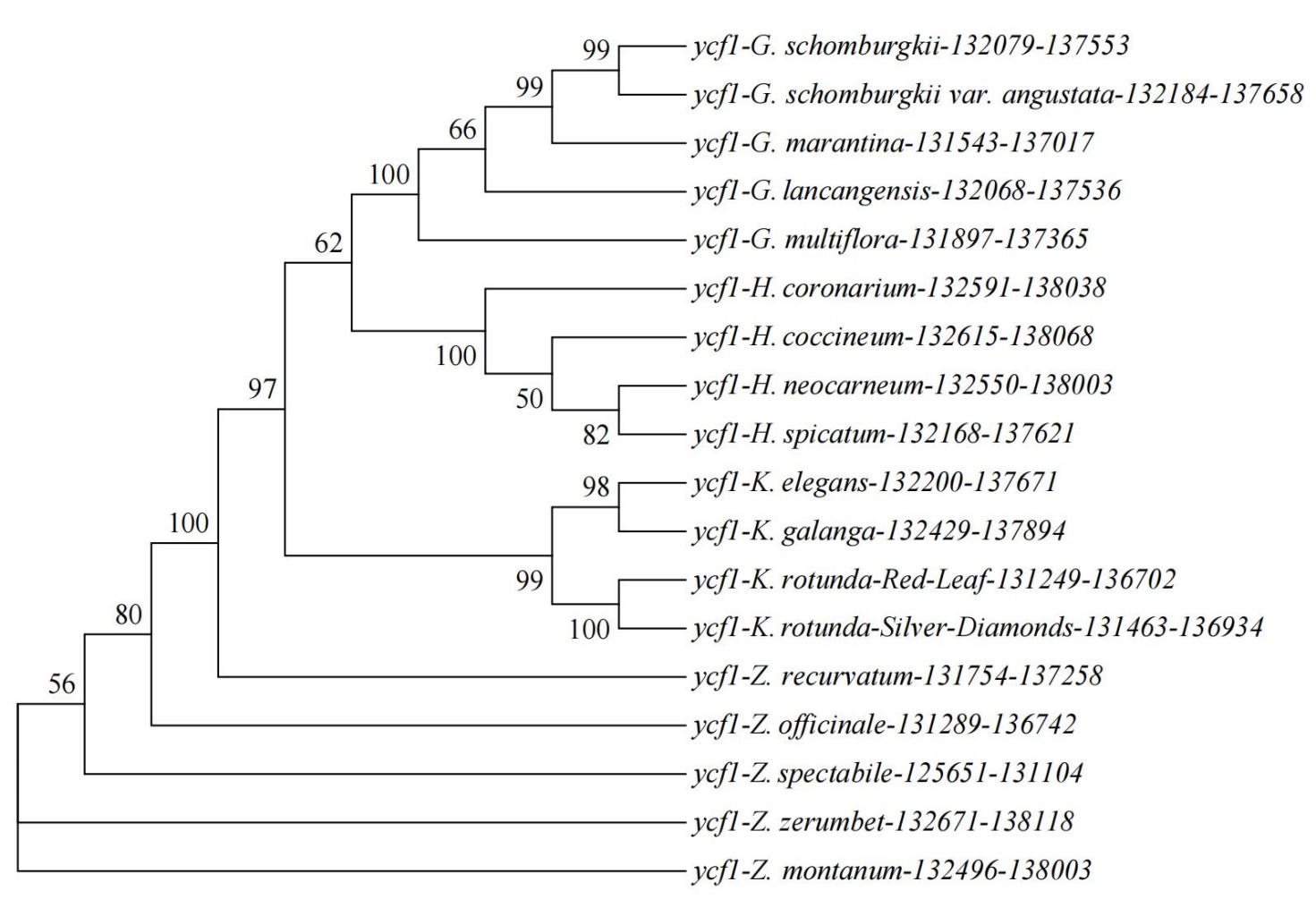


**c**

**Figure S2.** continued.


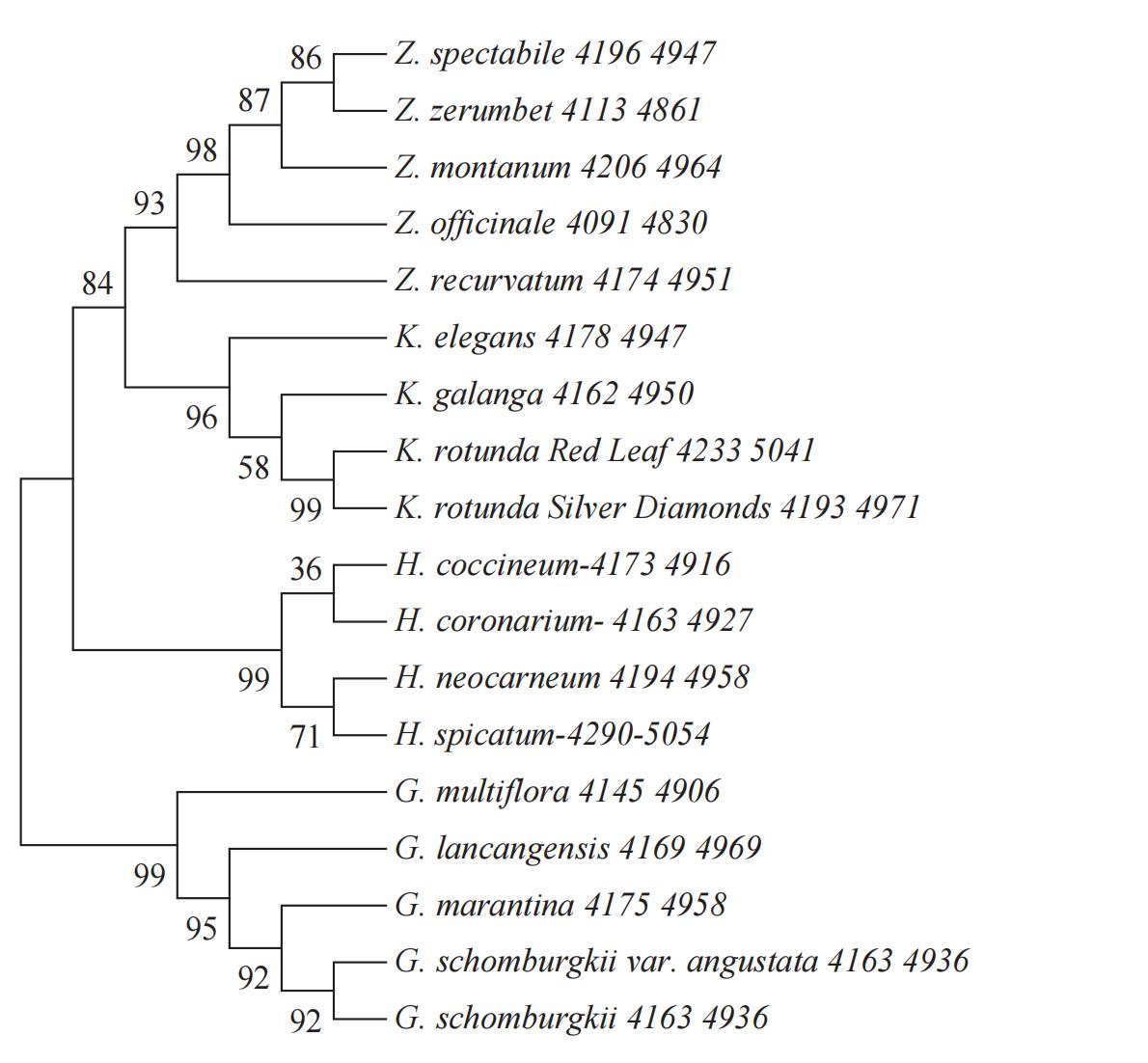


**d**


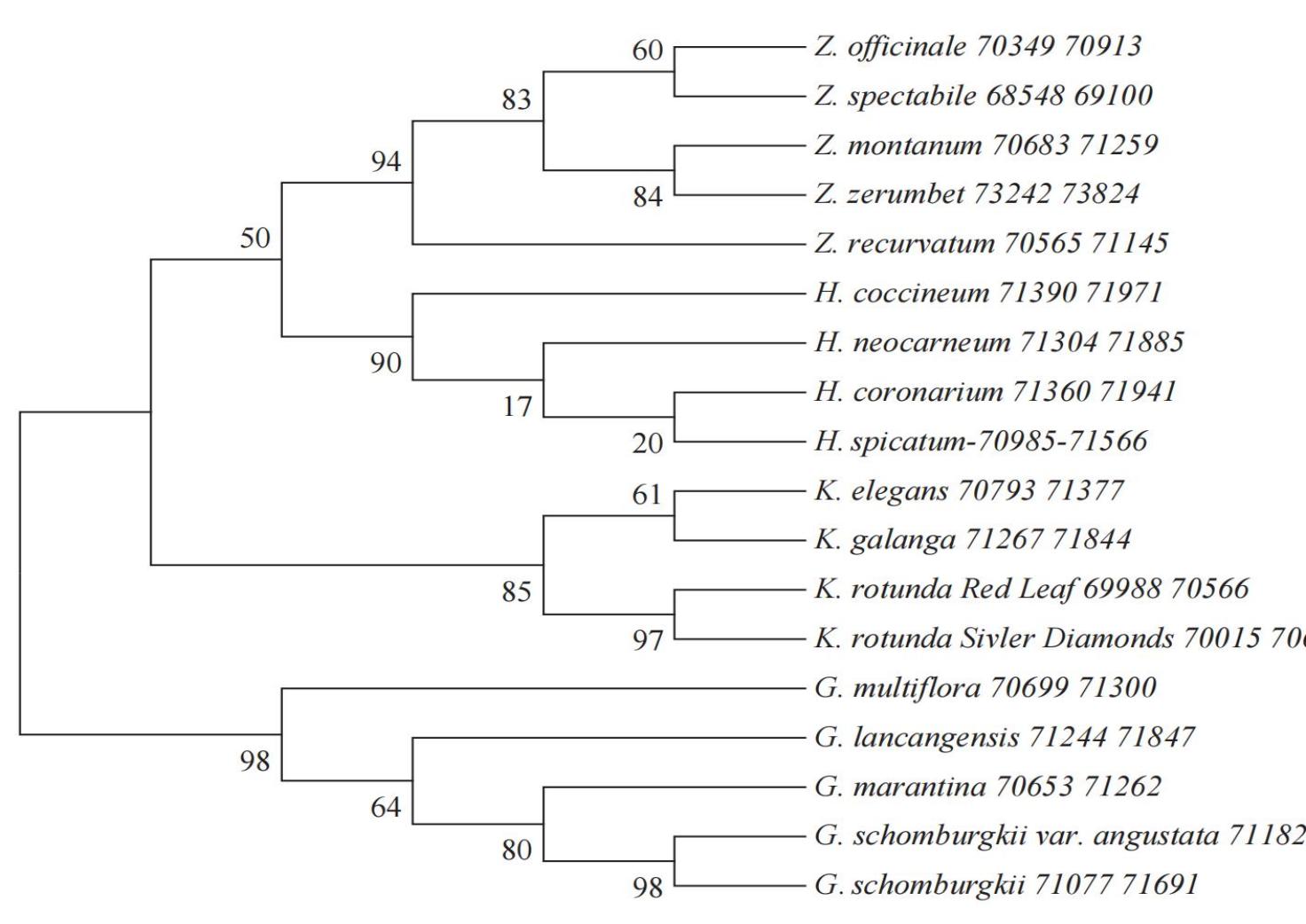


**e**

**Figure S2.** continued.


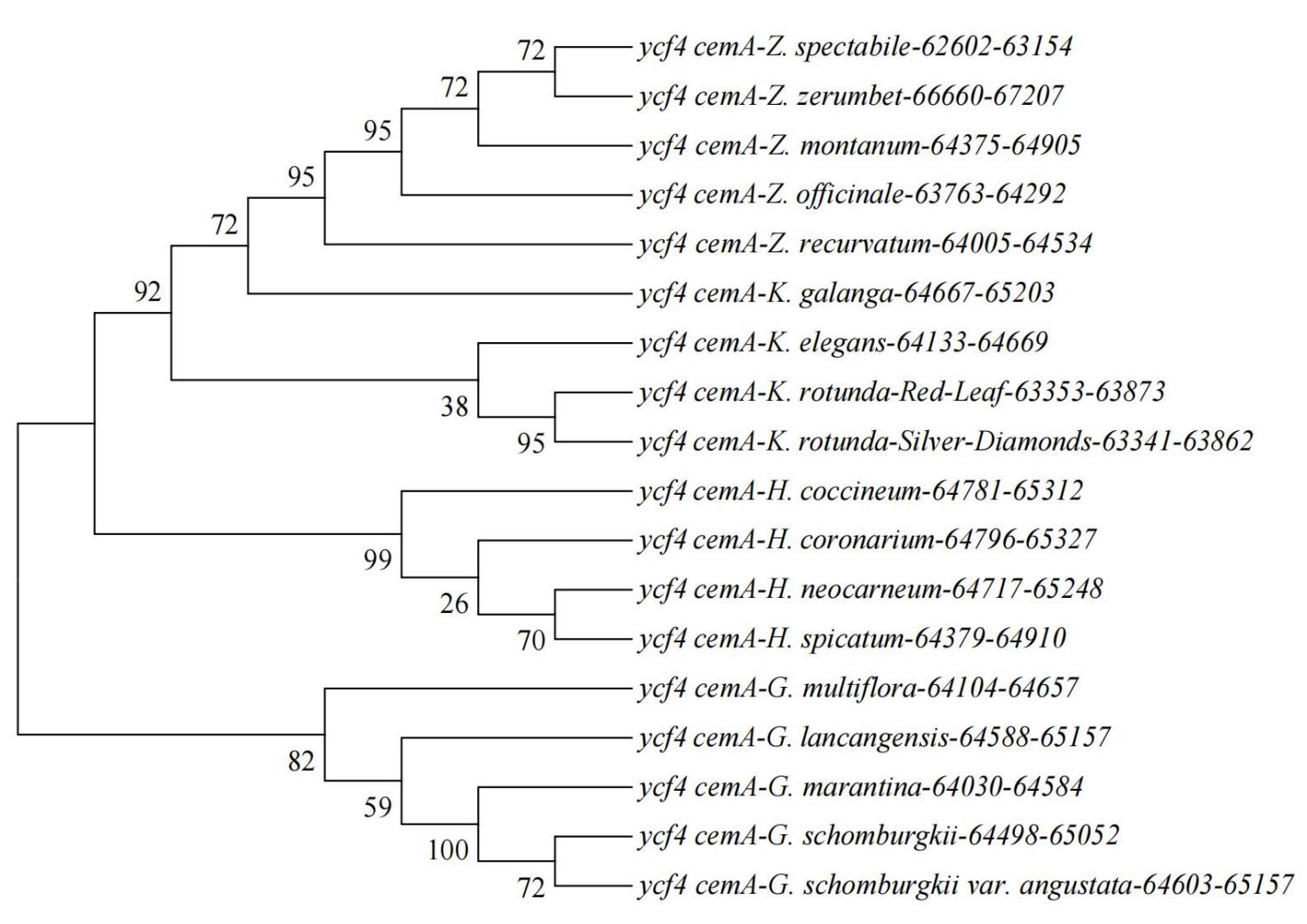


**f**


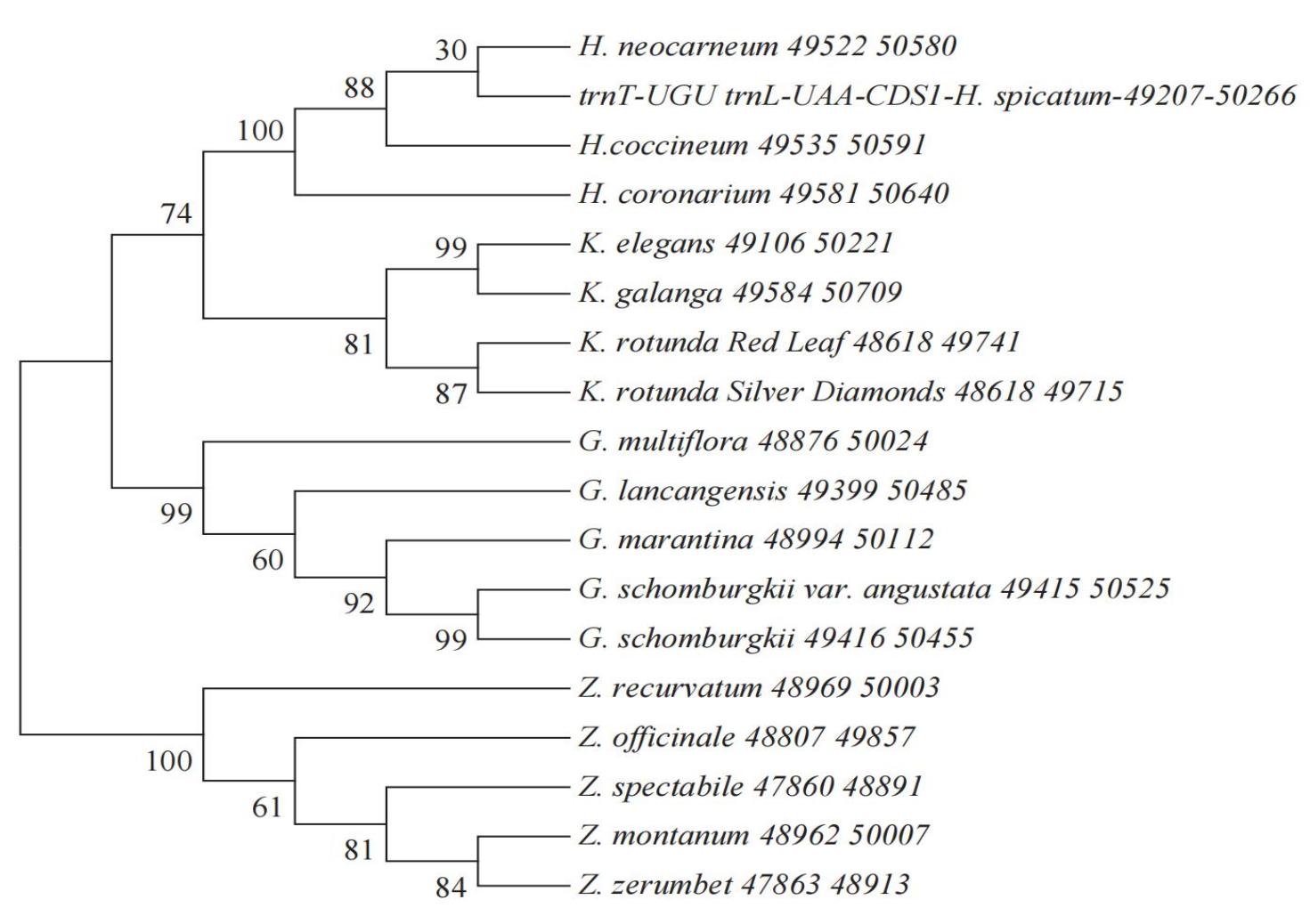


**g**

**Figure S2.** continued.


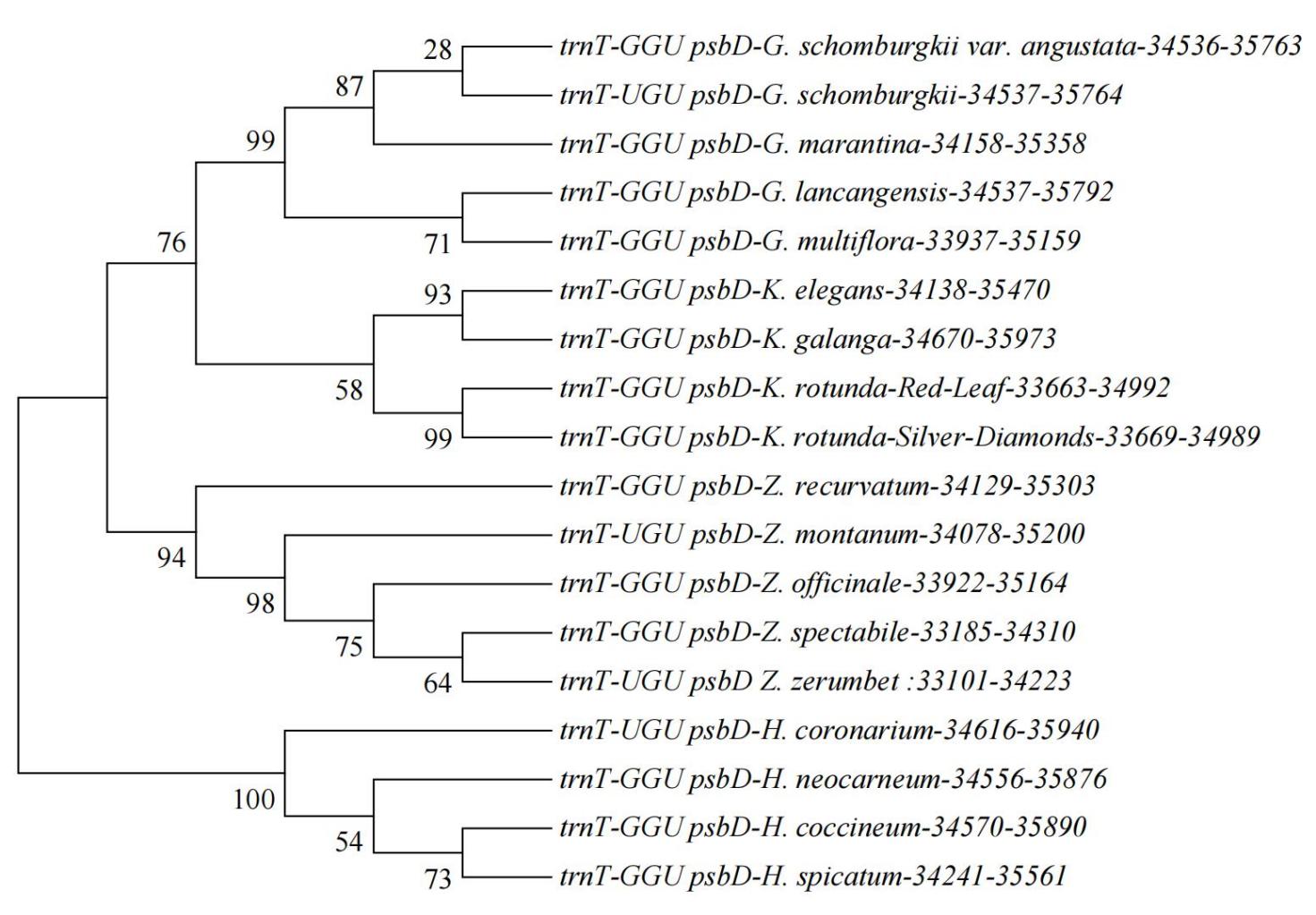


**h**


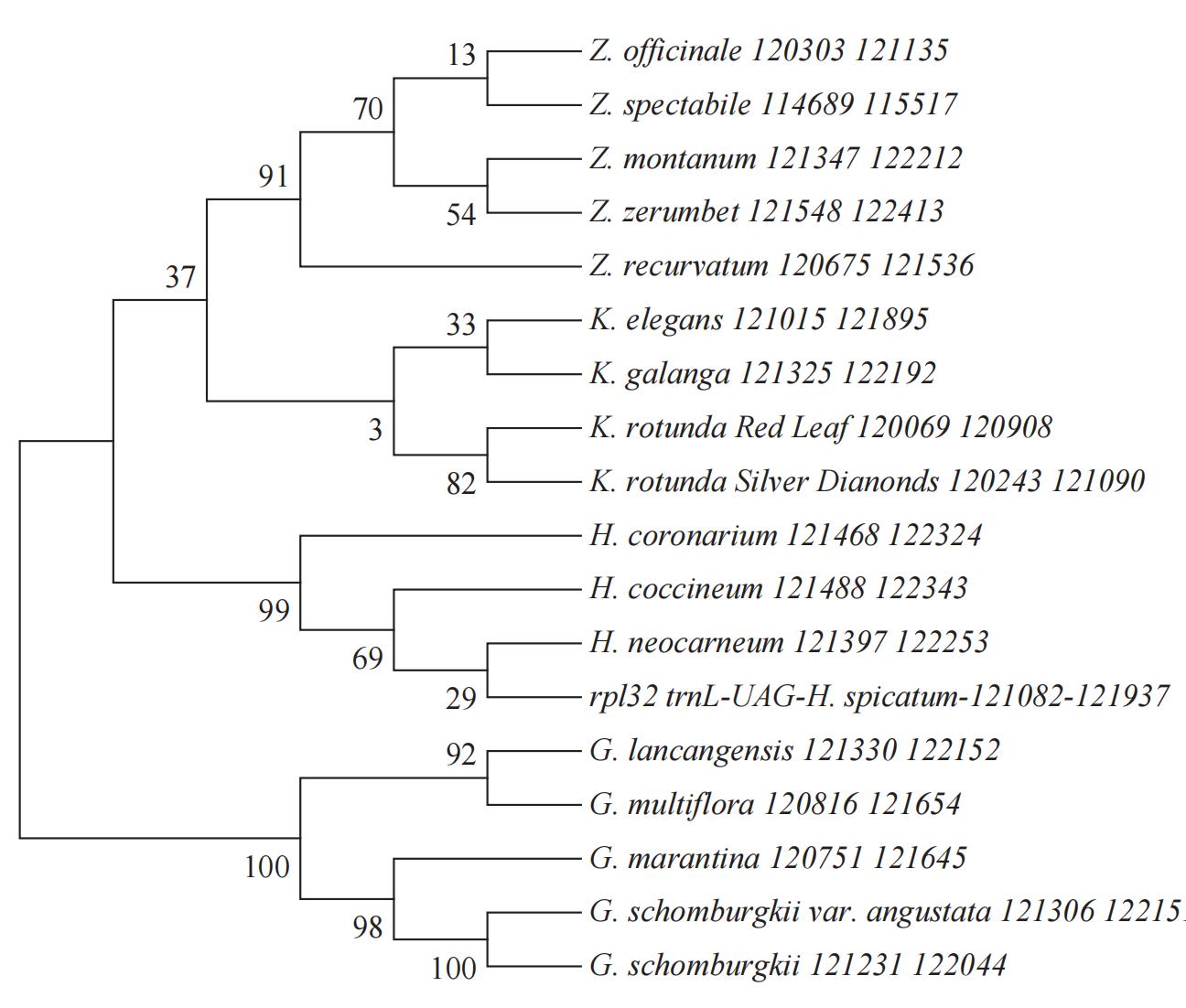


**i**

**Figure S2.** continued.


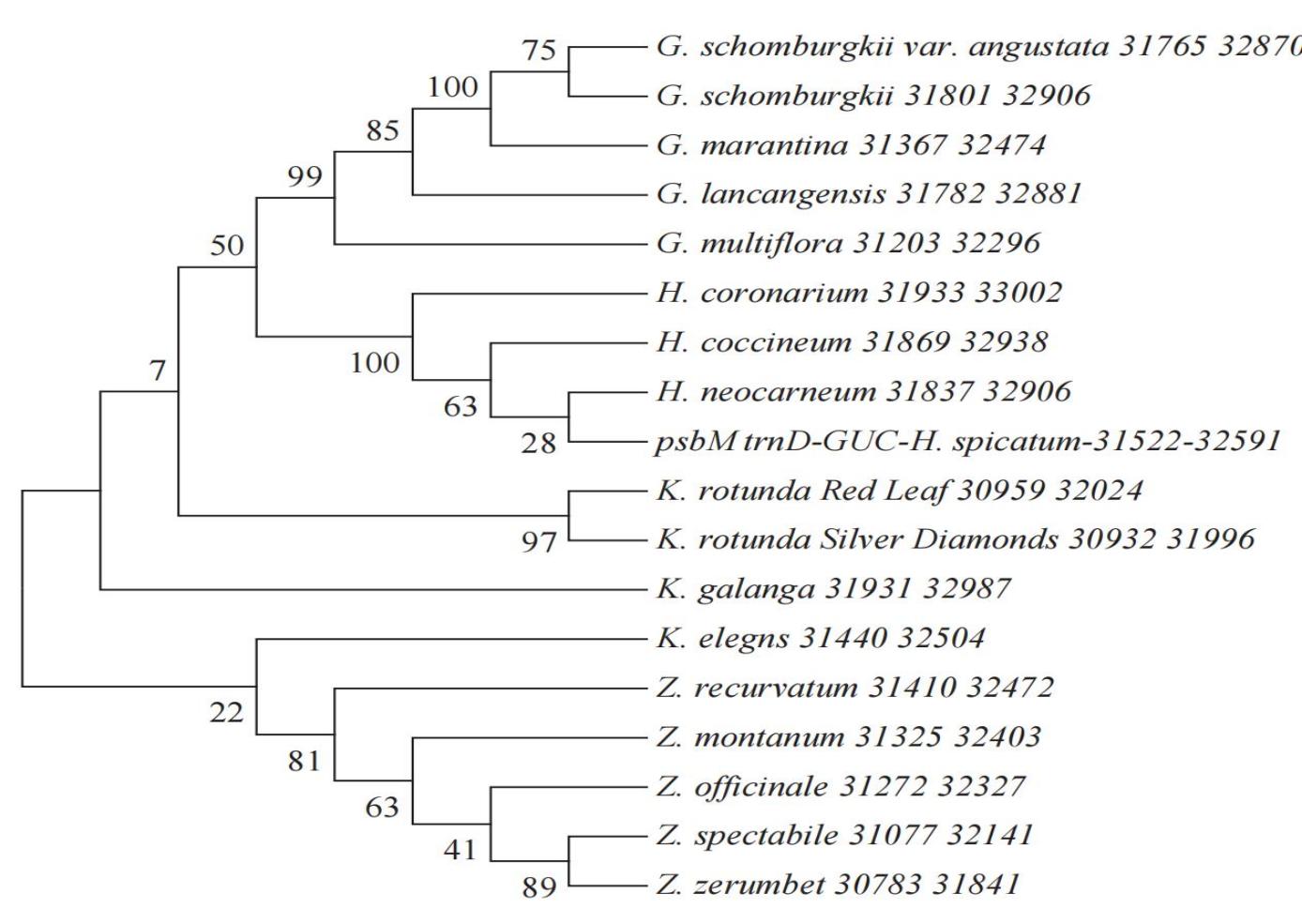


**j**


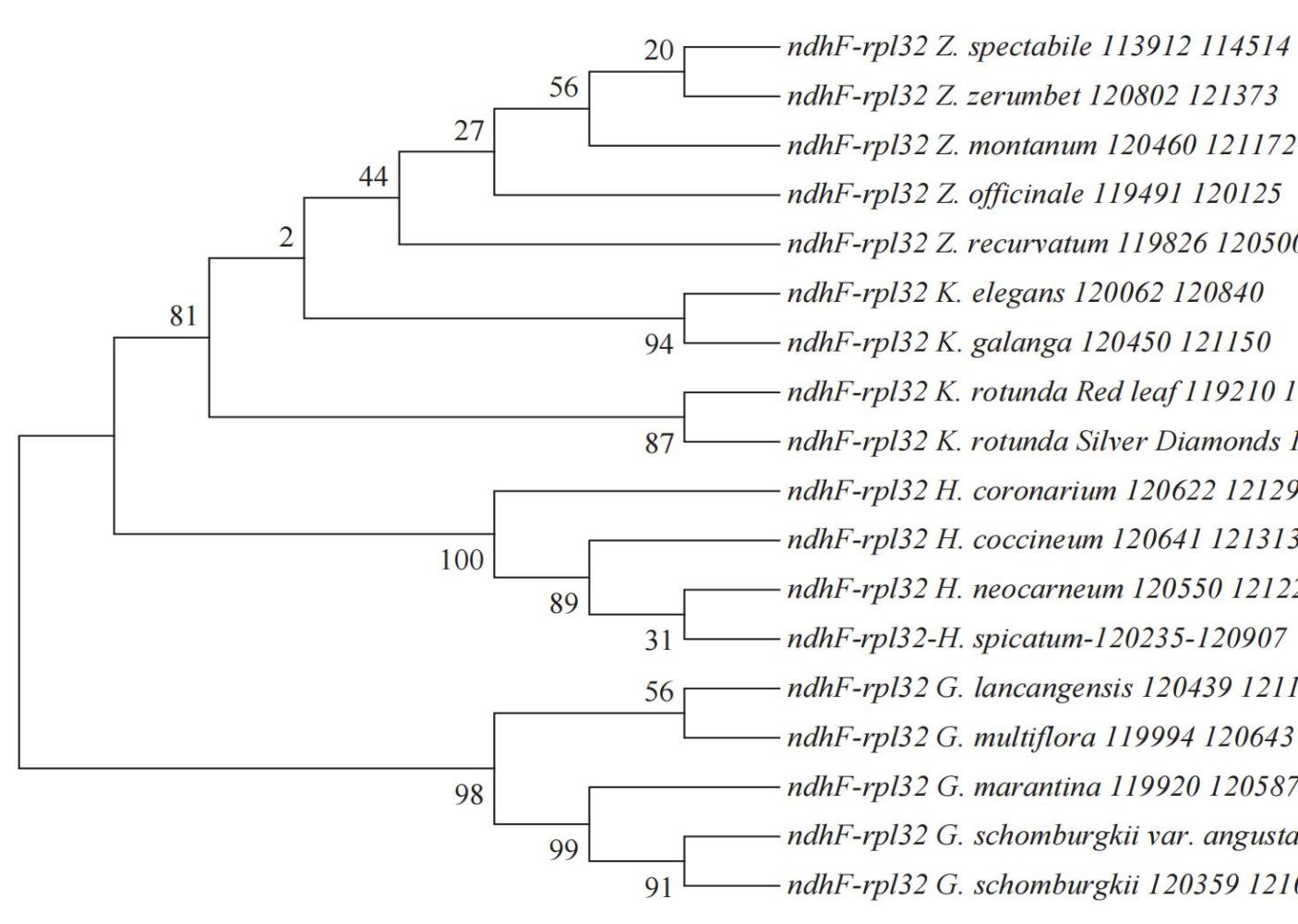


**k**

**Figure S2.** continued.


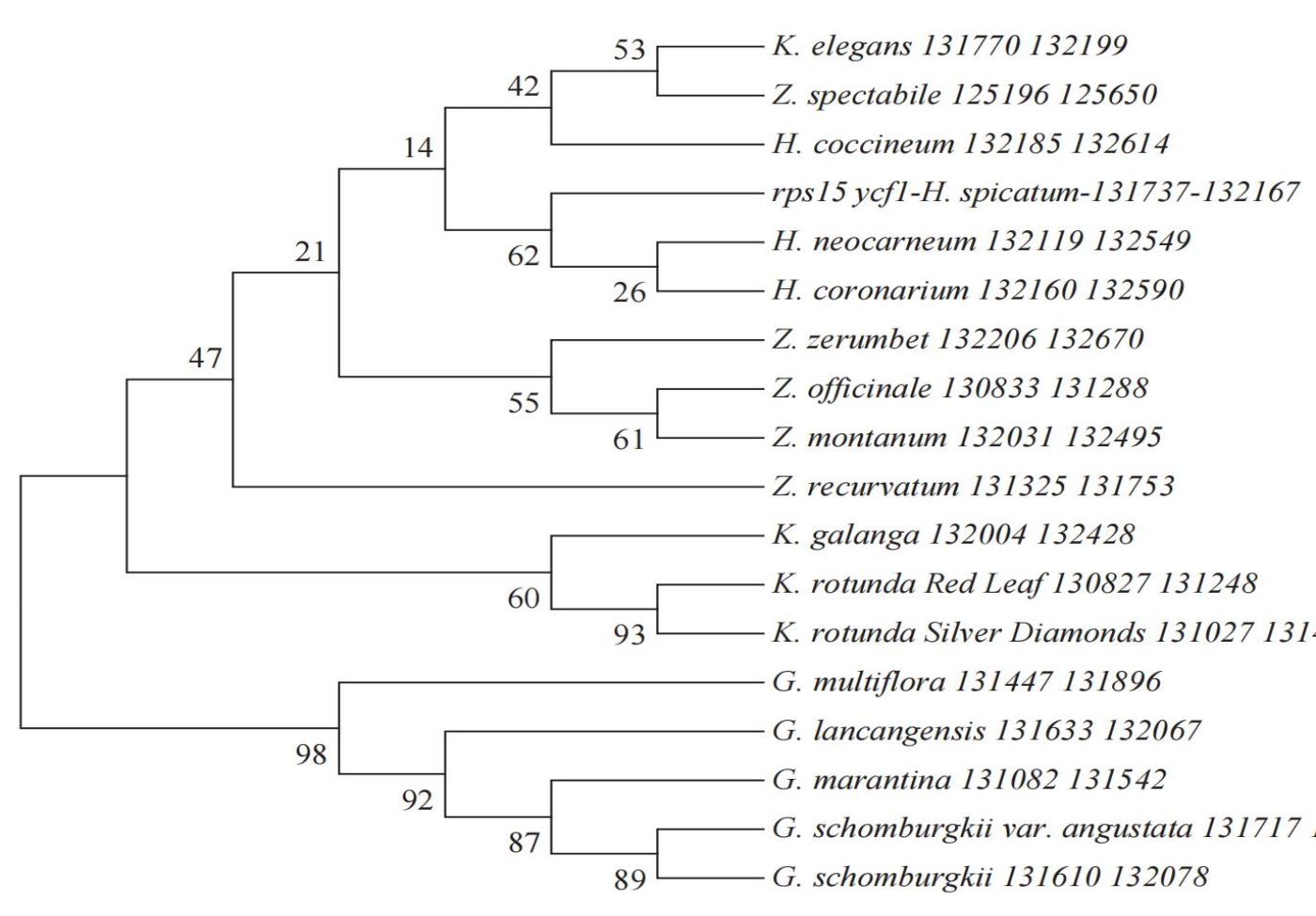


**l**


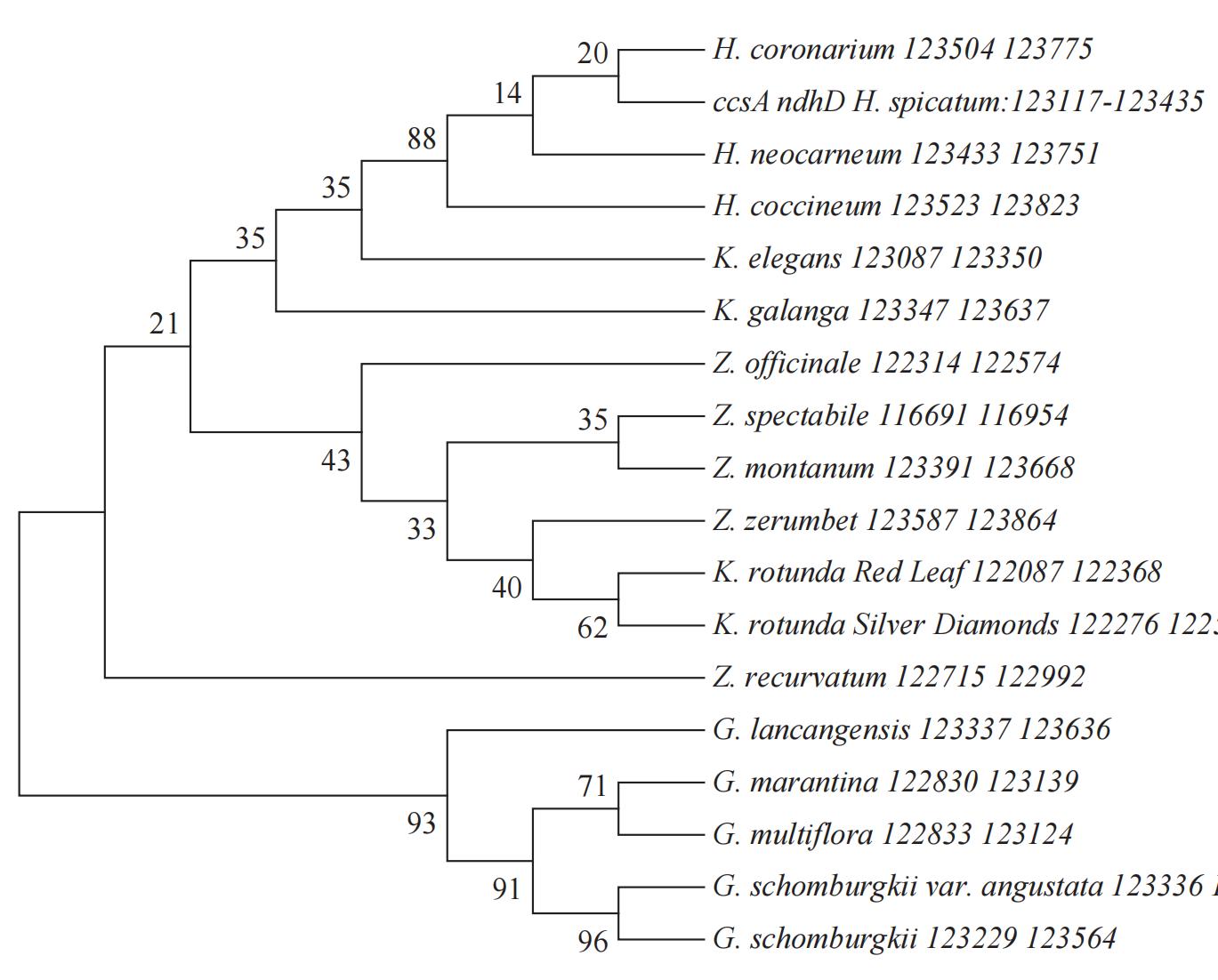


**m**

**Figure S2.** continued.
