# Supplementary material for: Molecular evolution of chloroplast genomes in subfamily Zingiberoideae (Zingiberaceae)
Source: BMC Plant Biol. 2021 Nov 23;21:558. doi: 10.1186/s12870-021-03315-9 (PMC8611967; doi:10.1186/s12870-021-03315-9)
Supplement: Supplementary file 17 — Additional file 17: Figure S3. Molecular phylogenetic tree based on the SNPs from 56 chloroplast genomes of family Zingiberaceae using Bayesian inference. The numbers at the nodes were Bayesian inference posterior probabilities. All nodes of the tree were supported by 1.00 Bayesian inference posterior probability. The branch length was proportional to the inferred divergence level and the scale bar indicated the number of inferred nucleic acids substitutions per site. C. indica, C. pulverulentus and C. viridis were used as the outgroups. [file 12870_2021_3315_MOESM17_ESM.docx]

**
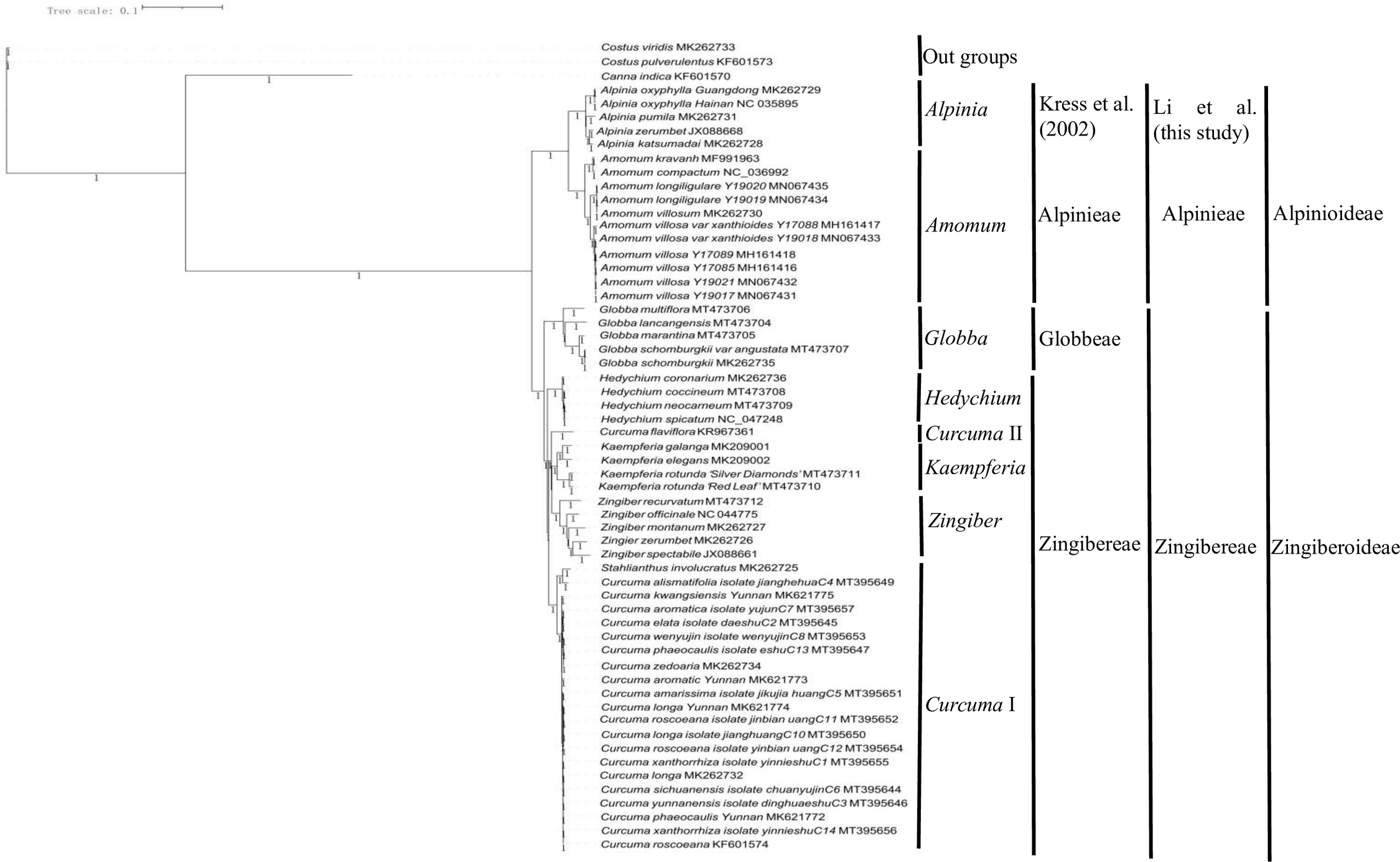
**

**Figure S3.** Molecular phylogenetic tree based on the SNPs from 56 chloroplast genomes of family Zingiberaceae using Bayesian inference. The numbers at the nodes were Bayesian inference posterior probabilities. All nodes of the tree were supported by 1.00 Bayesian inference posterior probability. The branch length was proportional to the inferred divergence level and the scale bar indicated the number of inferred nucleic acids substitutions per site. *C. indica*, *C. pulverulentus* and *C. viridis* were used as the outgroups.
